# The PAX5‐JAK2 translocation acts as dual‐hit mutation that promotes aggressive B‐cell leukemia via nuclear STAT5 activation

**DOI:** 10.15252/embj.2021108397

**Published:** 2022-02-14

**Authors:** Sabine Jurado, Anna S Fedl, Markus Jaritz, Daniela Kostanova‐Poliakova, Stephen G Malin, Charles G Mullighan, Sabine Strehl, Maria Fischer, Meinrad Busslinger

**Affiliations:** ^1^ Research Institute of Molecular Pathology (IMP) Vienna Biocenter (VBC) Vienna Austria; ^2^ Laboratory of Immunobiology Department of Medicine Solna Karolinska Institute Stockholm Sweden; ^3^ Department of Pathology St Jude Children’s Research Hospital Memphis TN USA; ^4^ St. Anna Children’s Cancer Research Institute (CCRI) Vienna Austria; ^5^ Present address: Boehringer Ingelheim RCV GmbH Vienna Austria

**Keywords:** B‐cell acute lymphoblastic leukemia, dual‐hit mutation, mouse *Pax5*‐*Jak2* knock‐in model, nuclear function of Pax5‐Jak2, *PAX5‐JAK2* rearrangement, Chromatin, Transcription & Genomics, Immunology

## Abstract

While *PAX5* is an important tumor suppressor gene in B‐cell acute lymphoblastic leukemia (B‐ALL), it is also involved in oncogenic translocations coding for diverse PAX5 fusion proteins. *PAX5‐JAK2* encodes a protein consisting of the PAX5 DNA‐binding region fused to the constitutively active JAK2 kinase domain. Here, we studied the oncogenic function of the PAX5‐JAK2 fusion protein in a mouse model expressing it from the endogenous *Pax5* locus, resulting in inactivation of one of the two *Pax5* alleles. *Pax5* ^Jak2/+^ mice rapidly developed an aggressive B‐ALL in the absence of another cooperating exogenous gene mutation. The DNA‐binding function and kinase activity of Pax5‐Jak2 as well as IL‐7 signaling contributed to leukemia development. Interestingly, all *Pax5* ^Jak2/+^ tumors lost the remaining wild‐type *Pax5* allele, allowing efficient DNA‐binding of Pax5‐Jak2. While we could not find evidence for a nuclear role of Pax5‐Jak2 as an epigenetic regulator, high levels of active phosphorylated STAT5 and increased expression of STAT5 target genes were seen in *Pax5* ^Jak2/+^ B‐ALL tumors, implying that nuclear Pax5‐Jak2 phosphorylates STAT5. Together, these data reveal Pax5‐Jak2 as an important nuclear driver of leukemogenesis by maintaining phosphorylated STAT5 levels in the nucleus.

## Graphical abstract

A mouse model of the oncogenic *PAX5‐JAK2* translocation reveals promotion of aggressive B‐ALL not via transcriptional or epigenetic alterations, but via increased phosphorylation and activation of the JAK2 target STAT5.

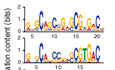

## Introduction

Acute lymphoblastic leukemia is the most common pediatric cancer and, in the majority of cases, originates within the B cell lineage (Hunger & Mullighan, 2015). Genome‐wide studies demonstrated that 60% of all B‐cell precursor acute lymphoblastic leukemia (B‐ALL) cases carry genetic alterations in genes coding for regulators of B cell development, with the most commonly affected transcription factor genes being *PAX5*, *EBF1,* and *IKZF1* (Ikaros) (Kuiper *et al*, 2007; Mullighan *et al*, 2007, 2008). Mutations leading to aberrant activation of tyrosine kinase and cytokine receptor signaling were also identified in about 15% of all B‐ALL cases, which are collectively referred to as Philadelphia chromosome‐like (Ph‐like) B‐ALLs and have a poor prognosis (Roberts *et al*, 2012, 2014; Ofran & Izraeli, 2017). One Ph‐like B‐ALL subtype is characterized by the *PAX5‐JAK2* rearrangement (Nebral *et al*, 2009; Roberts *et al*, 2012), which we have studied here with regard to its molecular and oncogenic function in a mouse model.

The transcription factor Pax5 is an essential regulator of B cell commitment and development (Nutt *et al*, 1999; Horcher *et al*, 2001; Medvedovic *et al,* 2001). It acts as a transcriptional repressor to suppress B‐lineage‐inappropriate genes (Delogu *et al*, 2006) and functions as an activator to induce gene expression required for B cell development and function (Schebesta *et al*, 2007; Revilla‐i‐Domingo *et al*, 2012). Notably, mature B cells upon conditional loss of Pax5 cannot only be converted into functional T cells via dedifferentiation to uncommitted bone marrow progenitor cells, but also give rise to an aggressive progenitor cell leukemia (Cobaleda *et al*, 2007). Hence, Pax5 maintains B cell identity throughout B‐lymphopoiesis and functions as a tumor suppressor in the B‐lymphoid lineage.

*PAX5* was identified as a haploinsufficient tumor suppressor gene in human B‐ALL, as heterozygous *PAX5* deletions, rearrangements, and loss‐of‐function mutations are present in one third of all cases (Kuiper *et al*, 2007; Mullighan *et al*, 2007). *PAX5* translocations occur at a frequency of 2.5% in human B‐ALLs and are currently known to involve 28 different partner genes, generating novel PAX5 fusion proteins (Gu *et al*, 2019). The different partner genes code for proteins of diverse functions such as transcription factors (exemplified by ETV6), signal transducers (JAK2), chromatin regulators (BRD1), structural proteins (ELN), and proteins of unknown function (NOL4L) (Cazzaniga *et al*, 2001; Nebral *et al*, 2009; Coyaud *et al*, 2010). The PAX5 fusion proteins contain the N‐terminal DNA‐binding paired domain, but lack the potent C‐terminal transactivation domain of PAX5 (Nebral *et al*, 2009; Coyaud *et al*, 2010) and were therefore thought to act as dominant‐negative proteins to prevent genomic DNA binding of the full‐length PAX5 protein expressed from the second allele (Kawamata *et al*, 2012; Fortschegger *et al*, 2014). However, we could demonstrate in a mouse model that the PAX5‐ETV6 fusion protein does not interfere with the expression of regulated Pax5 target genes and hence does not act as a dominant‐negative protein (Smeenk *et al*, 2017). Instead, PAX5‐ETV6 functions as a potent oncoprotein to promote B‐ALL development in combination with loss of the tumor suppressors Cdkn2a and Cdkn2b (Smeenk *et al*, 2017). Similarly, heterozygous loss of *Pax5* is not sufficient for tumor formation, as it additionally requires constitutive activation (ca) of STAT5 for leukemia development in transgenic *caStat5b Pax5* ^+/−^ mice (Heltemes‐Harris *et al*, 2011). Hence, heterozygous *PAX5* alterations promote B‐ALL development in cooperation with a second oncogenic “hit.”

The Janus kinase 2 (JAK2) belongs to a family of nonreceptor tyrosine kinases that mediate signal transduction downstream of many cytokine and growth hormone receptors, regulating hematopoiesis, immunity, growth, and development (Chen *et al*, 2012; Villarino *et al*, 2017). Upon signaling, JAK2 phosphorylates STAT transcription factors in the cytoplasm, which promotes their dimerization and translocation to the nucleus, where they control the expression of genes involved in cell survival, differentiation, and metabolism (Malin *et al*, 2010; Villarino *et al*, 2017; de Araujo *et al*, 2019). In addition to this canonical JAK‐STAT signaling function, JAK2 was also shown to be present in the nucleus and to directly phosphorylate histone H3 on tyrosine 41 (H3Y41ph), which, in turn, prevents interaction of the heterochromatin protein 1α (HP1α) with H3, thus leading to the activation of oncogenes such as *Lmo2* and *Myc* (Dawson *et al*, 2009; Rui *et al*, 2010). Hence, these studies uncovered a second role of JAK2 as an “epigenetic writer” that stimulates expression of leukemogenic genes. JAK2 has directly been implicated in the development of Ph‐like B‐ALL by activating mutations, which are predominantly located in its autoinhibitory pseudokinase domain (JH2) (Chen *et al*, 2012). *JAK2* also participates in translocations with at least 22 different partner genes, which all contain the catalytically active JAK2 kinase domain (JH1) as a common denominator (Nebral *et al*, 2009; Chen *et al*, 2012; Roberts *et al*, 2012; Akkari *et al*, 2020).

The *PAX5*‐*JAK2* rearrangement codes for a fusion protein consisting of the DNA‐binding paired domain of PAX5 fused to only the kinase domain (JH1) of JAK2 (Nebral *et al*, 2009) (Appendix Fig S1A). As shown by detailed characterization in transfected cell lines, PAX5‐JAK2 is a monomeric nuclear protein that can bind Pax5 recognition sequences and possesses constitutive kinase activity. Consequently, PAX5‐JAK2 activates STAT5 by phosphorylation, which likely induces a STAT5‐dependent gene program (Schinnerl *et al*, 2015). Moreover, JAK2 inhibitors efficiently block the constitutive kinase activity of PAX5‐JAK2 in transfected cells, suggesting that these inhibitors may be beneficial for the treatment of PAX5‐JAK2^+^ B‐ALL (Roberts *et al*, 2014; Schinnerl *et al*, 2015). As the *PAX5*‐*JAK2* rearrangement inactivates one functional *PAX5* allele, resulting in haploinsufficiency, and simultaneously leads to STAT5 activation, it may function as a dual‐hit mutation to promote aggressive B‐ALL. However, the function of PAX5‐JAK2 in B cell development and leukemogenesis has not yet been investigated in an *in vivo* model system.

Here, we have generated a mouse model that expresses the PAX5‐JAK2 protein under the control of the *Pax5* locus. *Pax5* ^Jak2/+^ mice exhibited normal B cell development up to 3 weeks of age, but thereafter rapidly developed an aggressive B‐ALL tumor in the absence of another cooperating exogenous gene mutation. The DNA‐binding function and kinase activity of Pax5‐Jak2 both contributed to leukemia development, as evidenced by mutation of the paired domain of Pax5 or the catalytic center of Jak2. Unexpectedly, the wild‐type *Pax5* allele was lost in all *Pax5* ^Jak2/+^ B‐ALLs by acquired uniparental disomy, which facilitated efficient binding of Pax5‐Jak2 to its genomic recognition sequences, thus pointing to an important oncogenic function of the fusion protein in the nucleus. While we could not find evidence for an epigenetic role of Pax5‐Jak2 in the nucleus, STAT5 was highly phosphorylated in the earliest pre‐leukemic B220^low^ B cells of 4‐week‐old mice. Consistent with this finding, activated STAT5 target genes were upregulated in *Pax5* ^Jak2/+^ B‐ALLs. Together, these data indicate that the constitutively active Pax5‐Jak2 kinase maintains active STAT5 at high levels in the nucleus, thus leading to continuous expression of STAT5 target genes in *Pax5* ^Jak2/+^ B‐ALL cells.

## Results

### Pax5‐Jak2 expression from the *Pax5* locus leads to development of an aggressive B‐ALL

**Figure 1:**
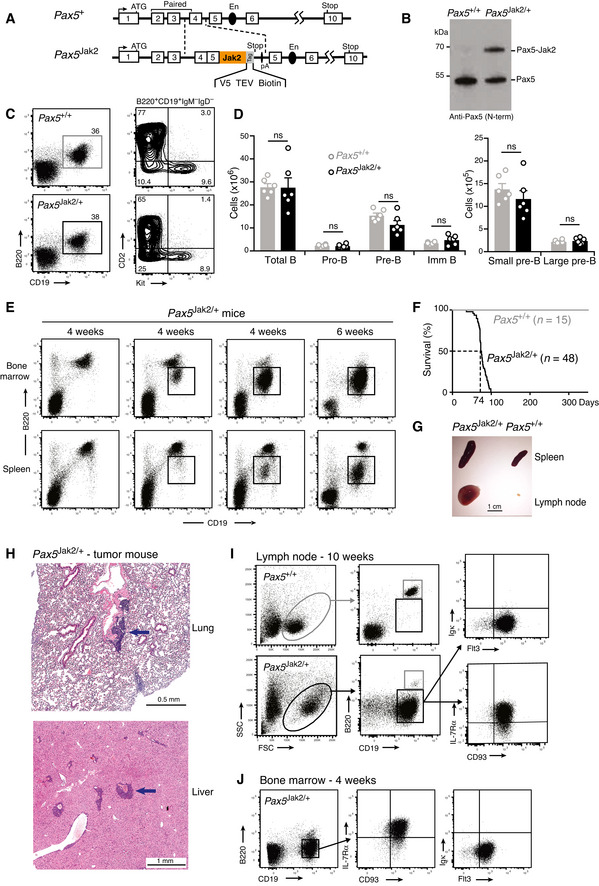
B‐ALL development in *Pax5* ^Jak2/+^ mice Schematic diagram of the *Pax5* ^Jak2^ allele. Human cDNA sequences, starting in *PAX5* exon 4 and encoding the remaining PAX5‐JAK2 protein, were inserted in frame into exon 4 of the mouse *Pax5* locus to generate the *Pax5* ^Jak2^ allele (Appendix Fig S1B). The C‐terminal tag sequence (grey) contains an epitope for the V5 antibody, two cleavage sites for the TEV protease, and a biotin acceptor sequence (Biotin). A black oval denotes the B cell‐specific enhancer (En) in intron 5 (Decker *et al*, 2009). pA, polyadenylation site. Notably, the human and mouse Pax5 protein sequences encoded from exon 1 to exon 5 contain only one amino acid substitution (human Ser13 vs. mouse Ile13 in exon 1) located upstream of the paired domain (Adams *et al*, 1992).Expression of the Pax5 protein in *Pax5* ^Jak2/+^ pro‐B cells. Nuclear extracts of short‐term cultured pro‐B cells from 3‐week‐old *Pax5* ^Jak2/+^ and *Pax5* ^+/+^ mice were analyzed by immunoblot analysis with an anti‐Pax5 antibody recognizing the N‐terminal paired domain. The size of marker proteins is indicated in kilodaltons (kDa) to the left.Flow‐cytometric analysis of bone marrow cells from the two hindlegs of 3‐week‐old *Pax5* ^+/+^ and *Pax5* ^Jak2/+^ mice. The percentage of the cells in each gate or quadrant is indicated.Absolute cell numbers of the indicated cell types were determined by flow‐cytometric analysis of the bone marrow from 3‐week‐old *Pax5* ^+/+^ and *Pax5* ^Jak2/+^ mice. Average cell numbers are shown with SEM and were statistically analyzed by multiple *t*‐tests (unpaired two‐tailed with Holm‐Šídák's correction); ns, not significant (*P* > 0.05). See Methods section for flow‐cytometric definition of the different cell types. One of 3 independent experiments is shown.Flow‐cytometric analysis of bone marrow cells and splenocytes from *Pax5* ^Jak2/+^ mice at the age of 4 or 6 weeks. The newly emerging B220^low^ B cells are highlighted by black boxes.Kaplan‐Meier survival analysis of *Pax5* ^Jak2/+^ (black) and control *Pax5* ^+/+^ (grey) mice. *n*, number of mice analyzed. A *P* value of < 0.0001 was determined for the survival curves by statistical analysis with the log‐rank (Mantel‐Cox) test.Size comparison of the spleen and lymph nodes from a control *Pax5* ^+/+^ mouse and a *Pax5* ^Jak2/+^ tumor mouse.Eosin‐hematoxylin‐stained sections of the lung and liver of a *Pax5* ^Jak2/+^ tumor mouse. Infiltrating and blasting tumor cells are indicated by an arrow.Flow‐cytometric analysis of lymph node cells from a control *Pax5* ^+/+^ mouse and a 10‐week‐old *Pax5* ^Jak2/+^ tumor mouse.Flow‐cytometric analysis of B220^low^CD19^+^ B cells from the bone marrow of a 4‐week‐old *Pax5* ^Jak2/+^ mouse. Source data are available online for this figure.

To study the role of PAX5‐JAK2 (Appendix Fig S1A) in B‐ALL development, we used ES cell targeting to generate a mouse model by inserting human cDNA sequences, starting with exon 4 and encoding the remaining PAX5‐JAK2 protein, into the mouse *Pax5* locus to recapitulate the corresponding human rearrangement as closely as possible (Fig 1A and Appendix Fig S1B and C). Additionally, we inserted an IRES‐luciferase indicator gene downstream of the *Jak2* sequence and turned the endogenous *Pax5* exon 4 into a *lox*P‐stop‐*lox*P (LSL) cassette by the insertion of a stop codon together with six polyadenylation sequences to generate the *Pax5* ^LSL‐Jak2‐Luc^ allele, which expresses the DNA‐binding paired domain of Pax5 instead of the full‐length Pax5‐Jak2 protein (Appendix Fig S1B). The *Pax5* ^LSL‐Jak2^ allele was subsequently created by Dre recombinase‐mediated deletion of the IRES‐luciferase gene (Appendix Fig S1B). To enable the expression of the Pax5‐Jak2 fusion, we eliminated the LSL cassette by ubiquitous Cre expression from the *Meox2* locus (Tallquist & Soriano, 2000) in *Meox2*‐Cre *Pax5* ^LSL‐Jak2/+^ or *Meox2*‐Cre *Pax5* ^LSL‐Jak2‐Luc/+^ mice, which will be thereafter referred to as *Pax5* ^Jak2/+^ or *Pax5* ^Jak2‐Luc/+^ mice, respectively. The LSL cassette was efficiently deleted in cells of *Pax5* ^Jak2/+^ and *Pax5* ^Jak2‐Luc/+^ mice, as shown by PCR analysis (Appendix Fig S1D). Immunoblot analysis of nuclear extracts with an anti‐Pax5 antibody recognizing the N‐terminal paired domain demonstrated that the Pax5‐Jak2 and wild‐type Pax5 proteins were similarly expressed in pro‐B cells of 3‐week‐old *Pax5* ^Jak2/+^ mice (Fig 1B and Appendix Fig S1E and F). These results therefore identified the *Pax5* ^Jak2/+^ mouse as a valid model for studying the developmental and leukemogenic role of Pax5‐Jak2 in B cells.

Flow‐cytometric analysis of B cell development in 3‐week‐old mice revealed that total B, pro‐B, large and small pre‐B, as well as immature B cells were present at similar numbers in the bone marrow of *Pax5* ^Jak2/+^ and control *Pax5* ^+/+^ mice (Fig 1C and D). These data therefore indicate that heterozygous expression of Pax5‐Jak2 had no apparent effect on B cell development in young mice.

The first sign of aberrant B cell development appeared in the bone marrow of *Pax5* ^Jak2/+^ mice at around 4 weeks of age with the emergence of B220^low^ B cells in some of these mice (Fig 1E). At 6 weeks of age, most B lymphocytes in the bone marrow were B220^low^ and proved to be tumorigenic, as their transplantation into wild‐type C57BL/6 mice resulted in tumor development within 72 days (Appendix Fig S1G). With some delay, these tumorigenic B220^low^ B cells also appeared in the spleen (Fig 1E), in agreement with their generation in the bone marrow. The expansion of these tumorigenic B220^low^ B cells led to the rapid death of *Pax5* ^Jak2/+^ mice with a median survival of 74 days, as shown by Kaplan–Meier survival analysis (Fig 1F). Moribund *Pax5* ^Jak2/+^ mice exhibited enlarged lymph nodes and splenomegaly (Fig 1G) as well as infiltration of leukemic cells in other organs such as the lung and liver (Fig 1H). Flow‐cytometric analyses revealed that the leukemic B cells from lymph nodes were blasting, as shown by their large size, and expressed surface markers characteristic of early B lymphopoiesis, such as CD93, IL‐7Rα (CD127), and Flt3 (CD135) (Fig 1I). The cell surface phenotype of the *Pax5* ^Jak2/+^ tumors was B220^low^CD93^+^IL7R^low/+^Flt3^+^Kit^low/+^IgM^low/−^CD2^−^IgD^−^CD21^−^CD23^−^, while the expression of CD19 was variable, with some tumors being positive, negative, or mixed (CD19^+^ to CD19^−^) (Fig 1I and Appendix Fig S1H). Moreover, the *Pax5* ^Jak2/+^ tumor cells from lymph nodes gave rise to overt leukemia within 30 days after transplantation in wild‐type C57BL/6 mice, thus highlighting their aggressive nature (Appendix Fig S1G). Notably, the B220^low^ B cells in the bone marrow of 4‐week‐old *Pax5* ^Jak2/+^ mice already expressed CD93, IL‐7Rα, and Flt3 (Fig 1J) and thus had a similar cell surface phenotype as the B‐ALL tumors in the lymph nodes of *Pax5* ^Jak2/+^ mice (Fig 1I). In summary, these data demonstrate that Pax5‐Jak2 expression from the *Pax5* locus initially did not interfere with normal B cell development in young mice, but then rapidly led to the development of an aggressive B‐ALL tumor.

### The *Pax5* ^Jak2^ allele does not provide any canonical Pax5 function

**Figure 2:**
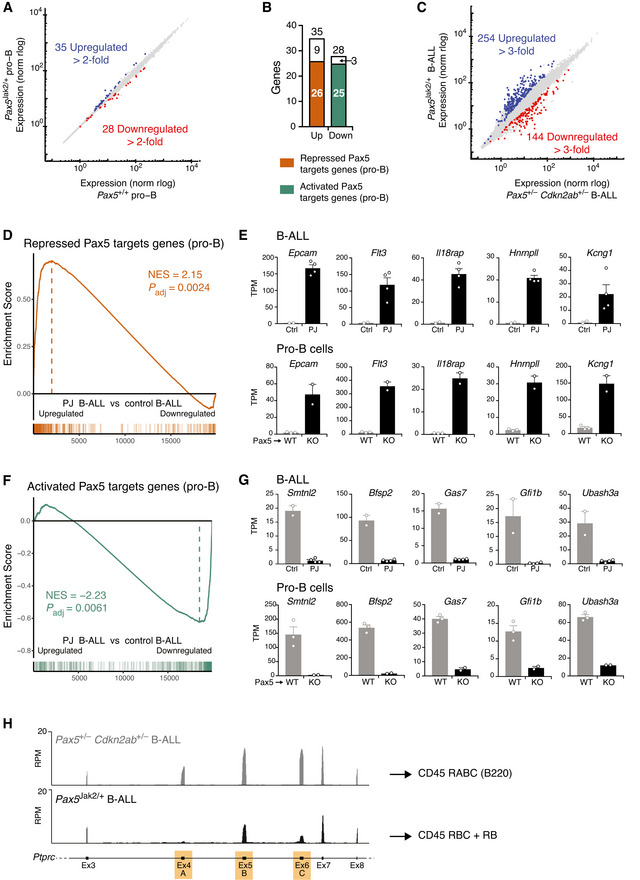
Gene regulation in *Pax5* ^Jak2/+^ pro‐B cells and *Pax5* ^Jak2/+^ B‐ALL cells AScatter plot of gene expression differences between *Pax5* ^Jak2/+^ and *Pax5* ^+/+^ pro‐B cells, which were isolated from the bone marrow of 3‐week‐old mice prior to RNA‐sequencing. The expression data of individual genes (indicated by dots) are plotted as mean normalized rlog (regularized logarithm) values. Genes with an expression difference of > 2‐fold, an adjusted *P* value of < 0.05 and a TPM value of > 5 (in at least one cell type) are colored in blue and red, corresponding to upregulation or downregulation in *Pax5* ^Jak2/+^ pro‐B cells (Dataset EV1).BBar graph indicating how many of the up‐ or down‐regulated genes in *Pax5* ^Jak2/+^ pro‐B cells correspond to repressed (orange) or activated (green) Pax5 target genes identified in pro‐B cells (Appendix Fig S2A).CScatter plot of gene expression differences between *Pax5* ^Jak2/+^ and *Pax5* ^+/−^ *Cdkn2ab* ^+/−^ B‐ALL cells of tumors that were isolated from lymph nodes prior to RNA‐sequencing. Genes with an expression difference of > 3‐fold, an adjusted *P* value of < 0.05 and a TPM value of > 5 (in at least one tumor cell type) are colored in blue and red, corresponding to upregulation or downregulation in *Pax5* ^Jak2/+^ B‐ALL cells (Dataset EV3).D–GGSEA analysis of 327 repressed (D) or 330 activated (F) Pax5 target genes identified in pro‐B cells (Appendix Fig S2A), as compared with the ranked log_2_‐fold gene expression changes in *Pax5* ^Jak2/+^ (PJ) B‐ALLs versus control (Ctrl) *Pax5* ^+/−^ *Cdkn2ab* ^+/−^ B‐ALLs (left). NES, normalized enrichment score. The expression of five genes that are upregulated in *Pax5* ^Jak2/+^ B‐ALL cells (upper row) and *Pax5* ^−/−^ (KO) pro‐B cells (lower row) is shown in (E). Likewise, the expression of five genes that are upregulated in control B‐ALL cells (upper row) and *Pax5* ^+/+^ (WT) pro‐B cells (lower row) is shown in (G). TPM, transcripts per million. Mean TPM values with SEM are shown for the following RNA‐seq experiments: 3 (WT pro‐B), 2 (KO pro‐B), 2 (Ctrl B‐ALL), and 4 (PJ B‐ALL).HExpression of the *Ptprc* (CD45) gene from exon 3 to exon 8, as determined by RNA‐seq of *Pax5* ^Jak2/+^ and *Pax5* ^+/−^ *Cdkn2ab* ^+/−^ B‐ALL cells. The alternatively spliced exons 4 (A), 5 (B), and 6 (C) are indicated in orange in the respective exon‐intron structure of the *Ptprc* gene. *Ptprc* transcripts of B220^+^ *Pax5* ^+/−^ *Cdkn2ab* ^+/−^ B‐ALL cells contain all three exons, thus giving rise to expression of the CD45 isoform RABC (known as B220). In contrast, reads at exon 4 are barely detectable and reads at exon 6 are strongly reduced in the mRNA of *Pax5* ^Jak2/+^ B‐ALL cells, which likely gives rise to the CD45 isoforms RBC and RB (see Appendix Fig S2I).

To gain insight into the role of Pax5‐Jak2 in early B cell development, we performed RNA‐sequencing (RNA‐seq) with *ex vivo* sorted pro‐B cells (CD19^+^B220^+^Kit^+^CD2^−^IgM^−^) from the bone marrow of *Pax5* ^Jak2/+^ and control *Pax5* ^+/+^ mice at the age of 3 weeks, before leukemic B220^low^ cells were detected. Differentially expressed genes were identified by an expression difference of > 2‐fold, an adjusted *P* value of < 0.05 and an expression value of > 5 TPM in at least one of the two cell types (Dataset EV1). Very few gene expression changes were observed between *Pax5* ^Jak2/+^ and *Pax5* ^+/+^ pro‐B cells. Only 35 and 28 genes were up‐ or down‐regulated, respectively, in *Pax5* ^Jak2/+^ pro‐B cells compared with control *Pax5* ^+/+^ pro‐B cells (Fig 2A), consistent with the absence of a B cell developmental phenotype in *Pax5* ^Jak2/+^ mice (Fig 1C and D). For comparative analyses, we identified 331 activated and 327 repressed Pax5 target genes with an expression difference of > 3‐fold by performing RNA‐seq analysis with *ex vivo* sorted *Pax5* ^−/−^ and *Pax5* ^+/+^ pro‐B cells as well as Bio‐ChIP‐seq analysis with *ex vivo* sorted *Pax5* ^Bio/Bio^ *Rag2* ^−/−^ pro‐B cells (Appendix Fig S2A and Dataset EV2; see Appendix Supplementary Methods). Comparison of the differentially expressed genes identified in *Pax5* ^Jak2/+^ pro‐B cells with these regulated Pax5 target genes revealed that 26 (74%) of the 35 upregulated genes corresponded to repressed Pax5 target genes, while 25 (89%) of the 28 downregulated genes qualified as activated Pax5 target genes (Fig 2B and Dataset EV1). Interestingly, a previous RNA‐seq comparison of *Pax5* ^+/−^ and *Pax5* ^+/+^ pro‐B cells identified a similarly low number of Pax5‐regulated genes (Smeenk *et al*, 2017). Together, these data suggest that the *Pax5* ^Jak2^ allele behaves like a *Pax5* null allele with regard to Pax5 function.

To corroborate this finding, we generated *Pax5* ^Jak2/−^ and control *Pax5* ^Prd/−^ mice, which were unable to generate CD19^+^ B cells in the bone marrow (Appendix Fig S2B), suggesting that the Jak2 kinase domain does not provide transcriptional activity to the Pax5‐Jak2 fusion protein. Importantly, B cell development in both mouse strains was arrested at an uncommitted B220^+^CD19^−^ progenitor cell stage, expressing Pax5‐Jak2 or the Pax5 paired domain (Prd) from the *Pax5* locus (Appendix Fig S2C and D). We conclude therefore that Pax5‐Jak2 does not function as a transcriptional regulator, as the Jak2 kinase domain cannot substitute for the loss of the central and C‐terminal Pax5 sequences encoding a potent transactivation domain (Dörfler & Busslinger, 1996).

### Pax5‐dependent gene expression signature of *Pax5* ^Jak2/+^ B‐ALL cells

To investigate the oncogenic function of Pax5‐Jak2 in the *Pax5* ^Jak2/+^ B‐ALL model, we next performed RNA‐seq analysis with tumors isolated from the lymph nodes of moribund *Pax5* ^Jak2/+^ mice. As reference tumors, we analyzed B‐ALLs that developed in the lymph nodes of *Pax5* ^+/−^ *Cdkn2ab* ^+/−^ mice, a tumor model that lacks constitutively activated JAK‐STAT signaling (Smeenk *et al*, 2017). The *Pax5* ^Jak2/+^ and control *Pax5* ^+/−^ *Cdkn2ab* ^+/−^ B‐ALL tumors were assigned by principal component analysis (PCA) between pro‐B and large pre‐B cells in early B cell development (Appendix Fig S2E) and yet differed from each other in expression (Appendix Fig S2F). Moreover, the *Pax5* ^Jak2/+^ B‐ALL cells were of oligoclonal origin, as they predominantly expressed a few V_H_ genes of the immunoglobulin heavy‐chain locus (Appendix Fig S2G). By defining differentially regulated genes by an expression difference of > 3‐fold and the aforementioned criteria, we identified 254 upregulated and 144 downregulated genes in *Pax5* ^Jak2/+^ B‐ALLs relative to the control B‐ALLs (Fig 2C and Dataset EV3). Unexpectedly, gene set enrichment analyses (GSEA) revealed that 76 repressed Pax5 target genes, identified in pro‐B cells (Appendix Fig S2A and Dataset EV2), were significantly enriched as upregulated genes in *Pax5* ^Jak2/+^ B‐ALL tumors (Fig 2D), which is also exemplified by the expression of 5 representative genes in the respective pro‐B and B‐ALL cells (Fig 2E). Likewise, 39 activated Pax5 target genes were significantly enriched as downregulated genes in *Pax5* ^Jak2/+^ B‐ALL tumors (Fig 2F and G). Hence, 29% of the differentially expressed genes in *Pax5* ^Jak2/+^ B‐ALL cells correspond to regulated Pax5 target genes. These observations strongly suggest that the function of wild‐type Pax5 may also be compromised in *Pax5* ^Jak2/+^ B‐ALL tumors. While the *Ptprc* (CD45) gene was similarly expressed in the *Pax5* ^Jak2/+^ and control B‐ALL tumors (Appendix Fig S2H), the increased expression of the repressed Pax5 target gene *Hnrnpll* (Fig 2E) may explain the decreased B220 expression on *Pax5* ^Jak2/+^ B‐ALL cells, as the RNA‐binding protein hnRNPLL regulates the alternative splicing of exons 4–6 of the *Ptprc* (CD45) mRNA (Oberdoerffer *et al*, 2008) (Fig 2H and Appendix Fig S2I). Low expression of hnRNPLL, as observed upon Pax5‐mediated repression in control B‐ALL and B cells, leads to the inclusion of all three exons in the *Ptprc* mRNA giving rise to the CD45 isoform RABC (known as B220), while increased expression of hnRNPLL, as detected in *Pax5* ^Jak2/+^ B‐ALL cells, results in skipping of individual exons (Fig 2H), giving rise to other CD45 isoforms (Oberdoerffer *et al*, 2008), including RBC and RB (Appendix Fig S2I). Hence, these expression data provided a molecular explanation why B220 downregulation can be used as a surrogate marker for *Pax5* ^Jak2/+^ B‐ALL tumors, and furthermore indicated that the function of wild‐type Pax5 may be impaired in *Pax5* ^Jak2/+^ B‐ALL tumors.

### Loss of wild‐type *Pax5* in B‐ALL by uniparental disomy of the *Pax5* ^Jak2^ allele

**Figure 3:**
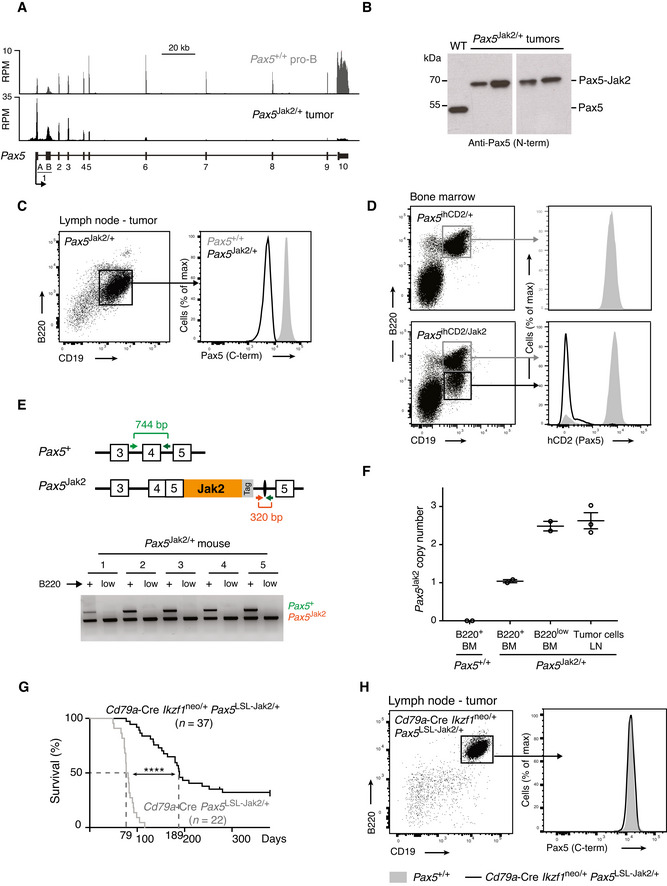
The loss of wild‐type *Pax5* accelerates B‐ALL development in *Pax5* ^Jak2/+^ mice ARNA‐seq expression profile at the *Pax5* locus in *Pax5* ^Jak2/+^ B‐ALL cells (black) and *Pax5* ^+/+^ pro‐B cells (grey). The exon‐intron structure and the two alternative promoters at exons 1A and 1B of *Pax5* are shown below.BImmunoblot analysis of nuclear extracts isolated from *Pax5* ^+/+^ (WT) pro‐B cells and *Pax5* ^Jak2/+^ lymph node tumor cells. The wild‐type Pax5 and Pax5‐Jak2 proteins were detected with an antibody raised again the N‐terminal (N‐term) paired domain of Pax5. The size of marker proteins is indicated in kilodaltons (kDa) to the left.CFlow‐cytometric analysis of Pax5 protein expression by intracellular Pax5 staining of lymph node B220^+^CD19^+^ B cells from a *Pax5* ^+/+^ mouse (grey filled) and B220^low^CD19^+^ B‐ALL cells from a 10‐week‐old *Pax5* ^Jak2/+^ tumor mouse (black). The full‐length Pax5 protein was detected with an antibody that recognizes C‐terminal Pax5 sequences that are absent in the Pax5‐Jak2 protein.DFlow‐cytometric analysis of bone marrow cells from 4‐week‐old *Pax5* ^ihCd2/+^ and *Pax5* ^ihCd2/Jak2^ mice. The expression of human (h) CD2 from the *Pax5* ^ihCd2^ allele is shown for CD19^+^B220^+^ (grey) and CD19^+^B220^low^ (black) B cells.ELoss of the wild‐type *Pax5* allele in CD19^+^B220^low^ B cells from the bone marrow of 6‐week‐old *Pax5* ^Jak2/+^ mice. Genomic DNA isolated from flow cytometry‐sorted CD19^+^B220^+^ (+) and CD19^+^B220^low^ (low) B cells was analyzed by PCR with primers amplifying exon 4 of the wild‐type Pax5 allele and unique sequences of the *Pax5* ^Jak2^ allele (Table EV1), as shown in the schematic diagram of the two *Pax5* alleles. The positions of the PCR fragments corresponding to the two *Pax5* alleles are indicated to the right of the agarose gel.FCopy number analysis of the *Pax5* ^Jak2^ allele by quantitative PCR analysis with primers amplifying unique sequences across the *Pax5* exon 5‐*Jak2* exon 19 junction of the *Pax5* ^Jak2^ allele. The PCR analysis was performed with genomic DNA isolated from lymph node (LN) *Pax5* ^Jak2/+^ B‐ALL cells (*n* = 3 mice), from flow cytometry‐sorted CD19^+^B220^+^ and CD19^+^B220^low^ B cells from the bone marrow (BM) of 6‐week‐old *Pax5* ^Jak2/+^ mice (*n* = 2 each) and from CD19^+^B220^+^ B cells from *Pax5* ^+/+^ bone marrow (*n* = 2). The PCR data were normalized to the product amplified from a control region located 35 Mb upstream of *Pax5*, and the ratio obtained for B220^+^ B cells from *Pax5* ^Jak2/+^ bone marrow was set to 1 copy number of the *Pax5* ^Jak2^ allele.GKaplan–Meier survival analysis of *Cd79a*‐Cre *Ikzf1* ^neo/+^ *Pax5* ^LSL‐Jak2/+^ (black) and *Cd79a*‐Cre *Pax5* ^LSL‐Jak2/+^ (grey) mice. Statistical analysis of the survival curves was performed with the log‐rank (Mantel‐Cox) test; ****P < 0.0001. *n*, number of mice analyzed.HFlow‐cytometric analysis of B220 and CD19 expression in B‐ALL tumor cells from the lymph node of a *Cd79a*‐Cre *Ikzf1* ^neo/+^ *Pax5* ^LSL‐Jak2/+^ mouse (black; left). Pax5 expression in these B‐ALL tumor cells (black line) and control *Pax5* ^+/+^ lymph node B cells (grey filled) was determined by intracellular Pax5 staining (right). Source data are available online for this figure.

To study why the function of Pax5 may be impaired in *Pax5* ^Jak2/+^ B‐ALL cells, we next compared the RNA‐seq expression pattern at the *Pax5* locus in *Pax5* ^+/+^ pro‐B cells and *Pax5* ^Jak2/+^ B‐ALL tumors. Whereas all 10 *Pax5* exons were expressed in *Pax5* ^+/+^ pro‐B cells, abundant expression was detected in *Pax5* ^Jak2/+^ B‐ALL cells only from exon 1 to exon 5, which code for the N‐terminal paired domain present in the Pax5‐Jak2 fusion protein (Fig 3A). Consistent with the absence of wild‐type *Pax5* mRNA, *Pax5* ^Jak2/+^ B‐ALL cells failed to express full‐length Pax5 protein, in contrast to the Pax5‐Jak2 protein, as shown by immunoblot analysis with a Pax5 paired domain‐specific antibody (Fig 3B). Absence of the wild‐type Pax5 protein was confirmed by intracellular Pax5 staining of *Pax5* ^Jak2/+^ B‐ALL cells with a C‐terminal Pax5‐specific antibody that is unable to detect the Pax5‐Jak2 protein (Fig 3C).

To investigate the developmental onset of the *Pax5* expression loss, we took advantage of the *Pax5* ^ihCd2^ allele, which carries an IRES‐*hCd2* gene in the 3’ untranslated region of *Pax5* and thus reports Pax5 expression by giving rise to the expression of human (h) CD2 (Fuxa & Busslinger, 2007). To this end, we generated *Pax5* ^Jak2/ihCd2^ mice and analyzed the bone marrow of these mice at the age of 4 weeks by flow cytometry, which revealed that hCD2 was expressed by B220^+^ cells but was already lost in all B220^low^ cells (Fig 3D). These data further confirmed that the downregulation of B220 expression is an ideal surrogate marker for monitoring the loss of wild‐type Pax5 expression in *Pax5* ^Jak2/+^ B‐ALL cells.

We next examined whether the wild‐type *Pax5* allele was lost in leukemic *Pax5* ^Jak2/+^ B cells. For this, we sorted B220^+^ and B220^low^ B cells from the bone marrow of 6‐week‐old *Pax5* ^Jak2/+^ mice and analyzed genomic DNA of these cells by PCR with specific primers that amplified exon 4 of the wild‐type *Pax5* allele or unique sequences of the *Pax5* ^Jak2^ allele, respectively (Fig 3E). While the *Pax5* ^Jak2^ allele was identified in both B220^+^ and B220^low^ B cells, the *Pax5* exon 4 could only be amplified from the B220^+^ B cells in all *Pax5* ^Jak2/+^ mice analyzed (Fig 3E). Moreover, copy number alteration in the *Pax5*‐containing genomic region could not be observed by whole genome sequencing at a 10‐fold coverage. As wild‐type *Pax5* sequences are present on both sides of the *Jak2* cDNA insertion in the *Pax5* ^Jak2^ allele (Fig 1A), it is conceivable that the *Jak2* insertion may be copied by interchromosomal homologous recombination into the wild‐type *Pax5* allele by a process known as acquired uniparental disomy or copy‐neutral loss of heterozygosity (Tuna *et al*, 2009). To investigate a possible copy‐neutral gain of the *Pax5* ^Jak2^ allele, we performed quantitative PCR analyses with primers that amplified the unique sequences at the *Pax5* exon 5–*Jak2* exon 19 junction of the *Pax5* ^Jak2^ allele (Fig 3F). To allow for normalization of the PCR data, we amplified a control region in intron 2 of the *Car8* gene, located 35 Mb upstream of *Pax5*, and then set the ratio obtained with B220^+^ B cells from *Pax5* ^Jak2/+^ bone marrow to 1 copy number for the *Pax5* ^Jak2^ allele (Fig 3F). This analysis revealed the gain of a second *Pax5* ^Jak2^ allele in B220^low^ B cells and B‐ALL cells of *Pax5* ^Jak2/+^ mice (Fig 3F). Conversely, the loss of the wild‐type *Pax5* allele in B220^low^ B cells and B‐ALL cells was confirmed by quantitative PCR analyses with primers amplifying a 150‐bp sequence in *Pax5* intron 3 that was absent on the *Pax5* ^Jak2^ allele (Appendix Fig S3A). Together, these data revealed a strong selection pressure to lose the wild‐type *Pax5* allele and to gain a second *Pax5* ^Jak2^ allele by acquired uniparental disomy in leukemic B220^low^ B cells of the *Pax5* ^Jak2/+^ mouse model.

### Pax5 loss is not essential for B‐ALL formation, but accelerates tumor progression

To investigate whether the loss of Pax5 is a prerequisite for leukemia formation, we reasoned that ectopic transcription of *Pax5* from a heterologous locus may maintain Pax5 expression in B cells of *Pax5* ^Jak2/+^ mice. For this, we took advantage of the *Ikzf1* ^neo^ allele, which contains a *lox*P‐flanked neomycin (neo) resistance gene upstream of a *Pax5* mini‐gene in the *Ikaros* (*Ikzf1*) locus (Souabni *et al*, 2002). Cre‐mediated deletion of the neo cassette results in Pax5 expression from the *Ikzf1* ^Pax5^ allele (Souabni *et al*, 2002). As ectopic Pax5 expression in the T‐lymphoid lineage leads to the development of an aggressive T cell lymphoma (Souabni *et al*, 2007), we used the *Cd79a*‐Cre line (Hobeika *et al*, 2006) to convert the *Ikzf1* ^neo^ to the *Ikzf1* ^Pax5^ allele only at the onset of B cell development. Hence, we generated *Cd79a*‐Cre *Ikzf1* ^neo/+^ *Pax5* ^LSL‐Jak2/+^ and control *Cd79a*‐Cre *Pax5* ^LSL‐Jak2/+^ mice, which we monitored for the development of B cell leukemia. Kaplan–Meier survival analysis revealed that the control *Cd79a*‐Cre *Pax5* ^LSL‐Jak2/+^ mice had a median survival of 79 days (Fig 3G) and thus died as rapidly as *Pax5* ^Jak2/+^ mice (Fig 1F). *Cd79a*‐Cre *Ikzf1* ^neo/+^ *Pax5* ^LSL‐Jak2/+^ mice also developed B‐ALL, although with a longer latency and incomplete penetrance, as 27% of these mice were still alive after one year (Fig 3G). B‐ALL tumors from the lymph nodes of *Cd79a*‐Cre *Ikzf1* ^neo/+^ *Pax5* ^LSL‐Jak2/+^ mice had a similar cell surface phenotype as *Pax5* ^Jak2/+^ B‐ALL tumors except for higher expression of B220 and CD19 (Fig 3H), although they still lost the wild‐type *Pax5* allele (Appendix Fig S3B). Consistent with normal B220 expression, intracellular Pax5 staining revealed that the B‐ALL tumors of *Cd79a*‐Cre *Ikzf1* ^neo/+^ *Pax5* ^LSL‐Jak2/+^ mice expressed high Pax5 levels similar to the wild‐type B cells (Fig 3H). In summary, we conclude that the loss of Pax5 expression was not strictly required for leukemia formation, although it clearly accelerated tumor development.

To determine whether the wild‐type *PAX5* allele is present or absent in human PAX5‐JAK2^+^ B‐ALL cells, we interrogated the RNA‐seq data of 8 human PAX5‐JAK2^+^ B‐ALL tumors to identify sequence reads spanning the unique exon junctions of the wild‐type *PAX5* gene (exon 5‐exon 6) and *PAX5‐JAK2* rearrangement (*PAX5* exon 5‐*JAK2* exon 19). Sequence reads could be detected at both unique exon junctions in all human PAX5‐JAK2^+^ B‐ALLs, in contrast to the absence of sequence reads at the *Pax5* exon 5‐exon 6 junction in the murine *Pax5* ^Jak2/+^ tumors (Appendix Fig S3C, left). Interestingly, the PAX5‐JAK2 transcripts were increased in 6 of the 8 B‐ALLs, resulting in an average percentage of 68.4% for *PAX5‐JAK2* mRNA compared with 31.6% for full‐length *PAX5* mRNA (Appendix Fig S3C, right), whereas a similar analysis of PAX5‐ETV6^+^ B‐ALLs revealed a 1:1 ratio of both *PAX5* transcripts (Smeenk *et al*, 2017). Analysis of the RNA‐seq expression pattern at the human *PAX5* locus corroborated that all 10 *PAX5* exons were expressed in PAX5‐JAK2^+^ B‐ALLs (Appendix Fig S3D), contrary to the situation observed in murine *Pax5* ^Jak2/+^ B‐ALLs (Fig 3A). These data therefore demonstrate that the wild‐type *PAX5* allele is not lost in human PAX5‐JAK2^+^ B‐ALLs. The discrepancy between the human *PAX5‐JAK2* rearrangement and the murine *Pax5* ^Jak2/+^ model is likely caused by the insertion of the *Jak2* cDNA sequence in the mouse *Pax5* locus, which provides an ideal substrate for acquired uniparental disomy due to the presence of *Pax5* sequence homologies on both sides of the *Jak2* cDNA insertion.

### The Jak2 kinase activity is required for the development and maintenance of *Pax5* ^Jak2/+^ B‐ALL

**Figure 4:**
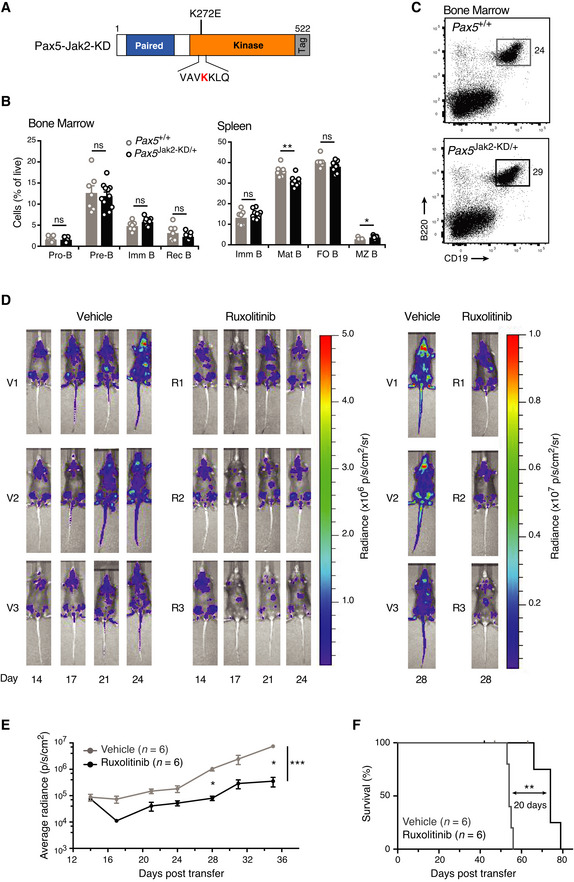
The development of *Pax5* ^Jak2/+^ tumors depends on the Jak2 kinase activity ASchematic diagram of the kinase‐dead (KD) Pax5‐Jak2 fusion protein, which is encoded by the *Pax5* ^Jak2‐KD^ allele and contains the K272E mutation known to abolish the Jak2 kinase function.B, CFlow‐cytometric analysis of bone marrow and spleen from *Pax5* ^Jak2‐KD/+^ (black; *n* = 11) and control *Pax5* ^+/+^ (grey; *n* = 7) mice at the age of 6–8 weeks. The frequencies of the indicated B cell types are shown for each organ (B). The data are presented as mean percentages with SEM and were statistically analyzed by multiple *t*‐tests (unpaired and two‐tailed with Holm‐Šídák's correction); ns (*P* > 0.5), *P < 0.05, **P < 0.01. See Methods for flow‐cytometric definition of the different cell types. The expression of CD19 and B220 on bone marrow B cells of the indicated genotypes is shown in (C).D, ETumor progression in mice, transplanted with *Pax5* ^Jak2‐Luc/+^ tumor cells, in the presence or absence of the JAK1/2 inhibitor ruxolitinib. At day 14 after cell transfer, the transplanted mice were treated twice daily with ruxolitinib or vehicle, and the tumor mass was monitored by bioluminescence measurements. Images of three representative mice with or without ruxolitinib treatment are shown in (D). The bioluminescence measurements of transplanted mice treated with ruxolitinib (black) or vehicle (grey) at the indicated days after cell transfer are shown as mean radiance values with SEM (E). *n*, number of mice analyzed. Statistical data are shown as mean value with SEM and were analyzed by the mixed‐effect model REML with Geisser–Greenhouse’s correction and Tukey’s multiple comparison test: *P < 0.05, ***P < 0.001.FKaplan–Meier survival analysis of the transplanted mice evaluated in (D, E) upon prolonged treatment with ruxolitinib (black) and vehicle (grey). Statistical analysis of the survival curves (F) was performed with the log‐rank (Mantel‐Cox) test; **P < 0.01.

We next investigated whether the kinase activity of Pax5‐Jak2 is essential for leukemia development. As mutation of the full‐length JAK2 protein at lysine (K) 882 to glutamic acid (E) in the ATP‐binding loop was previously shown to abolish its kinase activity (Feng *et al*, 1997), we introduced the equivalent K272E mutation in the *Pax5* ^Jak2^ allele to generate a kinase‐dead (KD) Pax5‐Jak2 protein (Fig 4A and Appendix Fig S4A). The Pax5‐Jak2‐KD protein was expressed in pro‐B cells of *Pax5* ^Jak2‐KD/+^ mice, albeit at a 4‐fold lower level relative to the wild‐type Pax5 protein, as shown by immunoblot analysis of nuclear pro‐B cell extracts (Appendix Fig S4B). All B cell subsets were present at normal frequencies and did not downregulate B220 expression in the bone marrow and spleen of *Pax5* ^Jak2‐KD/+^ mice at the age of 2 months (Fig 4B and C). Notably, no mice succumbed to leukemia during the observation period of 12 months (Appendix Fig S4C). While the 4‐fold lower expression of the Pax5‐Jak2‐KD protein in pro‐B cells is expected to significantly delay the tumor onset, the complete absence of B‐ALL in 1‐year‐old *Pax5* ^Jak2‐KD/+^ mice nevertheless indicates a critical role of the Pax5‐Jak2 kinase activity in the initiation of leukemia development.

To determine whether the kinase activity is required for tumor maintenance, we investigated the *in vivo* sensitivity of *Pax5* ^Jak2/+^ B‐ALLs to JAK2 inhibition. Previous studies showed that *in vitro* cultured cell lines ectopically expressing PAX5‐JAK2 rapidly lose cell viability upon treatment with the JAK1/2 inhibitor ruxolitinib (Roberts *et al*, 2014; Schinnerl *et al*, 2015; Hurtz *et al*, 2020). We next studied the effect of ruxolitinib on the maintenance of the murine *Pax5* ^Jak2/+^ B‐ALL tumors *in vivo*. As wild‐type pro‐B and pre‐B cells abundantly express Jak1 but not Jak2 (ImmGen database), ruxolitinib may mediate its effect by inhibition of the endogenous Jak1, transgenic Pax5‐Jak2, or both kinases in *Pax5* ^Jak2/+^ B‐ALL tumors. To perform the ruxolitinib inhibition experiment, we took advantage of the *Pax5* ^Jak2‐Luc/+^ mice (Appendix Fig S1B), which additionally expressed luciferase in B cells, thus facilitating tumor monitoring by *in vivo* bioluminescence measurement. The pro‐B cells of *Pax5* ^Jak2‐Luc/+^ mice expressed 2‐fold lower levels of Pax5‐Jak2 protein compared with *Pax5* ^Jak2/+^ pro‐B cells (Appendix Fig S1E and F). Consequently, the *Pax5* ^Jak2‐Luc/+^ mice develop B‐ALL with a 3‐fold longer latency of 214 days (Appendix Fig S4D and E) relative to the *Pax5* ^Jak2/+^ mice (74 days; Fig 1F). Tumor cells from lymph nodes of moribund *Pax5* ^Jak2‐Luc/+^ mice (Ly5.2^+^) were harvested, sorted as B220^low^ cells by flow cytometry, and 10^5^ cells were transferred into sublethally irradiated Ly5.1^+^ C57BL/6 recipient mice. Tumor cell engraftment was verified by *in vivo* bioluminescence measurement 14 days post‐transfer, followed by twice‐daily treatment with either ruxolitinib or vehicle (Appendix Fig S4F). Bioluminescence monitoring revealed that ruxolitinib treatment significantly slowed down leukemia progression in two independent experiments (Fig 4D and E, and Appendix Fig S4G). Consequently, ruxolitinib treatment prolonged the survival of the transplanted mice (with a median survival of 74 days) by 20 days compared with vehicle‐treated mice (with a median survival of 54 days; Fig 4F). In summary, we conclude that the kinase activity of Pax5‐Jak2 is important for both the development and maintenance of the *Pax5* ^Jak2/+^ B‐ALL tumors.

### Inefficient competition of Pax5‐Jak2 for DNA binding in the presence of wild‐type Pax5

**Figure 5:**
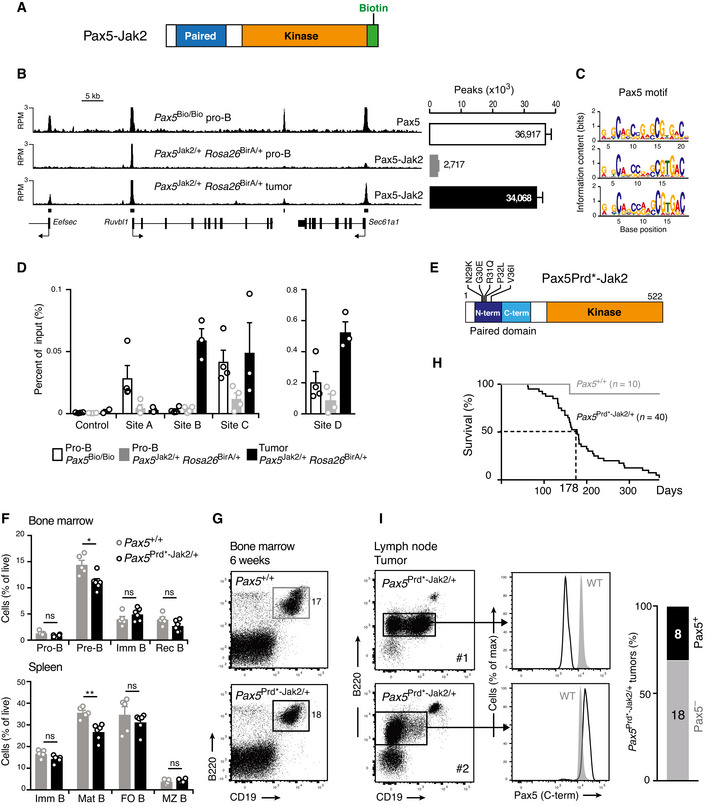
The DNA‐binding function of Pax5‐Jak2 is important for leukemia formation AA schematic diagram of the Pax5‐Jak2 protein containing a C‐terminal biotin acceptor sequence that can be biotinylated *in vivo* by the *E. coli* biotin ligase BirA upon expression from the *Rosa26* ^BirA^ allele (Driegen *et al*, 2005).BGenome‐wide binding of Pax5‐Jak2 in *in vitro* cultured pro‐B cells from *Pax5* ^Jak2/+^ *Rosa26* ^BirA/+^ mice at the age of 3 weeks (expressing Pax5) and *ex vivo Pax5* ^Jak2/+^ *Rosa26* ^BirA/+^ B‐ALL tumors (lacking Pax5), as determined by Bio‐ChIP‐seq analysis (Revilla‐i‐Domingo *et al*, 2012). The DNA‐binding pattern of full‐length Pax5 was determined by Bio‐ChIP‐seq analysis of *ex vivo* sorted *Pax5* ^Bio/Bio^ pro‐B cells, which carried a C‐terminal biotin acceptor sequence together with an IRES*‐*BirA gene insertion in the 3' untranslated region of *Pax5* (McManus *et al*, 2011). Two independent Bio‐ChIP‐seq experiments were performed for each cell type. Representative binding patterns of Pax5 and Pax5‐Jak2 in the three B cell types are shown for a selected genomic region, with horizontal bars indicating Pax5 or Pax5‐Jak2 peaks that were identified by MACS peak calling (left). The number of Pax5 (white) and Pax5‐Jak2 (grey or black) peaks, which were defined by stringent MACS peak calling with a *P* value of < 10^−10^ in the three B cell types, are shown to the right.CPax5 recognition motifs identified in the three different B cell types by *de novo* motif discovery analysis of the top 300 peaks (see Appendix Supplementary Methods).DBio‐ChIP‐qPCR analysis of Pax5 binding at four sites, which were selected for their differential Pax5 binding in the three cell types, as determined by Bio‐ChIP‐seq analysis (Appendix Fig S5A). The amount of precipitated DNA was determined as percentage relative to input and is shown as mean value with SEM based on 3–4 independent biological experiments. A gene‐less region on Chr.1 was used as a negative control.ESchematic diagram of the mutant Pax5‐Jak2 protein carrying the indicated amino acid changes that were introduced in the N‐terminal part of the paired domain by CRISPR/Cas9‐mediated mutagenesis in the *Pax5* ^Prd*‐Jak2^ allele (Appendix Fig S5E).F, GFlow‐cytometric analysis of the bone marrow and spleen from *Pax5* ^Prd*‐Jak2 /+^ (black; *n* = 6) and control *Pax5* ^+/+^ (grey; *n* = 5) mice at the age of 6–7 weeks. The frequency of the indicated B cell types in each organ is indicated in (F), and the flow‐cytometric analysis of CD19 and B220 expression on bone marrow B cells is shown in (G). Statistical data are shown as mean percentages with SEM and were analyzed by multiple *t*‐tests (unpaired and two‐tailed with Holm‐Šídák's correction): ns > 0.05; *P < 0.05; **P < 0.01. Each dot corresponds to one mouse. See Methods section for flow‐cytometric definition of the different cell types.HKaplan‐Meier survival analysis of *Pax5* ^Prd*‐Jak2 /+^ (black) and *Pax5* ^+/+^ (grey) mice. A *P* value of < 0.0001 was determined for the survival curves by statistical analysis with the log‐rank (Mantel‐Cox) test. *n*, number of mice analyzed.IFlow‐cytometric analysis and intracellular Pax5 staining of lymph node tumor cells from two moribund *Pax5* ^Prd*‐Jak2 /+^ mice (black line), which differed by the absence (mouse #1) or presence (mouse #2) of Pax5 expression in the tumor cells. *Pax5* ^+/+^ (WT) B cells (filled grey) were used as controls. The apparently higher Pax5 expression level in the blasting tumor cells of mouse # 2 compared with that of quiescent *Pax5* ^+/+^ B cells may reflect a difference in cell size rather than in Pax5 expression. The percentage and number of Pax5^+^ and Pax5^−^ *Pax5* ^Prd*‐Jak2 /+^ tumors analyzed is shown to the right.

The consistent loss of the wild‐type *Pax5* allele in *Pax5* ^Jak2/+^ tumor cells raised the question of whether the full‐length Pax5 protein may interfere with the function of Pax5‐Jak2. Since the N‐terminal DNA‐binding paired domain is the only common region between Pax5‐Jak2 and full‐length Pax5 (Appendix Fig S1A), it is conceivable that both proteins may compete for DNA binding in *Pax5* ^Jak2/+^ B cells. To test this hypothesis, we analyzed the genome‐wide binding pattern of both Pax5 proteins. In order to distinguish the two proteins, we added a biotin acceptor sequence in frame to the C‐terminus of the Pax5‐Jak2 protein encoded by the *Pax5* ^Jak2^ allele (Fig 5A and Appendix Fig S1B), which allowed Pax5‐Jak2 to be specifically biotinylated *in vivo* by the *E*. *coli* biotin ligase BirA expressed from the *Rosa26* ^BirA^ allele (de Boer *et al*, 2003; Driegen *et al*, 2005). We thus generated *Pax5* ^Jak2/+^ *Rosa26* ^BirA/+^ mice and used *in vitro* cultured pro‐B cells from young mice (expressing Pax5) or B‐ALL tumors (lacking Pax5) to determine the genome‐wide DNA‐binding pattern of Pax5‐Jak2 by streptavidin‐mediated chromatin precipitation coupled with deep sequencing (Bio‐ChIP‐seq; Revilla‐i‐Domingo *et al*, 2012). For comparison, we also determined the DNA‐binding profile of full‐length Pax5 by Bio‐ChIP‐seq analysis of *Pax5* ^Bio/Bio^ pro‐B cells, which carried a C‐terminal biotin acceptor sequence together with an IRES*‐*BirA gene insertion in the 3' untranslated region of *Pax5* (McManus *et al*, 2011).

Peak calling with a stringent *P* value of < 10^‐10^ identified 36,917 Pax5 peaks in *Pax5* ^Bio/Bio^ pro‐B cells, 2,717 Pax5‐Jak2 peaks in *Pax5* ^Jak2/+^ *Rosa26* ^BirA/+^ pro‐B cells, and 34,068 Pax5‐Jak2 peaks in *Pax5* ^Jak2/+^ *Rosa26* ^BirA/+^ B‐ALL cells (Fig 5B). Analysis of the 300 top‐ranked peaks with the *de novo* motif‐discovery program MEME‐ChIP (Machanick & Bailey, 2011) identified the Pax5 motif in both Pax5‐Jak2 and Pax5 peaks (Fig 5C), consistent with the two proteins sharing the same DNA‐binding domain. Four sites, which exhibited differential binding of Pax5 and Pax5‐Jak2 in the Bio‐ChIP‐seq data (Appendix Fig S5A), were selected for validation by Bio‐ChIP‐qPCR analysis of independently prepared *Pax5* ^Bio/Bio^ pro‐B cell, *Pax5* ^Jak2/+^ *Rosa26* ^BirA/+^ pro‐B cell, and *Pax5* ^Jak2/+^ *Rosa26* ^BirA/+^ B‐ALL samples (Appendix Fig S5B). The Bio‐ChIP‐seq and Bio‐ChIP‐qPCR methods both detected the same binding pattern at each selected site in the three different cell types (Fig 5D and Appendix Fig S5A). Notably, the Bio‐ChIP‐seq analysis identified a 13.6‐fold lower number of Pax5‐Jak2 peaks in *Pax5* ^Jak2/+^ *Rosa26* ^BirA/+^ pro‐B cells compared with the Pax5 peaks in *Pax5* ^Bio/Bio^ pro‐B cells (Fig 5B), which unequivocally demonstrated that the Pax5‐Jak2 protein was unable to efficiently compete for DNA binding in the presence of the full‐length Pax5 protein. Conversely, the loss of Pax5 in *Pax5* ^Jak2/+^ *Rosa26* ^BirA/+^ B‐ALL cells resulted in a similarly high number and extensive overlap of the Pax5‐Jak2 peaks compared with the Pax5 peaks identified in *Pax5* ^Bio/Bio^ pro‐B cells, although the binding density was still higher for full‐length Pax5 compared with Pax5‐Jak2 (Fig 5B and Appendix Fig S5C and D). We therefore conclude that the consistent loss of Pax5 in *Pax5* ^Jak2/+^ B‐ALL tumors allows Pax5‐Jak2 to bind to its target sites in the genome, which strongly argues for a nuclear function of Pax5‐Jak2.

### The DNA‐binding function of Pax5‐Jak2 contributes to leukemia formation

We next investigated whether the DNA‐binding activity of Pax5‐Jak2 is essential for leukemia formation. To this end, we mutated the N‐terminal region of the paired domain by introducing five amino acid changes (N29K, G30S, R31Q, P32L, V36I) by CRISPR/Cas9‐mediated mutagenesis in the *Pax5* ^Prd^*^‐Jak2^ allele (Fig 5E and Appendix Fig S5E). The selected five amino acid residues are known to bind to the DNA backbone in the minor groove of the Pax5‐binding sequence (Garvie *et al*, 2001) and are thus predicted to affect DNA‐binding of the N‐terminal paired domain region, consistent with the fact that the amino acids N29, R31, and P32 were shown to be mutated in human B‐ALLs (Gu *et al*, 2019). As shown by immunoblot analysis, the Pax5Prd*‐Jak2 protein was expressed at a lower level relative to the Pax5 protein in *Pax5* ^Prd^*^‐Jak2/+^ B cells (Appendix Fig S5F), which may be caused by an interference of the five mutant amino acid residues with efficient recognition of the Pax5Prd*‐Jak2 protein by the anti‐Pax5 paired domain antibody. We therefore analyzed the expression of the *Pax5* ^Prd^*^‐Jak2^ allele by RT‐qPCR analysis of *Pax5* ^Prd^*^‐Jak2/+^ pro‐B cells, which indicated that the Pax5Prd*‐Jak2 mRNA was expressed at a similar level as *Pax5‐Jak2* and *Pax5‐Jak2‐Luc* mRNA in *Pax5* ^Jak2/+^ and *Pax5* ^Jak2‐Luc/+^ pro‐B cells (Appendix Fig S5G).

B cell development in the bone marrow and spleen of 6–7‐week‐old *Pax5* ^Prd^*^‐Jak2/+^ mice was largely normal except for a modest decrease in pre‐B and mature B cells (Fig 5F). Importantly, leukemic B220^low^ cells could not be detected in the bone marrow of 6–7‐week‐old *Pax5* ^Prd^*^‐Jak2/+^ mice (Fig 5G). However, the *Pax5* ^Prd^*^‐Jak2/+^ mice still developed B‐ALL with a median survival of 178 days (Fig 5H) and thus lived on average 100 days longer than *Pax5* ^Jak2/+^ mice (Fig 1F). The *Pax5* ^Prd^*^‐Jak2/+^ tumor cells also downregulated B220 and CD19 expression (Fig 5I) and had a similar cell surface phenotype as the *Pax5* ^Jak2/+^ tumor cells (Fig 5I). Interestingly, 31% of the tumors did not lose wild‐type Pax5 expression, as revealed by intracellular Pax5 staining (Fig 5I). However, stratification of the *Pax5* ^Prd^*^‐Jak2/+^ tumors according to their Pax5 expression status did not reveal any survival advantage for mice with tumors that still expressed the wild‐type Pax5 protein (Appendix Fig S5H). The delayed development of *Pax5* ^Prd^*^‐Jak2/+^ B‐ALLs could be caused by an altered DNA‐binding potential of the mutant paired domain. To test this hypothesis, we used *in vitro* cultured *Pax5* ^Jak2/+^ and *Pax5* ^Prd^*^‐Jak2/+^ B‐ALL cells, which lost the wild‐type *Pax5* allele, for ChIP‐seq analysis with an antibody detecting the N‐terminal paired domain of Pax5. Notably, the mutant Pax5‐Jak2 protein still bound to 20,692 sites in the genome (Appendix Fig S5I). However, the mutant protein uniquely bound to 52% of these sites and furthermore failed to interact with 33% of all Pax5‐Jak2‐binding sites (Appendix Fig S5I), suggesting that the five N‐terminal amino acid substitutions resulted in an altered DNA‐binding specificity of the paired domain. In summary, these data indicate that the DNA‐binding activity of Pax5‐Jak2 contributes to leukemia development.

### Nuclear Pax5‐Jak2 does neither phosphorylate H3Y41 nor induce active chromatin

**Figure 6:**
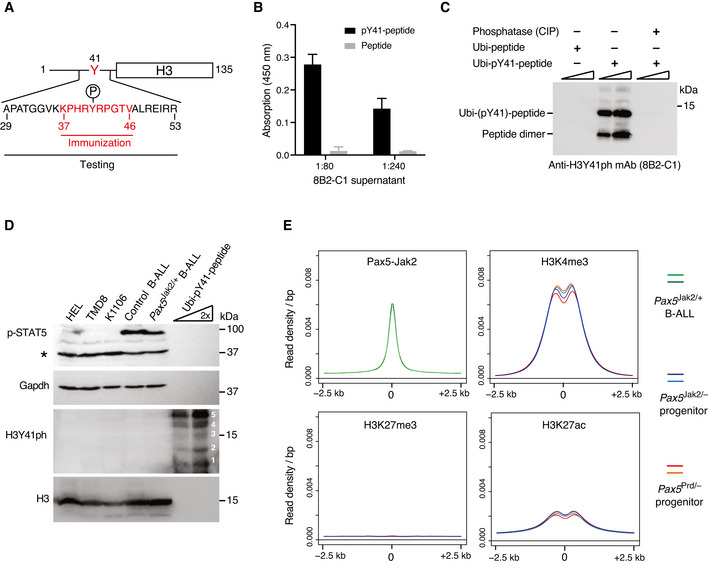
Pax5‐Jak2 does not function as an epigenetic regulator Schematic diagram of histone H3 indicating the position of Y41. The pY41‐peptide (amino acids 37–46, red) was used for immunizing mice and the subsequent generation of the hybridoma cell line 8B2‐C1 (see Methods). The longer phosphorylated (pY41) and nonphosphorylated peptides (amino acids 29–53, black) were used for ELISA and immunoblot analysis.Specific detection of the pY41‐peptide by the antibody produced by the hybridoma cell line 8B2‐C1. The plate‐bound pY41‐peptide, in contrast to the corresponding nonphosphorylated peptide, is specifically detected by antibodies in the serially diluted supernatant of the hybridoma cells by ELISA assays, as measured by absorption at the wavelength of 450 nm. Mean values with SEM are shown for two independent experiments.Specificity of the anti‐H3Y41ph antibody, as shown by immunoblot analysis. One phosphorylated (pY41) or nonphosphorylated peptide was coupled to ubiquitin (Ubi, 8.5 kDa), followed by separation of the protein conjugates on SDS‐PAGE and immunoblot detection with the anti‐H3Y41ph antibody present in the 8B2‐C1 cell supernatant. Following treatment with calf intestinal alkaline phosphatase (CIP), the dephosphorylated Ubi‐pY41 peptide conjugate could no longer be detected with the anti‐H3Y41ph antibody.Immunoblot analysis of whole‐cell extracts prepared from *Pax5* ^Jak2/+^ and control *Pax5* ^Etv6/+^ *Cdkn2ab* ^+/−^ B‐ALL cells as well as from the human HEL, TMD8 and K1106 cell lines. Phosphorylated (p) STAT5 was detected with an anti‐STAT5 (pY694) antibody and the pY41‐peptide with the purified anti H3Y41ph antibody (Appendix Fig S6B). One to five pY41‐peptides were coupled to ubiquitin, which was added in the range of 50 ng per well. The Gapdh and histone H3 proteins were analyzed as loading control. An unspecific protein is denoted by an asterisk. One representative of 5 immunoblot experiments is shown.No evidence for an epigenetic function of Pax5‐Jak2 in early B cell development. The genome‐wide distribution of the histone marks H3K4me3, H3K27ac and H3K27me3 in *in vitro* cultured *Pax5* ^Jak2/−^ and *Pax5* ^Prd/−^ progenitor cells was determined by ChIP‐seq analysis (Appendix Fig S6C). The average density of the three histone marks in these progenitor cells was determined for a region from −2.5 kb to +2.5 kb from the summit of the Pax5‐Jak2 peaks identified in *Pax5* ^Jak2/+^ B‐ALL cells (Fig 5B). The results of two different ChIP‐seq experiments per cell type are shown. The expression of the Pax5‐Jak2 protein and paired domain (Prd) polypeptide in *Pax5* ^Jak2/−^ and *Pax5* ^Prd/−^ progenitor cells is shown in Appendix Fig S2D. Source data are available online for this figure.

To gain further insight into the nuclear function of Pax5‐Jak2, we next studied the subcellular localization of Pax5‐Jak2 by nuclear‐cytoplasmic cell fractionation and immunoblot analysis, which revealed that the Pax5‐Jak2 protein was almost exclusively present in the nucleus of *Pax5* ^Jak2/+^ B‐ALL cells (Appendix Fig S6A). This evidence further supported the concept of an essential nuclear function for Pax5‐Jak2 that involves DNA binding in the absence of a classical transcriptional activity. We therefore investigated whether Pax5‐Jak2 may indirectly control gene expression by phosphorylating histone H3 on tyrosine 41 (H3Y41ph), as it was previously proposed for nuclear JAK2 (Dawson *et al*, 2009). Due to the discontinued commercial availability of the published rabbit polyclonal anti‐H3Y41ph antibody, we generated a new mouse monoclonal anti‐H3Y41ph antibody (clone 8B2‐C1; IgG1/Igκ) that specifically detected an H3 peptide containing the phosphorylated Y41 (pY41) in ELISA assays and identified the pY41‐peptide conjugated to the ubiquitin protein in immunoblot analysis (Fig 6A–C). We next extensively purified the anti‐H3Y41ph antibody from 8B2‐C1 hybridoma cell supernatants by negative and positive selection on peptide columns (Appendix Fig S6B). Immunoblot analysis of whole‐cell extracts prepared from *Pax5* ^Jak2/+^ or control *Pax5* ^Etv6/+^ *Cdkn2ab* ^+/−^ tumor cells revealed that phosphorylated STAT5 could be readily detected in both tumor cell types (Fig 6D). However, the monoclonal anti‐H3Y41ph antibody (8B2‐C1) could not detect H3Y41 phosphorylation in the *Pax5* ^Jak2/+^ tumor cells, although it readily identified the pY41‐peptide conjugated to ubiquitin (Fig 6D). Moreover, the H3Y41ph modification could also not be detected in HEL, TMD8, and K1106 cells (Fig 6D), which were previously shown to contain abundant H3Y41ph levels upon detection with the published polyclonal anti‐H3Y41ph antibody (Dawson *et al*, 2009; Rui *et al*, 2010, 2016). As a control, abundant H3 expression was observed in all tumor cell lines by analysis with an anti‐H3 antibody (Fig 6D). Based on these data, we conclude that Pax5‐Jak2 does not phosphorylate H3Y41 in *Pax5* ^Jak2/+^ B‐ALL cells.

Phosphorylation of H3Y41 was also shown to expel HP1α from nucleosomes, thus inducing active chromatin leading to gene activation (Dawson *et al*, 2009; Rui *et al*, 2010). We next investigated a possible role of Pax5‐Jak2 in chromatin regulation. For this purpose, *Pax5* ^Jak2/+^ B‐ALL cells could, however, not be analyzed due to the absence of a reference cell type that would be blocked at the same developmental stage as the B‐ALL cells. As B lymphopoiesis was arrested at the same uncommitted progenitor stage in *Pax5* ^Jak2/−^ and control *Pax5* ^Prd/−^ mice (Appendix Fig S2C), we performed ChIP‐seq analysis with *in vitro* cultured *Pax5* ^Jak2/−^ and *Pax5* ^Prd/−^ progenitor cells (Appendix Fig S6C) to investigate the genome‐wide distribution of the active histone marks H3K4me3 and H3K27ac as well as the repressive histone modification H3K27me3. We initially focused our bioinformatic analysis on Pax5‐Jak2‐binding regions identified in *Pax5* ^Jak2/+^ B‐ALL cells (Figs 5B and 6E). Heat maps and density profiles did not reveal any obvious difference in the distribution of the active H3K4me3 and H3K27ac marks at the Pax5‐Jak2‐binding sites, while the repressive H3K27me3 modification was absent at these sites (Fig 6E and Appendix Fig S6C). We subsequently used a window‐based approach (Lun & Smyth, 2016) to scan the entire genome for regions with a differential abundance of the three individual histone marks, which also did not identify any significant differences. We therefore conclude that Pax5‐Jak2 does not function as an epigenetic regulator.

### IL‐7 signaling promotes the development of *Pax5* ^Jak2/+^ B‐ALL

**Figure 7:**
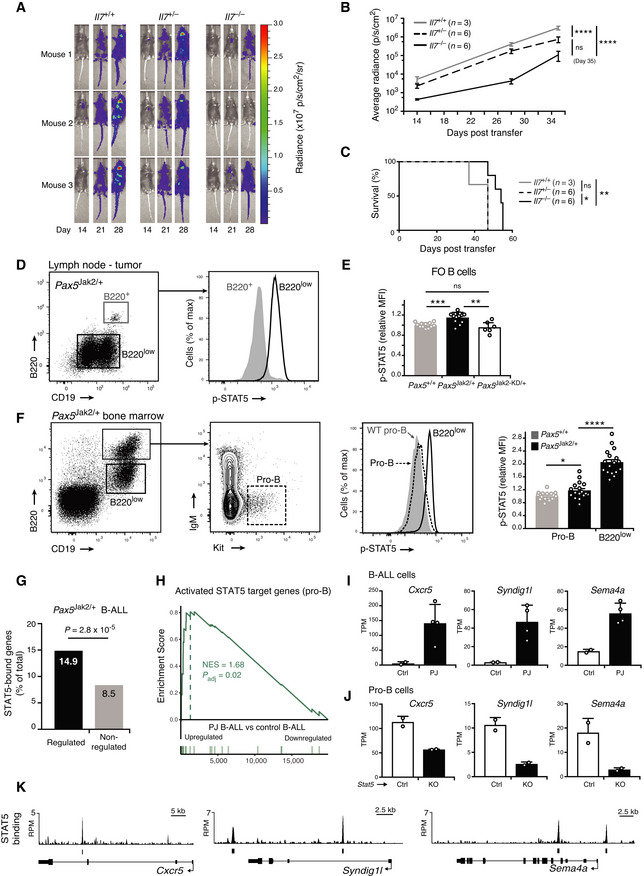
IL‐7 requirement and regulation of STAT5 target genes in *Pax5* ^Jak2/+^ B‐ALLs A, BTumor progression in *Il7* ^+/+^ (grey), *Il7* ^+/−^ (dashed), and *Il7* ^−/−^ (black) mice transplanted with *Pax5* ^Jak2‐Luc/+^ tumor cells, as determined by bioluminescence measurements at the indicated days after cell transfer. Images of three transplanted mice for each *Il7* genotype are shown at the indicated days after cell transfer (A). The bioluminescence measurements of the transplanted mice are shown as mean radiance values with SEM (B) and were analyzed by two‐way ANOVA with Tukey’s multiple comparison test: ns *P* > 0.05, ****P < 0.0001. *n*, number of mice analyzed.CKaplan–Meier survival analysis of *Il7* ^+/+^, *Il7* ^+/−^, and *Il7* ^−/−^ mice transplanted with *Pax5* ^Jak2‐Luc/+^ tumors cells. Statistical analysis of the survival curves was performed with the log‐rank (Mantel‐Cox) test; ns *P* > 0.05, *P < 0.05, **P < 0.01.DPhosphorylation (p) of the STAT5 protein in B220^low^ B‐ALL cells (black) and control nonleukemic B220^+^ B cells (grey) from the lymph node of a 11‐week‐old *Pax5* ^Jak2/+^ mouse. The p‐STAT5 level was determined by intracellular staining with an anti‐STAT5 (pY694) antibody.EQuantification of the median fluorescence intensity (MFI) values of the p‐STAT5 levels determined by intracellular staining of splenic FO B cells from *Pax5* ^+/+^ (grey), *Pax5* ^Jak2/+^ (black), and *Pax5* ^Jak2‐KD/+^ (white) mice at the age of 3–10 weeks (for additional data see Appendix Fig S7F).FFlow cytometric analysis of p‐STAT5 levels in pro‐B cells (B220^+^CD19^+^Kit^+^IgM^−^) and leukemic B220^low^ B cells of *Pax5* ^Jak2/+^ mice (black) as well as in control pro‐B cells of *Pax5* ^+/+^ (WT) mice (grey) at the age of 4–5 weeks. A representative flow cytometric analysis of the bone marrow of a *Pax5* ^Jak2/+^ mouse (left) and representative intracellular p‐STAT5 stainings (middle) are shown. The p‐STAT5 levels in pro‐B and leukemic B220^low^ B cells (right) are quantified as MFI values relative to that of *Pax5* ^+/+^ pro‐B cells. Statistical data (E, F) are presented as mean values with SEM and were analyzed by the two‐tailed unpaired Student’s *t*‐test: *P < 0.05, **P < 0.01, ***P < 0.001, ****P < 0.0001. Each dot corresponds to one mouse.GSTAT5 binding at regulated and nonregulated genes in *Pax5* ^Jak2/+^ B‐ALL cells. The regulated genes corresponded to the 254 up‐ and 144 down‐regulated genes (> 3‐fold) identified in *Pax5* ^Jak2/+^ B‐ALL cells relative to control *Pax5* ^+/−^ *Cdkn2ab* ^+/−^ B‐ALL cells (Fig 2C). The 4,264 nonregulated genes were defined by an expression difference between > –1.25 and < 1.25‐fold. The percentages indicate the overlap of the regulated and nonregulated genes with the 1,606 STAT5‐bound genes, which were determined by ChIP‐seq analysis in wild‐type pro‐B cells. The *P* value was calculated with the Pearson’s chi‐squared test with Yates’ continuity correction.HGSEA analysis of the 19 activated STAT5 target genes identified in pro‐B cells (Appendix Fig S7H), as compared with the ranked log_2_‐fold gene expression changes in *Pax5* ^Jak2/+^ (PJ) B‐ALLs versus control *Pax5* ^+/−^ *Cdkn2ab* ^+/−^ B‐ALLs. NES, normalized enrichment score.IUpregulation of the genes *Cxcr5*, *Syndig1l* and *Sema4a* in *Pax5* ^Jak2/+^ (PJ) B‐ALL cells.J, KIdentification of *Cxcr5*, *Syndig1l* and *Sema4a* as activated STAT5 target genes. Expression of the three genes in pro‐B cells of *Vav‐Bcl2 Rag1*‐Cre *Stat5* ^fl/fl^ (KO) and control (Ctrl) *Vav‐Bcl2 Rag1*‐Cre *Stat5* ^fl/+^ mice (J) was determined by RNA‐seq (Appendix Fig S7H). ChIP‐seq analysis identified STAT5‐binding regions at all three loci (K). Horizontal bars below the ChIP‐seq track indicate STAT5‐binding regions identified by MACS peak calling. RPM, reads per million. Mean TPM values with SEM (I, J) are shown for the following RNA‐seq experiments: 2 (Ctrl B‐ALL), 4 (PJ B‐ALL), 2 (Ctrl pro‐B), and 2 (KO pro‐B).

While establishing *Pax5* ^Jak2/+^ tumor cells in culture, we noticed that IL‐7 strongly stimulated the proliferation of these cells (Appendix Fig S7A), which is consistent with expression of the IL‐7 receptor on these cells (Fig 1I) and with a recent report demonstrating that the IL‐7 sensitivity of leukemic B cells is increased upon Pax5 loss (Ramamoorthy *et al*, 2020). To investigate a possible IL‐7 dependency *in vivo*, we again took advantage of the *Pax5* ^Jak2‐Luc/+^ tumor cell transplant system and injected freshly harvested tumor cells into *Il7* ^+/+^, *Il7* ^+/−^, or *Il7* ^−/−^ recipient mice (Appendix Fig S7B). Weekly bioluminescence analysis revealed that tumor development was delayed in *Il7* ^−/−^ mice compared with control *Il7* ^+/−^ and *Il7* ^+/+^ mice in two independent experiments (Fig 7A and B, and Appendix Fig S7C). Moreover, the survival of tumor‐bearing mice was modestly improved in *Il7* ^−/−^ recipient mice compared with the control *Il7* ^+/−^ and *Il7* ^−/−^ recipient mice (Fig 7C). These results indicate that IL‐7 promotes the growth of *Pax5* ^Jak2/+^ tumor cells also *in vivo*. It therefore appears that IL‐7 signaling and the constitutively active Pax5‐Jak2 kinase cooperatively support the development of *Pax5* ^Jak2/+^ B‐ALL.

### STAT5‐mediated gene regulation in *Pax5* ^Jak2/+^ B‐ALL

As the constitutively active JAK2 kinase domain of the PAX5‐JAK2 protein was shown to phosphorylate STAT5 when ectopically expressed in a JAK2‐deficient human cell line (Schinnerl *et al*, 2015), we next investigated the status of STAT5 phosphorylation in B cells of *Pax5* ^Jak2/+^ mice. Intracellular staining combined with flow‐cytometric analysis revealed a strong increase of phosphorylated STAT5 (p‐STAT5) in B‐ALL cells compared with residual B220^+^ B cells in the lymph nodes of *Pax5* ^Jak2/+^ mice (Fig 7D). Moreover, the p‐STAT5 levels in *Pax5* ^Jak2/+^ B‐ALL cells were reduced at 30 min and lost at 1 h after ruxolitinib treatment (Appendix Fig S7D), while they were reduced at 2 h and lost at 4 h after IL‐7 withdrawal (Appendix Fig S7E), suggesting that STAT5 phosphorylation in these tumor cells depends on both the Pax5‐Jak2 kinase and IL‐7 signaling. To investigate whether the Pax5‐Jak2 kinase could also phosphorylate STAT5 in a B cell type lacking IL‐7R signaling, we analyzed p‐STAT5 levels in follicular (FO) B cells (B220^+^CD19^+^CD21^int^CD23^hi^) from the spleen of 3–5‐week‐old *Pax5* ^Jak2/+^ mice. A small but significant increase of p‐STAT5 was detected in *Pax5* ^Jak2/+^ FO B cells compared with control *Pax5* ^+/+^ FO B cells (Fig 7E and Appendix Fig S7F). Importantly, this increase in p‐STAT5 was not observed in *Pax5* ^Jak2‐KD/+^ FO B cells due to inactivation of the Jak2 kinase by the K272E mutation (Fig 7E). We next analyzed B cells from the bone marrow of *Pax5* ^Jak2/+^ mice at the age of 4–5 weeks. A small but significant increase of p‐STAT5 levels was again observed in *Pax5* ^Jak2/+^ pro‐B cells compared with *Pax5* ^+/+^ pro‐B cells, whereas the p‐STAT5 levels were strongly increased in the leukemic B220^low^ B cells (Fig 7F). We therefore conclude that the strong increase of STAT5 phosphorylation occurs already in early leukemogenesis concomitant with loss of the wild‐type *Pax5* allele and increased DNA‐binding of Pax5‐Jak2. These data therefore provide strong support for a nuclear function of Pax5‐Jak2 in maintaining high levels of p‐STAT5 in *Pax5* ^Jak2/+^ B‐ALL cells.

To evaluate the role of STAT5 in controlling gene expression in *Pax5* ^Jak2/+^ B‐ALL tumors, we first generated a reference dataset by identifying STAT5‐bound and STAT5‐regulated genes in pro‐B cells. ChIP‐seq analysis of short‐term cultured pro‐B cells with an anti‐STAT5 antibody identified 2,377 STAT5 peaks by stringent MACS peak calling with a *P* value of < 10^−10^, which resulted in 1,606 STAT5‐bound genes and the identification of the consensus STAT5‐binding motif (Appendix Fig S7G). RNA‐seq analysis of *ex vivo* sorted pro‐B cells from *Vav‐Bcl2 Rag1*‐Cre *Stat5* ^fl/fl^ and control *Vav‐Bcl2 Rag1*‐Cre *Stat5* ^fl/+^ mice (Malin *et al*, 2010) identified 57 STAT5‐activated and 53 STAT5‐repressed genes with an expression difference of > 2‐fold, an adjusted *P* value of < 0.05 and an expression value of > 5 TPM in at least one of the two cell types (Appendix Fig S7H and Dataset EV4). Combining differential gene expression with STAT5 binding defined 19 activated and 4 repressed STAT5 target genes in pro‐B cells (Appendix Fig S7H). We next investigated whether the STAT5‐binding sites identified in pro‐B cells were enriched at the 254 upregulated and 144 downregulated genes in *Pax5* ^Jak2/+^ B‐ALLs (Fig 2C). Indeed, STAT5 binding was significantly increased from 8.5% of all nonregulated genes to 14.9% of the 398 regulated genes in *Pax5* ^Jak2/+^ B‐ALL tumors (Fig 7G), which was also confirmed by GSEA analysis (Appendix Fig S7I). Although we identified only 19 activated STAT5 target genes in pro‐B cells, nine of these genes were shown by GSEA to be significantly enriched as upregulated genes in *Pax5* ^Jak2/+^ B‐ALL cells compared with control *Pax5* ^+/−^ *Cdkn2ab* ^+/−^ B‐ALL cells (Fig 7H). *Cxcr5*, *Syndig1l,* and *Sema4a* are shown as representative genes that exhibited strong upregulation in *Pax5* ^Jak2/+^ B‐ALL cells (Fig 7I) and displayed STAT5‐dependent activation as well as STAT5 binding in pro‐B cells (Fig 7J and K). In summary, we conclude that the nuclear Pax5‐Jak2 kinase activity phosphorylates STAT5, which in turn activates a STAT5‐dependent gene expression program in *Pax5* ^Jak2/+^ B‐ALL cells.

## Discussion

The PAX5‐JAK2^+^ B‐ALLs belong to the Philadelphia chromosome‐like (Ph‐like) ALL subgroup, which is characterized by genetic alterations leading to constitutive activation of kinase signaling (Roberts *et al*, 2012, 2014). The PAX5‐JAK2 protein, consisting of the DNA‐binding paired domain of PAX5 fused to the kinase domain of JAK2 (Nebral *et al*, 2009), was shown to bind Pax5 recognition sequences and to function as a constitutively active kinase in established cell lines (Schinnerl *et al*, 2015). Here, we have generated a mouse model to investigate the oncogenic role of the Pax5‐Jak2 protein in leukemia formation. The *Pax5* ^Jak2/+^ mice rapidly developed an aggressive B‐ALL in the bone marrow without the need of introducing another cooperating exogenous gene mutation. Both the DNA‐binding function and kinase activity of Pax5‐Jak2 were contributing to leukemia development. Unexpectedly, the cells of all *Pax5* ^Jak2/+^ tumors analyzed lost the wild‐type *Pax5* allele, which allowed the Pax5‐Jak2 protein to efficiently bind to genomic target sites in the nucleus. The strong selection for loss of heterozygosity identified Pax5‐Jak2 as a nuclear oncoprotein and important driver of leukemia development, which functions by maintaining high levels of phosphorylated STAT5 in the nucleus.

Although Pax5‐Jak2 contains a DNA‐binding domain, it does not function as a classical transcription factor, as the Jak2 kinase domain cannot substitute for the lack of the Pax5 transactivation domain. This is best evidenced by the lack of CD19^+^ B cells due to a developmental block at an uncommitted lymphoid progenitor stage (Kit^+^B220^+^CD19^−^) in *Pax5* ^Jak2/−^ mice similar to *Pax5* ^−/−^ mice (Nutt *et al*, 1999), demonstrating that the *Pax5* ^Jak2^ allele behaves like a *Pax5* null allele with regard to Pax5 function. Like other B‐ALL types, the *Pax5* ^Jak2/+^ tumors arise in committed Pax5‐expressing B cells in the bone marrow. However, different to most other B‐ALLs, loss of the wild‐type *Pax5* allele is a genetic alteration leading to accelerated leukemia development in *Pax5* ^Jak2/+^ mice. Consequently, the leukemic *Pax5* ^Jak2/+^ cells lack normal Pax5 function, which is evidenced by the downregulated expression of activated Pax5 target genes and the reactivation of repressed Pax5 target genes in *Pax5* ^Jak2/+^ B‐ALL cells. Hence, tumor development is initiated in committed B cells, but then leads to dedifferentiation of the *Pax5* ^Jak2/+^ B‐ALL cells due to the loss of Pax5 function similar to what is observed upon conditional *Pax5* deletion in pro‐B cells (Mikkola *et al*, 2002).

By genome‐wide binding analysis, we previously demonstrated that a Pax5 protein consisting only of the DNA‐binding paired domain (Prd) competes very inefficiently with full‐length Pax5 protein for binding to genomic Pax5 target sites in *Pax5* ^Prd/+^ pro‐B cells (Smeenk *et al*, 2017). A possible explanation for this binding competition may be that full‐length Pax5 can be incorporated into transcription factor complexes through interaction via its central and C‐terminal protein sequences in contrast to the Pax5 paired domain polypeptide, which cannot be stabilized on genomic DNA by such protein interactions. Here, we have shown that the Pax5‐Jak2 protein, containing only the paired domain of Pax5, is equally inefficient in competing with full‐length Pax5 for binding to genomic Pax5 recognition sequences in *Pax5* ^Jak2/+^ pro‐B cells. Upon loss of full‐length Pax5, the Pax5‐Jak2 protein was, however, able to interact with most genomic Pax5‐binding sites in *Pax5* ^Jak2/+^ B‐ALL cells, strongly indicating that Pax5‐Jak2 functions as DNA‐binding oncoprotein in the nucleus. The potent selection pressure to lose the wild‐type *Pax5* allele in these *Pax5* ^Jak2/+^ B‐ALL cells was dependent on both the Jak2 kinase activity and the DNA‐binding function of Pax5‐Jak2, as no tumors developed in *Pax5* ^Jak2‐KD/+^ mice, while one third of the *Pax5* ^Prd^*^‐Jak2/+^ tumors still contained the wild‐type *Pax5* allele. Notably, the loss of heterozygosity in *Pax5* ^Jak2/+^ B‐ALL tumors occurred by acquired uniparental disomy through copying the *Jak2* cDNA insertion of the *Pax5* ^Jak2^ allele by interchromosomal recombination into the wild‐type *Pax5* allele. The observed oligoclonal origin of the *Pax5* ^Jak2/+^ B‐ALL tumors furthermore suggests that rare independent events of Pax5 loss by uniparental disomy were strongly selected for cell expansion to contribute to tumor development.

Loss of heterozygosity by uniparental disomy depends on homology regions on both sides of the mutant sequence to be inserted by homologous recombination into the wild‐type allele (Tuna *et al*, 2009) and was previously reported to account for the homozygosity of the *JAK2‐V617F* or *PAX5‐P80R* mutation in myeloid or B cell leukemia, respectively (Vilaine *et al*, 2011; Li *et al*, 2014; Bastian *et al*, 2019; Gu *et al*, 2019). In this regard, the mouse *Pax5* ^Jak2/+^ tumor model differs from the human PAX5‐JAK2^+^ B‐ALLs, as the *Pax5* ^Jak2^ allele was created by an in‐frame insertion of the partner *Jak2* cDNA sequences into the mouse *Pax5* locus, whereas the human *PAX5‐JAK2* rearrangements are generated by fusion of the 5’ part of the *PAX5* locus with the 3’ region of the *JAK2* locus (Nebral *et al*, 2009). Consequently, only the *Pax5* ^Jak2^ allele can undergo loss of heterozygosity by homologous recombination in marked contrast to the *PAX5‐JAK2* rearrangement. Consistent with this idea, the wild‐type *PAX5* allele was present and expressed in all 8 human PAX5‐JAK2^+^ B‐ALLs analyzed. Notably however, the expression of Pax5 from the heterologous *Ikaros* locus delayed but did not prevent B‐ALL development in *Pax5* ^Jak2/+^ mice, indicating that the Pax5‐Jak2 protein could still induce tumor development in the presence of full‐length Pax5 in the mouse, which is analogous to the situation observed with human PAX5‐JAK2^+^ B‐ALLs. Hence, the mouse *Pax5* ^Jak2/+^ model appears to generate, through loss of the wild‐type *Pax5* allele, a more aggressive leukemia and thus does not recapitulate all aspects of the human PAX5‐JAK2^+^ B‐ALL disease. However, the more aggressive mouse *Pax5* ^Jak2/+^ tumor model allowed us to identify the Pax5‐Jak2 protein as an important oncogenic driver of leukemia development, which might have been more difficult to demonstrate in the presence of the competing full‐length Pax5 protein. The *Pax5* ^Jak2/+^ mouse model also allowed us to demonstrate by genetic mutation that both the DNA‐binding function and kinase activity of the Pax5‐Jak2 protein contribute to B‐ALL development. The Jak2 kinase activity is also required for tumor progression, as treatment of *Pax5* ^Jak2/+^ tumor‐bearing mice with the JAK1/2 inhibitor ruxolitinib delayed tumor growth leading to prolonged survival. However, this treatment could not eradicate the tumor cells similar to the observed development of resistance to ruxolitinib in clinical applications (Meyer & Levine, 2014).

In addition to the canonical role of cytoplasmic JAK2 in JAK‐STAT signaling (Chen *et al*, 2012), nuclear JAK2 was identified as an “epigenetic writer” that phosphorylates histone H3 on tyrosine 41 (H3Y41ph; Dawson *et al*, 2009). H3Y41 phosphorylation was shown to prevent heterochromatin formation by interfering with HP1α binding to H3, which leads to gene activation as exemplified by the oncogenes *Lmo2* and *Myc* (Dawson *et al*, 2009; Rui *et al*, 2010). Consistent with a role in gene expression, high levels of H3Y41 phosphorylation correlate with high abundance of the active H3K4me3 mark at active promoters (Dawson *et al*, 2012; Rui *et al*, 2016). A critical reagent for obtaining these results was a rabbit polyclonal anti‐H3Y41ph antibody that is no longer commercially available. Based on these published data, we hypothesized that the nuclear Pax5‐Jak2 protein with its constitutive Jak2 kinase activity may also control gene expression by phosphorylating H3Y41. By generating a mouse monoclonal antibody that specifically detects pY41 in the H3 sequence context, we could, however, not detect Jak2‐dependent phosphorylation of H3Y41 in *Pax5* ^Jak2/+^ B‐ALL cells. Moreover, we also did not observe differences in the abundance of the active H3K4me3 or H3K27ac mark at genomic Pax5‐Jak2‐binding sites in experimental *Pax5* ^Jak2/−^ and control *Pax5* ^Prd/−^ progenitor cells. Based on these data, we therefore conclude that Pax5‐Jak2 is unlikely to control gene expression as an epigenetic regulator.

Here, we have demonstrated that Pax5‐Jak2 phosphorylates STAT5 in *Pax5* ^Jak2/+^ B‐ALL cells, consistent with previous data obtained with transfected human cell lines (Roberts *et al*, 2014; Schinnerl *et al*, 2015). While STAT5 phosphorylation was minimally induced by Pax5‐Jak2 in *Pax5* ^Jak2/+^ pro‐B cells, it was strongly increased in leukemic B220^low^ B cells of 4‐week‐old *Pax5* ^Jak2/+^ mice. Hence, the strong increase of STAT5 phosphorylation occurred already in early leukemogenesis concomitant with loss of the wild‐type *Pax5* allele and increased DNA‐binding of Pax5‐Jak2, which provides compelling evidence for a nuclear function of Pax5‐Jak2 in maintaining high levels of p‐STAT5 in *Pax5* ^Jak2/+^ B‐ALL cells. ChIP‐ and RNA‐seq analyses furthermore implicated Pax5‐Jak2 in the regulation of a STAT5‐dependent gene expression program in *Pax5* ^Jak2/+^ B‐ALL cells. In this context, it is important to note that STAT5 has been identified as an important oncogenic driver of leukemia development (Heltemes‐Harris *et al*, 2011; Katerndahl *et al*, 2017; Wingelhofer *et al*, 2018; de Araujo *et al*, 2019).

**Figure 8:**
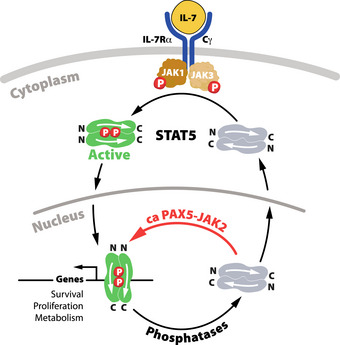
Model explaining the role of PAX5‐JAK2 in maintaining active STAT5 levels in the nucleus In wild‐type pro‐B cells, IL‐7‐mediated activation of the IL‐7 receptor, consisting of the IL‐7Rα and Cγ chains, leads to phosphorylation and activation of JAK1 and JAK3. These active kinases phosphorylate STAT5, which results in the formation of parallel STAT5 dimers that are transported across the nuclear membrane by importins. In the nucleus, STAT5 binds to DNA and regulates genes involved in cell survival, proliferation, and metabolism (Villarino *et al*, 2017). Phosphatase‐mediated inactivation of the DNA‐bound STAT5 complex disengages the dimer from the DNA, followed by formation of the anti‐parallel STAT5 dimer and its export via nuclear exportins to the cytoplasm. In *Pax5* ^Jak2/+^ B‐ALL cells, cytoplasmic STAT5 is similarly activated by IL‐7R signaling and transported across the nuclear membrane. In the nucleus however, the constitutively active (ca) PAX5‐JAK2 protein antagonizes the action of phosphatases by re‐phosphorylating STAT5 and thus maintaining active STAT5 levels, leading to sustained expression of STAT5 target genes, which promotes leukemogenesis.

The almost exclusive localization of Pax5‐Jak2 in the nucleus raises the question of how nuclear Pax5‐Jak2 can promote gene expression, if newly synthesized and unphosphorylated STAT5 needs to be phosphorylated by Jak kinases in the cytoplasm in order to relocate to the nucleus in response to cytokine signaling (Villarino *et al*, 2017) (Fig 8). Notably, the *Pax5* ^Jak2/+^ B‐ALL cells still depend on IL‐7 signaling even in the presence of the constitutively active Pax5‐Jak2 protein, which may explain why the *Pax5* ^Jak2/+^ tumors are preferentially located in the IL‐7‐rich environment of lymph nodes (Link *et al*, 2007). Based on this finding, we propose the following model for the nuclear function of Pax5‐Jak2 (Fig 8). In control pro‐B cells, IL‐7 signaling promotes Jak1,3‐dependent STAT5 phosphorylation, which leads to parallel dimer formation and nuclear transfer of p‐STAT5. In the nucleus, p‐STAT5 promotes gene expression until it is dephosphorylated and relocated to the cytoplasm to initiate a new cycle of phosphorylation and nuclear transfer in response to IL‐7 signaling. In *Pax5* ^Jak2/+^ B‐ALL cells, IL‐7 signaling is also responsible for the transfer of p‐STAT5 from the cytoplasm to the nucleus. However, p‐STAT5 can now be maintained at a higher level in the nucleus by the constitutively active Pax5‐Jak2 kinase, which antagonizes the action of nuclear phosphatases by re‐phosphorylating STAT5, thus leading to continuous expression of STAT5 target genes. The DNA‐binding function of Pax5‐Jak2 likely contributes to the nuclear Jak2 activity by retaining the fusion protein in the nucleus either through specific recognition of Pax5‐binding sites or through a more general DNA‐binding mode. This paradigm explaining the nuclear function of Pax5‐Jak2 may also be valid for all other 13 nuclear JAK2 fusion proteins identified in human B‐ALL, as they also retain the catalytic kinase domain (JH1) of JAK2 (Roberts *et al*, 2014; Akkari *et al*, 2020).

Finally, B‐ALL development requires at least two cooperating mutations, as exemplified by the constitutive activation (ca) of STAT5 combined with heterozygous loss of *Pax5* in transgenic *caStat5a Pax5* ^+/−^ and *caStat5b Pax5* ^+/−^ mice (Heltemes‐Harris *et al*, 2011; Smeenk *et al*, 2017). In contrast, the *Pax5* ^Jak2^ allele behaves like a dual‐hit mutation, as it gives rise to the expression of a constitutively active Jak2 kinase in the absence of Pax5 function. In summary, our detailed analysis of the *Pax5* ^Jak2/+^ mouse model has identified the *PAX5‐JAK2* rearrangement as a dual‐hit mutation that promotes B‐ALL development by maintaining high STAT5 activity in the nucleus.

## Material and Methods

Detailed methods can be found in the Appendix Supplementary Methods available online.

### Mice

The following mice were maintained on the C57BL/6 genetic background: *Pax5* ^+/−^ (Urbánek *et al*, 1994), *Pax5* ^ihCd2/ihCd2^ (Fuxa & Busslinger, 2007), *Pax5* ^Bio/Bio^ (McManus *et al*, 2011), *Pax5* ^Prd/+^ (Smeenk *et al*, 2017), *Pax5* ^Etv6/+^ (Smeenk *et al*, 2017), *Cdkn2ab* ^+/−^ (Krimpenfort *et al*, 2007), *Il7* ^+/−^ (von Freeden‐Jeffry *et al*, 1995), *Rosa26* ^BirA/BirA^ (Driegen *et al*, 2005), *Ikzf1* ^neo/+^ (Souabni *et al*, 2002), *Stat5* ^fl/fl^ (Cui *et al*, 2004), *Cd79a*(Mb1)^Cre/+^ (Hobeika *et al*, 2006), *Meox2* ^Cre/+^ (Tallquist & Soriano, 2000), *Rag1* ^Cre/+^ (McCormack *et al*, 2003), transgenic FLPe (Rodriguez *et al*, 2000), transgenic CAGGs‐Dre (Anastassiadis *et al*, 2009), and transgenic *Vav*‐Bcl2 (Ogilvy *et al*, 1999). The *Meox2* ^Cre/+^, *Rag1* ^Cre/+^ and *Cd79a* ^Cre/+^ genotypes are referred to as *Meox2*‐Cre, *Rag1*‐Cre and *Cd79a*‐Cre, respectively. All animal experiments were carried out according to valid project licenses, which were approved and regularly controlled by the Austrian Veterinary Authorities.

### Generation of *Pax5* ^Jak2/+^, *Pax5* ^Jak2‐Luc/+^, *Pax5* ^Prd^*^‐Jak2/+^, and *Pax5* ^Jak2‐KD/+^ mice

The *Pax5* ^Jak2‐Luc^ and *Pax5* ^Jak2^ alleles were generated by ES cell targeting using the targeting vector shown in Appendix Fig S1B and described in detail in Appendix Supplementary Methods. The *Pax5* ^LSL‐Jak2‐KD/+^ and *Pax5* ^LSL‐Prd^*^‐Jak2/+^ mice were generated by CRISPR/Cas9‐mediated genome editing in mouse *Pax5* ^LSL‐Jak2/+^ zygotes that were injected with Cas9 mRNA, an sgRNA specific for the sequence to be mutated and a single‐stranded DNA repair template of 200 nucleotides (Appendix Fig S4A and S5E). The *Pax5* ^Jak2‐Luc/+^ and *Pax5* ^Jak2/+^ mice were generated by deletion of the LSL cassette with *Meox2*‐Cre.

### Antibodies

The following antibodies were used for flow‐cytometric analysis: B220/CD45R (RA3‐6B2), CD2 (RM2‐5), CD11b/Mac1 (M1/70), CD19 (1D3), CD21/CD35 (7G6), CD23 (B3B4), CD25/IL‐2Rα (PC61), CD93/AA4.1 (AA4.1), CD117/c‐Kit (2B8), CD127/IL‐7Rα (A7R34), CD135/Flt3 (A2F10), Gr1 (RB6‐8C5), IgD (11.26c), Igκ (187.1), IgM (II/41 or eB121‐15F9), and TCRβ (H57‐597).

The anti‐Pax5 (C‐terminal; D19F8; Cell Signaling Technology) anti‐p‐STAT5 (47/Stat5 pY694; BD Biosciences) antibodies were used for intracellular staining. The following antibodies were used for immunoblot analysis: anti‐Pax5 (directed against amino acids 17–145; Adams *et al*, 1992), anti‐p‐STAT5 (C11C5 pY694; Cell Signaling Technology), anti‐Tbp (3TF1‐3G3; Active Motif), anti‐H3, HRP‐coupled (D1H2; Cell Signaling Technology), and anti‐Gapdh, HRP‐coupled (14C10; Cell Signaling Technology). The following antibodies were used for ChIP analysis: anti‐STAT5A and anti‐STAT5B (PA‐ST5A and PA‐ST5B, R&D Systems), anti‐H3K4me3 (pAb‐003‐050; Diagenode), anti‐H3K27ac (ab4729, Abcam), and anti‐H3K27me3 (C36B11; Cell Signaling Technology) antibody.

### Generation of a monoclonal H3Y41ph‐specific antibody

Fifty milligrams of the phosphorylated H3 peptide (amino acids 37–46 with phosphorylated (p) Y41; Fig 6A) in complete Freund’s adjuvant were subcutaneously injected into one mouse. After 3 immunizations, 30 mg of the phosphorylated H3 peptide (without adjuvant) were intravenously injected as final boost. After 4 days, splenocytes were fused with the myeloma cell line X63‐Ag8.653 by using polyethylene glycol. The cells were seeded into 96‐well plates, and fused hybridoma cells were selected in HAT‐containing growth medium. Hybridoma supernatants were screened for H3Y41ph‐specific antibodies by ELISA against unmodified or pY41‐modified H3 peptides (amino acids 29–53, Fig 6A).

### Flow cytometric sorting and definition of mouse hematopoietic cell types

Cell types were defined as follows: Pax5‐deficient progenitors (CD19^−^B220^+^Kit^+^Ly6D^+^), pro‐B cells (CD19^+^B220^+^Kit^+^CD2^−^IgM^−^IgD^−^), pre‐B cells (CD19^+^B220^+^Kit^−^CD2^+^IgM^−^IgD^−^), large pre‐B cells (CD19^+^B220^+^Kit^−^IgM^−^IgD^−^FSC^hi^), small pre‐B cells (CD19^+^B220^+^Kit^−^IgM^−^IgD^−^FSC^lo^), immature B cells (CD19^+^B220^+^IgM^+^IgD^−^), recirculating B cells (CD19^+^B220^+^IgD^+^), mature splenic B cells (CD19^+^B220^+^IgD^+^), follicular (FO) B cells (CD19^+^B220^+^CD23^+^CD21^lo^), marginal zone (MZ) B cells (CD19^+^B220^+^CD21^hi^CD23^lo^), T cells (TCRβ^+^), myeloid cells (CD11b^+^Gr1^+^).

### Transplantation experiments and ruxolitinib treatment

Tumor cells from lymph nodes of moribund *Pax5* ^Jak2/+^ and *Pax5* ^Jak2‐Luc/+^ mice as well as B220^low^ and B220^+^ B cells from the bone marrow of *Pax5* ^Jak2/+^ mice were sorted by flow cytometry and transferred by intravenous injection (10^5^ cells per mouse) into sublethally irradiated C57BL/6 mice (4.5 Gy). Ruxolitinib (R‐6688; LC Laboratories) was administered twice daily by oral gavage of 45 mg/kg in 0.5% methylcellulose (M0512; Sigma). For bioluminescence imaging, mice were intraperitoneally injected with D‐luciferin (150 mg/kg, Goldbio) and imaged with an IVIS Spectrum Xenogen machine (Caliper Life Sciences).

### *In vitro* culture of pro‐B cells and B‐ALL cell lines

Pro‐B cells were cultured on OP9 feeder cells in IL7‐containing IMDM as described (Nutt *et al*, 1997). B‐ALL cells were isolated from lymph nodes of *Pax5* ^Jak2/+^ *Rosa26* ^BirA/+^ or *Pax5* ^Etv6/+^ *Cdkn2ab* ^+/−^ tumor mice and were established as cell lines by culturing them on ST2 feeder cells in IL7‐containing IMDM medium.

### ChIP analysis of Pax5‐Jak2 and STAT5 binding

*Ex vivo* B‐ALL cells from *Pax5* ^Jak2/+^ *Rosa26* ^BirA/+^ tumor mice, *ex vivo* sorted *Pax5* ^Bio/Bio^ pro‐B cells, short‐term cultured *Pax5* ^Jak2/+^ *Rosa26* ^BirA/+^ pro‐B cells, and short‐term cultured *Pax5* ^Jak2/−^ and *Pax5* ^Prd/−^ progenitor cells were crosslinked at room temperature with 1% formaldehyde (Sigma) for 10 min. The sheared chromatin was immunoprecipitated with specific antibodies or by streptavidin‐mediated pull‐down of *in vivo* biotinylated Pax5 and Pax5‐Jak2 proteins, as described (Ebert *et al*, 2011; Revilla‐i‐Domingo *et al*, 2012). The precipitated DNA at selected genomic sites was determined by qPCR analysis (Table EV1). For analysis of STAT5 binding, wild‐type pro‐B cells were cultured on OP9 cells in the presence of IL‐7, before IL‐7 was withdrawn for 4 h and the pro‐B cells were restimulated with IL‐7 (10 ng/ml) for 30 min prior to formaldehyde fixation, nuclei preparation, and ChIP analysis with a mixture of anti‐STAT5A and anti‐STAT5B antibodies. ChIP‐precipitated DNA (0.5–5 ng) was used for library preparation and Illumina deep sequencing.

### RNA‐sequencing

RNA from *ex vivo* sorted pro‐B cells and B‐ALL cells was isolated with the RNeasy Plus Mini Kit (Qiagen). mRNA was obtained by two rounds of poly(A) selection and used for library preparation and Illumina deep sequencing, as described (Smeenk *et al*, 2017).

### Bioinformatic analysis of RNA‐ and ChIP‐seq data

Bioinformatic analysis of the RNA‐ and ChIP‐seq data was performed as described in Smeenk *et al* (2017) and in Appendix Supplementary Methods.

### Statistical analysis

Statistical analysis was performed with the GraphPad Prism 8 software. Two‐tailed unpaired Student’s *t*‐test analysis and multiple *t*‐tests (unpaired and two‐tailed with Holm‐Šídák's correction) were used to assess the statistical significance of one observed parameter between two experimental groups. For comparison of multiple groups and analysis of repeated measurements, the ANOVA analysis or mixed model effect analysis (REML) were used together with post‐hoc multiple comparison tests (Šídák or Tukey, for two or more groups, respectively).

## Author contributions

SJ performed most experiments; ASF carried out p‐STAT5 analyses; DK‐P generated the *Pax5* ^LSL‐Jak2‐Luc‐Neo^ allele by ES cell targeting; SGM performed the ChIP‐ and RNA‐seq experiments to identify regulated STAT5 target genes; CGM provided PAX5‐JAK2^+^ B‐ALL RNA‐seq data; SS provided human PAX5‐JAK2^+^ B‐ALL samples and advice on these tumors; MF and MJ performed the bioinformatic analysis of the RNA‐seq and ChIP‐seq data, respectively; SJ and MB planned the project, designed the experiments, and wrote the manuscript.

## Supporting information



Appendix

Table EV1

Table EV2

Dataset EV1

Dataset EV2

Dataset EV3

Dataset EV4

Source Data for Appendix

Source Data for Figure 1

Source Data for Figure 3

Source Data for Figure 6

## Data Availability

The RNA‐seq and ChIP‐seq data generated for this study (Table EV2) are available at the Gene Expression Omnibus (GEO) repository under the accession number GSE174775 (http://www.ncbi.nlm.nih.gov/geo/query/acc.cgi?acc=GSE174775). The human RNA‐seq data are available upon request.
