# Supplementary material for: The PAX5‐JAK2 translocation acts as dual‐hit mutation that promotes aggressive B‐cell leukemia via nuclear STAT5 activation
Source: EMBO J. 2022 Feb 14;41(7):e108397. doi: 10.15252/embj.2021108397 (PMC8982625; doi:10.15252/embj.2021108397)
Supplement: Supplementary file 1 — Appendix [file EMBJ-41-e108397-s008.pdf]

Appendix

The PAX5-JAK2 translocation acts as dual-hit mutation that promotes aggressive B-cell leukemia via nuclear STAT5 activation

Sabine Jurado, Anna S. Fedl, Markus Jaritz, Daniela Kostanova-Poliakova, Stephen G. Malin, Charles G. Mullighan, Sabine Strehl, Maria Fischer and Meinrad Busslinger

Table of content

1. Appendix Figures (pages 2 – 14)
2. Appendix Supplementary Methods (pages 15 – 23)
3. Appendix Supplementary References (pages 24 – 26)

1. Appendix Figures

Appendix Figure S1

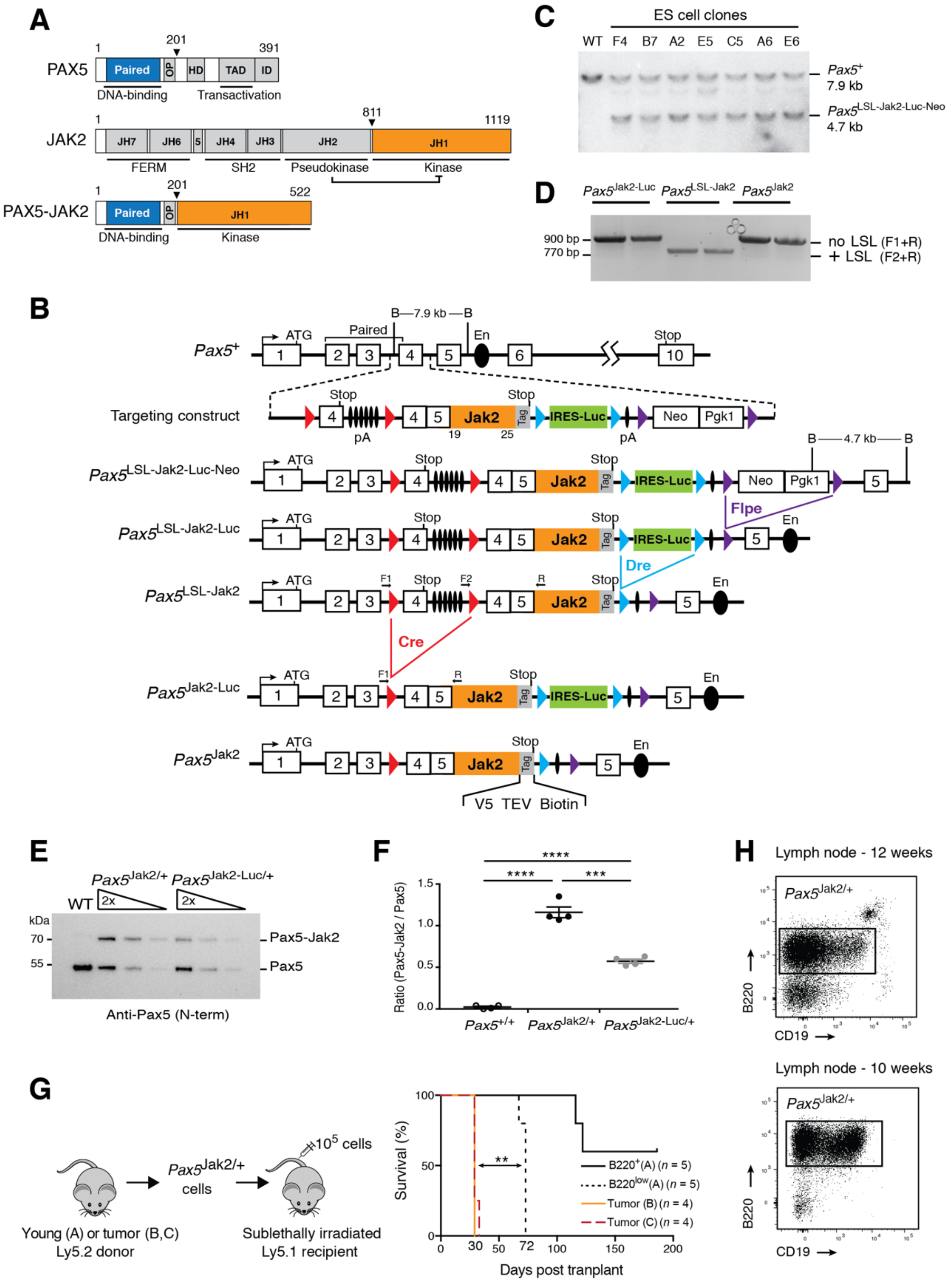

Appendix Figure S1. Generation and characterization of the *Pax5*$^{\mathbf{Jak2}}$ and Pax5$^{\mathbf{Jak2\text{-}Luc}}$ alleles.

(A) Schematic diagram indicating the domain structure of the human PAX5, JAK2 and PAX5-JAK2 proteins. Arrowheads indicate the junction (amino acid residue) where PAX5 and JAK2 are fused. The JAK homology (JH), FERM, SH2, pseudokinase and kinase domains of JAK2 as well as the paired domain, octapeptide (OP) motif, partial homeodomain (HD), transactivation domain (TAD) and inhibitory domain (ID) of PAX5 are indicated. **(B)** Generation of the *Pax5*$^{\text{Jak2}}$ and *Pax5*$^{\text{Jak2-Luc}}$ alleles. The *Pax5*$^{\text{LSL-Jak2-Luc-Neo}}$ allele was generated by replacing exon 4 of *Pax5* with the following sequences in the 5' to 3' direction: (1) a *loxP*-flanked 1.6-kb DNA fragment containing exon 4 linked to a stop codon and six copies of the SV40 polyadenylation region, (2) a 1.5-kb DNA fragment containing the 3' splice site and start of *Pax5* exon 4 linked to human cDNA sequences starting in *PAX5* exon 4 and encoding the remaining PAX5-JAK2 protein coupled to a C-terminal tag sequence, (3) a *rox*-flanked 2.3-kb DNA fragment containing the IRES-luciferase gene and (4) a 2.1-kb DNA fragment containing a SV40 polyadenylation (pA) region and *frt*-flanked mouse phosphoglycerate kinase (*Pgk1*) promoter linked to the neomycin (*neo*^{r}) resistance gene. *LoxP*, *rox* and *frt* sites are indicated by red, blue and purple arrowheads, respectively. The C-terminal tag sequences contain an epitope for V5 antibodies, two cleavage sites for the TEV protease and a biotin acceptor sequence (Biotin) for biotinylation by the *E. coli* biotin ligase BirA. The mouse *Pax5* exons are numbered and shown as open boxes. The numbers of the exons coding for the start and end of the human *JAK2* cDNA sequences are indicated. A black oval denotes the B cell-specific enhancer (En) in *Pax5* intron 5. The BglI (B) fragments of the *Pax5*$^{+}$ and *Pax5*$^{\text{LSL-Jak2-Luc-Neo}}$ alleles, which were used for allele identification by Southern blot analysis with a *Pax5* exon 5 probe, are shown together with their length (in kilobases, kb). The *Pax5*$^{\text{Jak2-Luc}}$ and *Pax5*$^{\text{Jak2}}$ alleles were generated *in vivo* by sequential Flpe-, Dre- and Cre-mediated deletion of the *Pax5*$^{\text{LSL-Jak2-Luc-Neo}}$ allele, respectively. The locations of forward (F1, F2) and reverse (R) primers used for PCR genotyping are shown. **(C)** Southern blot analysis of wild-type (WT) ES cells and 7 correctly targeted ES cell clones by hybridization of BglI-digested DNA with a *Pax5* exon 5 probe. **(D)** Efficient deletion of the *lox*P-stop-*lox*P (LSL) cassette in cells of *Pax5*$^{\text{Jak2/+}}$ and *Pax5*$^{\text{Jak2-Luc/+}}$ mice, as shown by PCR analysis of DNA from toe tissue. DNA from *Pax5*$^{\text{LSL-Jak2 /+}}$ mice was used as a control. **(E, F)** Expression of Pax5-Jak2 in short-term cultured pro-B cells from 3-week-old *Pax5*$^{\text{Jak2/+}}$ and *Pax5*$^{\text{Jak2-Luc/+}}$ mice. Two-fold serially diluted nuclear extracts were analyzed by immunoblotting with an anti-Pax5 antibody recognizing the N-terminal paired domain. One of four experiments is shown **(E)**. The ratio between the Pax5-Jak2 and Pax5 proteins was determined by densitometric quantification of 4 biological replicates **(F)**. Statistical data are shown as mean value with SEM and were analyzed by one-way ANOVA with Tukey's multiple comparison test: ***$P < 0.001$, ****$P < 0.0001$. (**G**) Analysis of tumor development by transplantation. B220^{low} pre-leukemic and B220^{+} B cells (10^5) from one 6-week-old mouse (A) as well as lymph node cells (10^5) from two *Pax5*$^{\text{Jak2/+}}$ tumor mice (B and C) were intravenously injected into sub-lethally irradiated C57BL/6 (Ly5.1) mice. Tumor development in the recipient mice was recorded by determining the appearance of enlarged lymph nodes, and the data are presented as Kaplan-Meier survival curves. Statistical analysis of the survival curves was performed with the log-rank (Mantel-Cox) test; **$P < 0.01$. **(H)** Flow-cytometric analysis of CD19 and B220 expression on B-ALL cells from the lymph nodes of two different *Pax5*$^{\text{Jak2/+}}$ tumor mice at the age of 10 and 12 weeks.

Appendix Figure S2

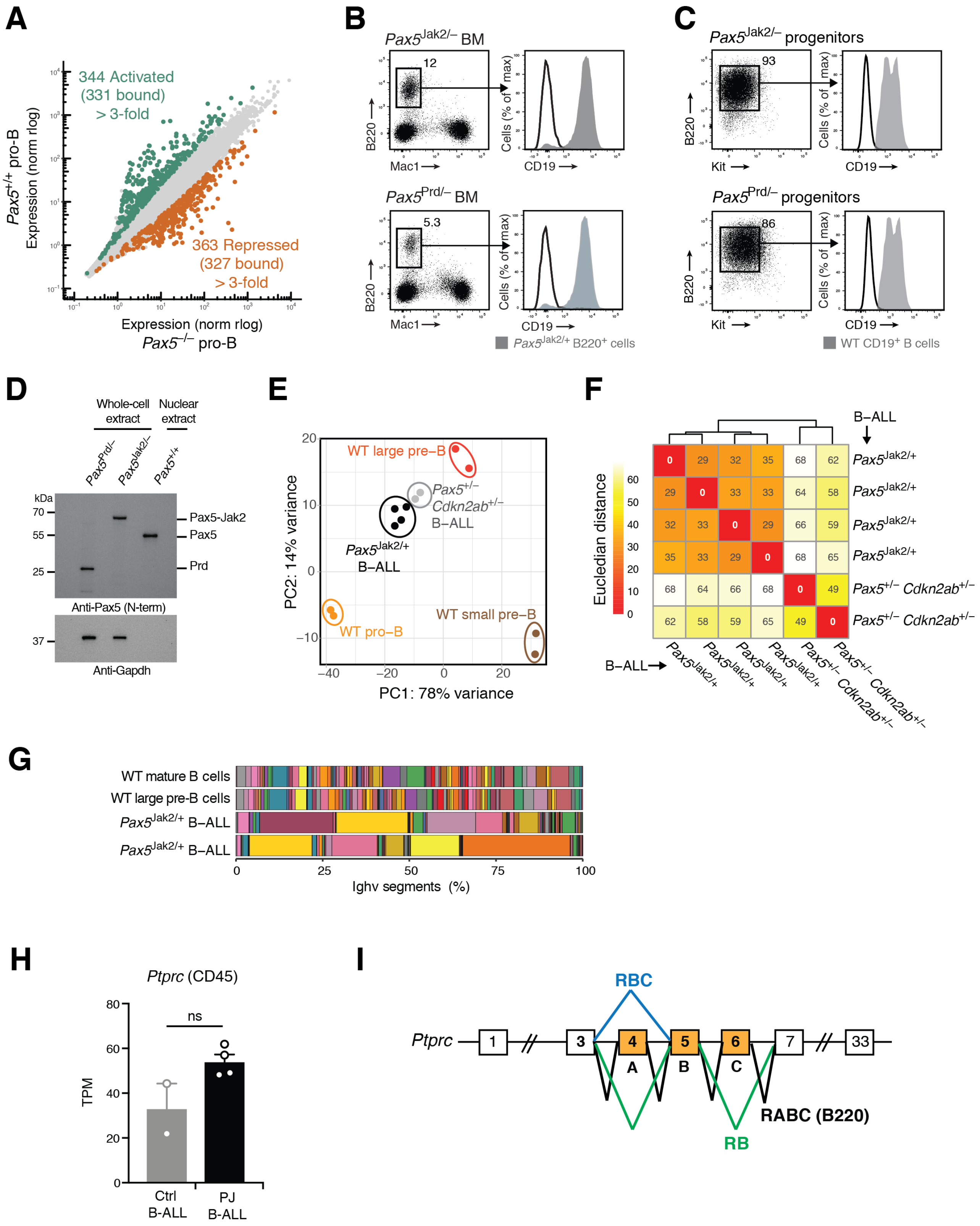

Appendix Figure S2. Pax5 target genes in pro-B cells and gene expression in *Pax5*$^{\mathrm{Jak2/+}}$ B-ALL cells.

(A) Scatter plot of gene expression differences between *Pax5*$^{+/+}$ and *Pax5*$^{-/-}$ pro-B cells, which were *ex vivo* sorted from the bone marrow prior to RNA-seq analysis. The expression data of individual genes (indicated by dots) are plotted as mean normalized rlog (regularized logarithm) values. Genes with an expression difference of > 3-fold, an adjusted *P* value of < 0.05 and a TPM value of > 5 (in at least one pro-B cell type) are colored in green and orange, corresponding to activation or repression by Pax5, respectively (Dataset EV2). Pax5-bound genes are indicated, based on the determination of the genome-wide Pax5 binding by Bio-ChIP-seq analysis of *ex vivo* sorted *Pax5*$^{\mathrm{Bio/Bio}}$ *Rag1*$^{\mathrm{Cre/Cre}}$ pro-B cells (see Methods). **(B)** Absence of CD19^{+} B cells in the bone marrow of *Pax5*$^{\mathrm{Jak2/-}}$ and *Pax5*$^{\mathrm{Prd/-}}$ mice at the age of 2 weeks, as shown by flow-cytometric analysis. CD19 expression of B220^{+} B cells from a *Pax5*$^{\mathrm{Jak2/+}}$ mouse (grey) is shown as a control. **(C)** Flow-cytometric analysis of short-term cultured *Pax5*$^{\mathrm{Jak2/-}}$ and *Pax5*$^{\mathrm{Prd/-}}$ progenitor cells, which failed to express the Pax5 target gene *Cd19*. CD19^{+} B cells (grey) from the bone marrow of a wild-type (WT) mouse were analyzed as a control. **(D)** Expression of Pax5-Jak2 and the paired domain (Prd) polypeptide in short-term cultured *Pax5*$^{\mathrm{Jak2/-}}$ and *Pax5*$^{\mathrm{Prd/-}}$ progenitor cells. Whole-cell extracts of *Pax5*$^{\mathrm{Jak2/-}}$ and *Pax5*$^{\mathrm{Prd/-}}$ progenitor cells as well as a nuclear extract of wild-type pro-B cell were analyzed by immunoblotting with an anti-Pax5 paired domain antibody and anti-Gapdh antibody. **(E)** Principal component analysis based on 2-4 RNA-seq experiments performed with the indicated *ex vivo* sorted B-ALL and early B cell types. The RNA-seq data of pro-B, large pre-B and small pre-B cells from wild-type (WT) bone marrow were previously published (Smeenk et al., EMBO J. *36*, 718-735). **(F)** Expression differences between the *Pax5*$^{\mathrm{Jak2/+}}$ and control *Pax5*$^{+/-}$ *Cdkn2ab*$^{+/-}$ B-ALL tumors, as shown by their separate clustering based on RNA-seq data. **(G)** Oligoclonality of *Pax5*$^{\mathrm{Jak2/+}}$ B-ALL tumors. The expression of each V_H gene across the *Igh* locus was determined as TPM value by RNA-seq and was displayed as a percentage of the total expression of all V_H genes in two B-ALL tumors, wild-type large pre-B and mature B cells. **(H)** Expression of the *Ptprc* (CD45) gene in *Pax5*$^{\mathrm{Jak2/+}}$ (PJ) and control (Ctrl) *Pax5*$^{+/-}$ *Cdkn2ab*$^{+/-}$ B-ALL cells. TPM, transcripts per million. **(I)** Schematic diagram indicating the predominant alternative splicing that was detected at the *Ptprc* locus in B220^{+} *Pax5*$^{+/-}$ *Cdkn2ab*$^{+/-}$ B-ALL cells (RABC isoform) and B220^{low} *Pax5*$^{\mathrm{Jak2/+}}$ B-ALL cells (RBC and RB) by RNA-seq (Fig 2H).

Appendix Figure S3

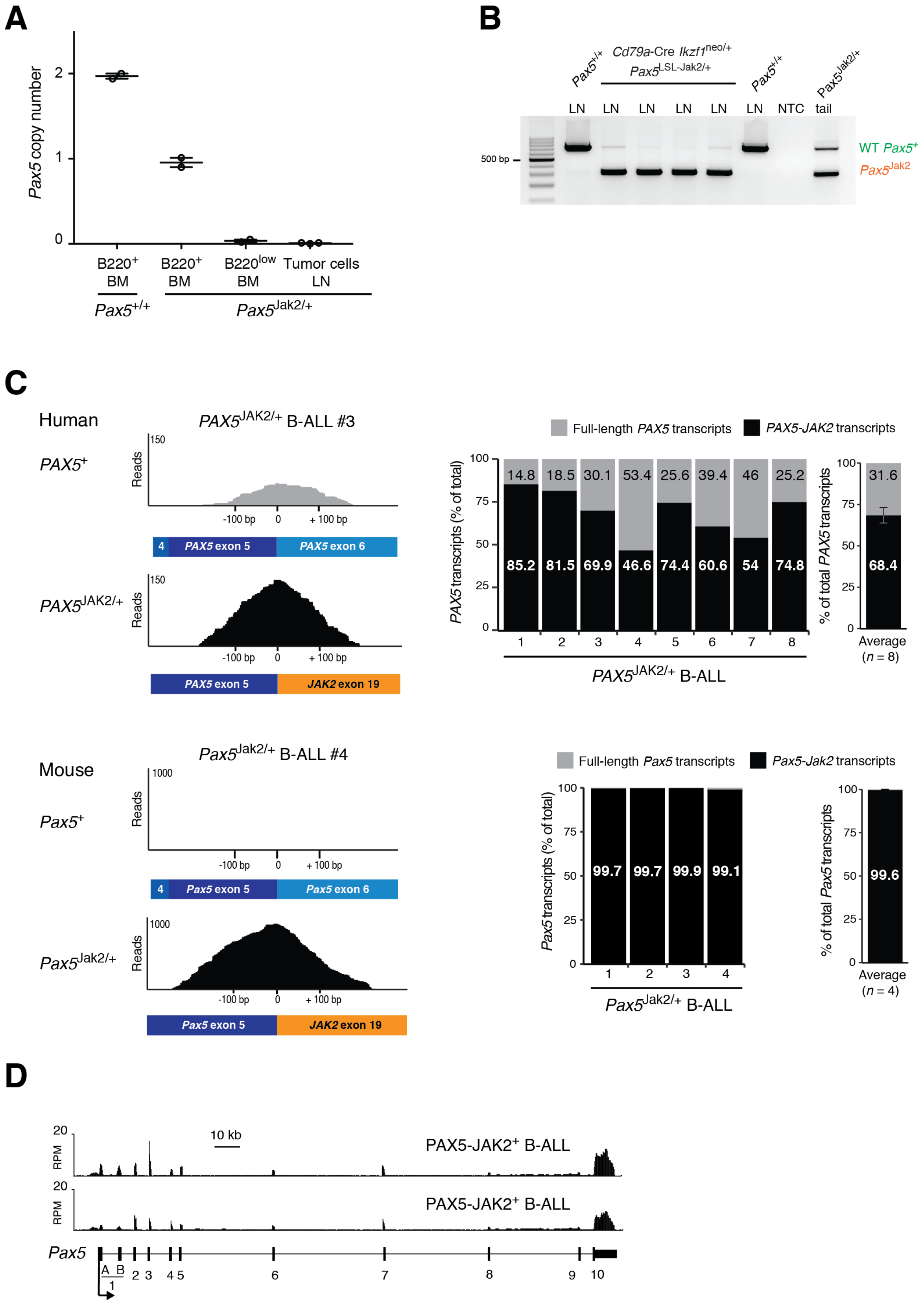

Appendix Figure S3. The *PAX5* gene is not lost in human PAX5-JAK2^{+} B-ALL tumors.

(**A**) Copy number analysis of the wild-type *Pax5* allele by quantitative PCR analysis with primers amplifying a unique 150-bp fragment of Pax5 intron 3 that is absent on the *Pax5*^{Jak2} allele. The PCR analysis was performed with genomic DNA isolated from lymph node (LN) *Pax5*$^{Jak2/+}$ B-ALL cells, from flow cytometry-sorted CD19^{+}B220^{+} and CD19^{+}B220^{low} B cells from the bone marrow (BM) of 6-week-old *Pax5*$^{Jak2/+}$ mice and from CD19^{+}B220^{+} B cells of *Pax5*$^{+/+}$ bone marrow. The PCR data were normalized to the product amplified from a control region located 22 Mb downstream of *Pax5*, and the ratio obtained for B220^{+} B cells of *Pax5*$^{Jak2/+}$ bone marrow was set to 1 copy number of the *Pax5* allele. Statistical data are shown as mean value with SEM and were analyzed by one-way ANOVA with Tukey's multiple comparison test: ns $P > 0.05$, ****$P < 0.0001$. (**B**) Loss of the *Pax5* allele in lymph node B-ALL cells from *Cd79a*-Cre *Ikzf1*$^{neo/+}$ *Pax5*$^{LSL\text{-}Jak2/+}$ tumor mice. PCR amplification was performed with primers amplifying exon 4 of the wild-type Pax5 allele and unique sequences of the *Pax5*^{Jak2} allele, as shown in the schematic diagram of Fig 3E. The positions of the PCR fragments corresponding to the two *Pax5* alleles are indicated to the right of the agarose gel. (**C**) Detection of the *PAX5* and *PAX5-JAK2* transcripts in 8 human PAX5-JAK2^{+} B-ALL samples that were analyzed by RNA-seq. The *PAX5* transcript (grey) was identified by analyzing its unique junction between *PAX5* exons 5 and 6, while the *PAX5-JAK2* transcript (black) was characterized by its unique junction between *PAX5* exon 5 and *JAK2* exon 19. The sequence data of four mouse *Pax5*$^{Jak2/+}$ B-ALL tumors were similarly analyzed. The distribution of the sequence reads at the relevant junction is shown for one representative human and mouse B-ALL tumor (left). The frequency of the *PAX5-JAK2* and *PAX5* transcripts in each human and mouse B-ALL tumor analyzed is shown together with the average frequency of all tumors (right). (**D**) RNA-seq expression profile at the *PAX5* locus in 2 representative PAX5-JAK2^{+} B-ALL tumors. The exon-intron structure and the two alternative promoters at exons 1A and 1B of *PAX5* are shown below.

Appendix Figure S4

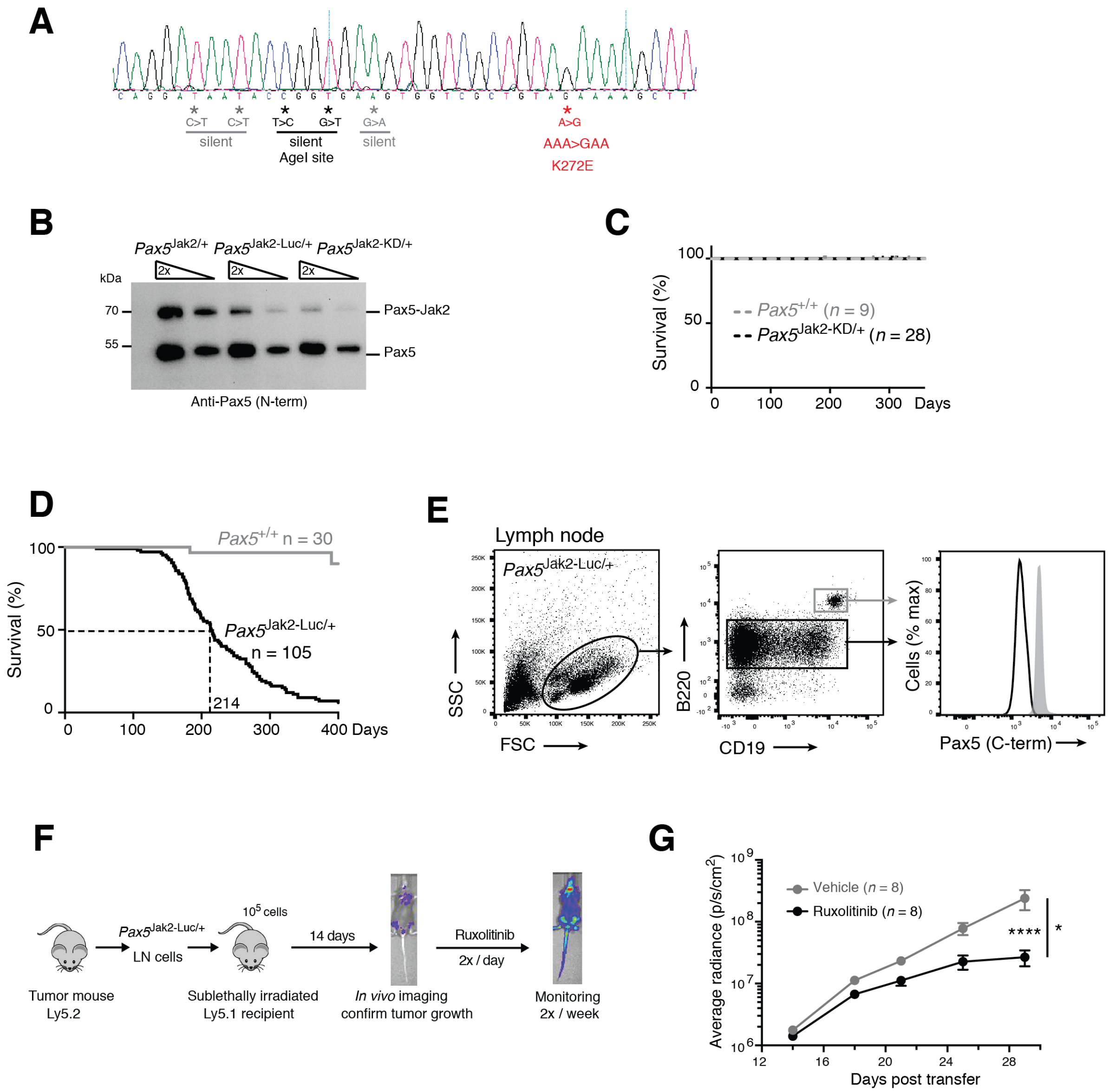

Appendix Figure S4. The development and maintenance of *Pax5*$^{\text{Jak2/+}}$ tumors depends on the Jak2 kinase activity.

(A) Generation of the *Pax5*$^{\text{Jak2-KD}}$ allele by introducing the K272E mutation into the *Pax5*$^{\text{LSL-Jak2}}$ allele by CRISPR/Cas9-mediated mutagenesis in injected *Pax5*$^{\text{LSL-Jak2/+}}$ zygotes (see Appendix Supplementary Methods). The introduced mutation was verified by PCR amplification, cloning and Sanger sequencing of the respective genomic DNA fragment. In addition to the K272E mutation (red), five silent mutations (grey) were introduced to prevent Cas9 cleavage and to generate an AgeI restriction site (ACCGGT) for genotyping. **(B)** Expression of Pax5-Jak2 in short-term cultured pro-

B cells from the bone marrow of 2-3-week-old *Pax5*$^{Jak2/+}$, *Pax5*$^{Jak2\text{-}Luc/+}$ and *Pax5*$^{Jak2\text{-}KD/+}$ mice. Two-fold serially diluted nuclear extracts were analyzed by immunoblotting with an anti-Pax5 antibody recognizing the N-terminal paired domain. One of three experiments is shown. **(C)** Kaplan-Meier survival analysis of *Pax5*$^{Jak2\text{-}KD/+}$ (black dashed) and control *Pax5*$^{+/+}$ (grey dashed) mice. *n*, number of mice analyzed. **(D)** Kaplan-Meier survival analysis of *Pax5*$^{Jak2\text{-}Luc/+}$ (black) and control *Pax5*$^{+/+}$ (grey) mice. A *P* value of < 0.0001 was determined for the survival curves by statistical analysis with the log-rank (Mantel-Cox) test. **(E)** Flow cytometry analysis of Pax5 protein expression by intracellular Pax5 staining of B220^{+}CD19^{+} B cells (grey filled) and B220^{low}CD19$^{low/-}$ B-ALL cells (black) from the lymph node of a *Pax5*$^{Jak2\text{-}Luc/+}$ tumor mouse at the age of 4 months. The full-length Pax5 protein was detected with an antibody that recognizes C-terminal Pax5 sequences that are absent in the Pax5-Jak2 protein. **(F)** Schematic diagram describing the experimental outline of the ruxolitinib inhibitor experiments. B220^{low} B-ALL cells were sorted by flow cytometry from the lymph node of moribund *Pax5*$^{Jak2\text{-}Luc/+}$ mice (Ly5.2^{+}), and 10^5 cells were intravenously transferred into sublethally irradiated Ly5.1^{+} C57BL/6 recipient mice. Tumor cell engraftment was verified by *in vivo* bioluminescence measurement 14 days post transfer, followed by twice-daily treatment with either ruxolitinib or vehicle. **(G)** Second experiment measuring the tumor progression in mice, transplanted with *Pax5*$^{Jak2\text{-}Luc/+}$ B-ALL cells, in the presence or absence of ruxolitinib as described in (**F**). The bioluminescence measurements at the indicated days after cell transfer are shown as mean radiance values with SEM. Statistical data are shown as mean value with SEM and were analyzed by two-way ANOVA with Šídák's multiple comparison test: **P* < 0.05, *****P* < 0.0001.

Appendix Figure S5

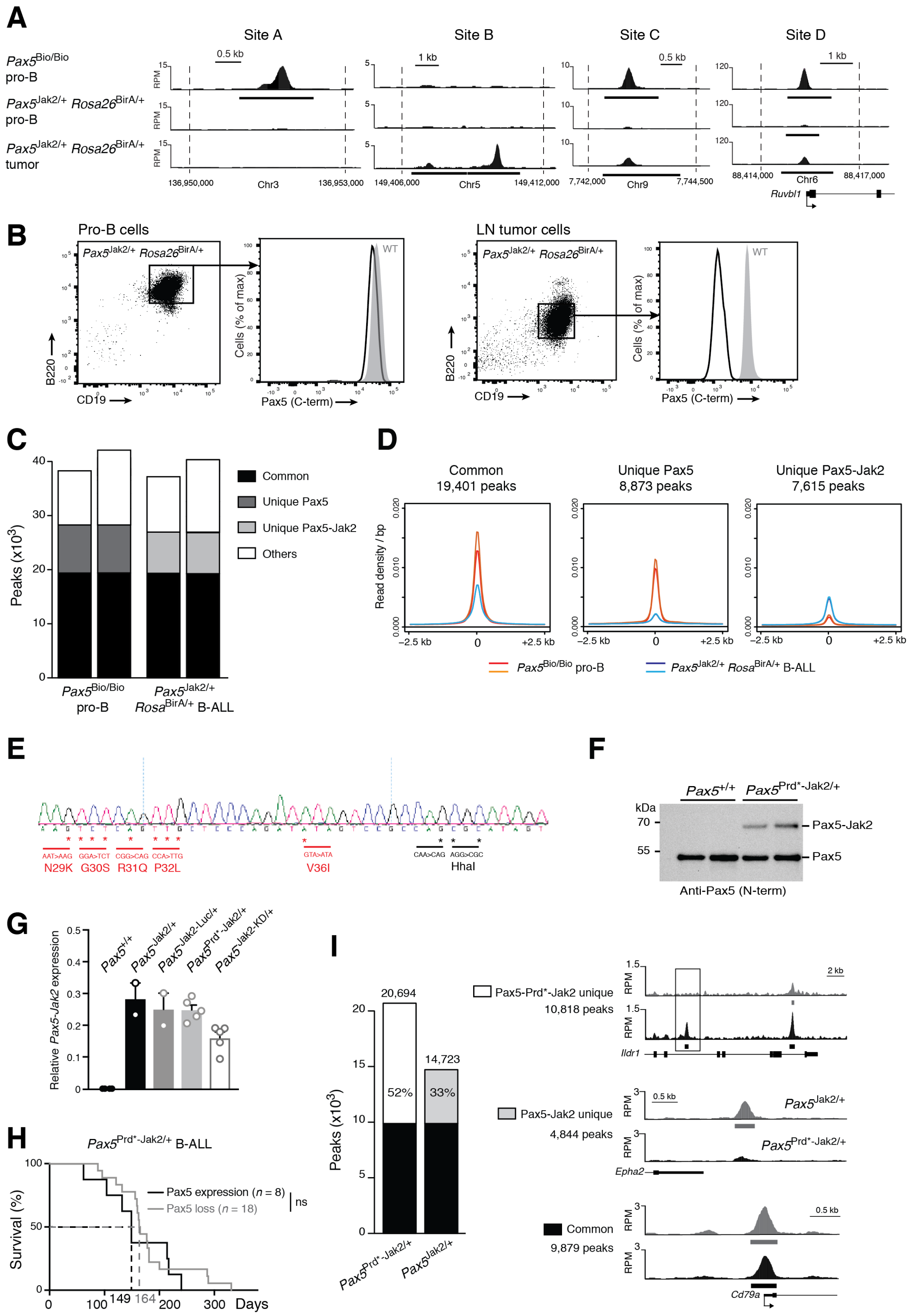

Appendix Figure S5. Leukemia formation depends on the DNA-binding function of Pax5-Jak2.

(A) Binding of Pax5 and Pax5-Jak2 at the selected sites A-D, as revealed by Bio-ChIP-seq analysis of *Pax5*$^{Bio/Bio}$ pro-B, *Pax5*$^{Jak2/+}$ *Rosa26*$^{BirA/+}$ pro-B and *Pax5*$^{Jak2/+}$ *Rosa26*$^{BirA/+}$ tumor cells. Horizontal bars indicate the Pax5 or Pax5-Jak2 peaks that were identified by MACS peak calling. The mm9 genomic coordinates of the four regions are indicated. **(B)** Flow-cytometric analysis and intracellular Pax5 staining of short-term cultured *Pax5*$^{Jak2/+}$ *Rosa26*$^{BirA/+}$ pro-B cells from a 3-week-old mouse and *ex vivo* B-ALL cells from the lymph node of a *Pax5*$^{Jak2/+}$ *Rosa26*$^{BirA/+}$ tumor mouse. These cells were used to prepare the chromatin templates for the Bio-ChIP-qPCR experiments shown in Fig 5D. *Pax5*$^{+/+}$ (WT) B cells were used as controls. **(C)** Common and unique peaks determined by two independent Bio-ChIP-seq experiments performed with *Pax5*$^{Bio/Bio}$ pro-B cells and *Pax5*$^{Jak2/+}$ *Rosa26*$^{BirA/+}$ B-ALL tumor cells (Fig 5B). Pax5 and Pax5-Jak2 peaks, identified by MACS peak calling with a *P* value of < 10^{-10}, were classified as common peaks (black), unique Pax5 peaks (dark grey) and unique Pax5-Jak2 peaks (light grey). 'Others' refers to several other combinations of the Pax5(-Jak2) peaks between the four Bio-ChIP-seq experiments. **(D)** Density of Pax5 and Pax5-Jak2 binding at common and unique peaks. Average read density profiles aligned at the center of the Pax5 or Pax5-Jak2 peaks are shown for a region extending from -2.5 kb to +2.5 kb. **(E)** Generation of the *Pax5*$^{Prd*\text{-}Jak2}$ allele by introducing the five amino acid changes N29K, G30S, R31Q, P32L and V36I into the *Pax5*$^{LSL\text{-}Jak2}$ allele by CRISPR/Cas9-mediated mutagenesis (see Appendix Supplementary Methods). The introduced mutations were verified by PCR amplification, cloning and Sanger sequencing of the respective genomic DNA fragment. In addition to the five amino acid substitutions (red), three silent mutations (grey) were introduced to prevent Cas9 cleavage in the zygote and to generate a HhaI restriction site (GCGC) for genotyping. **(F)** Expression of the Pax5Prd*-Jak2 and Pax5 proteins in *ex vivo* sorted B220^{+}CD19^{+} B cells from the bone marrow of 7-week-old *Pax5*$^{Prd*\text{-}Jak2\ /+}$ and control *Pax5*$^{+/+}$ mice, as shown by immunoblot analysis with an anti-Pax5 paired domain antibody. **(G)** RT-qPCR analysis of *Pax5-Jak2* mRNA expression in short-term cultured pro-B cells from 2-4-week-old *Pax5*$^{Jak2/+}$, *Pax5*$^{Jak2\text{-}Luc/+}$, *Pax5*$^{Prd*\text{-}Jak2\ /+}$ and *Pax5*$^{Jak2\text{-}KD/+}$ mice with primers unique for the *Pax5-Jak2* transcripts (primers in *Pax5* exon 5 and *Jak2* exon 19; Table EV1). The *Pax5-Jak2* transcripts were normalized to the *Tbp* mRNA. **(H)** Kaplan-Meier survival analysis of *Pax5*$^{Prd*\text{-}Jak2/+}$ mice that developed B-ALL tumors either expressing Pax5 (black) or lacking *Pax5* (grey). The differences between the two survival curves were non-significant (ns) as shown by the log-rank (Mantel-Cox) test. *n*, number of mice analyzed. **(I)** Pax5-Jak2 binding in *Pax5*$^{Prd*\text{-}Jak2\ /+}$ B-ALL cells, which lost the wild-type *Pax5* allele. *In vitro* cultured *Pax5*$^{Prd*\text{-}Jak2\ /+}$ and *Pax5*$^{Jak2/+}$ B-ALL cells were analyzed by ChIP-seq analysis with an antibody detecting the N-terminal paired domain of Pax5. Common and unique Pax5-Jak2 peaks were identified by stringent MACS peak calling with a *P* value of < 10^{-10} (left). Representative genes with common or unique Pax5-Jak2 peaks in the two B-ALL types are shown to the right.

Appendix Figure S6

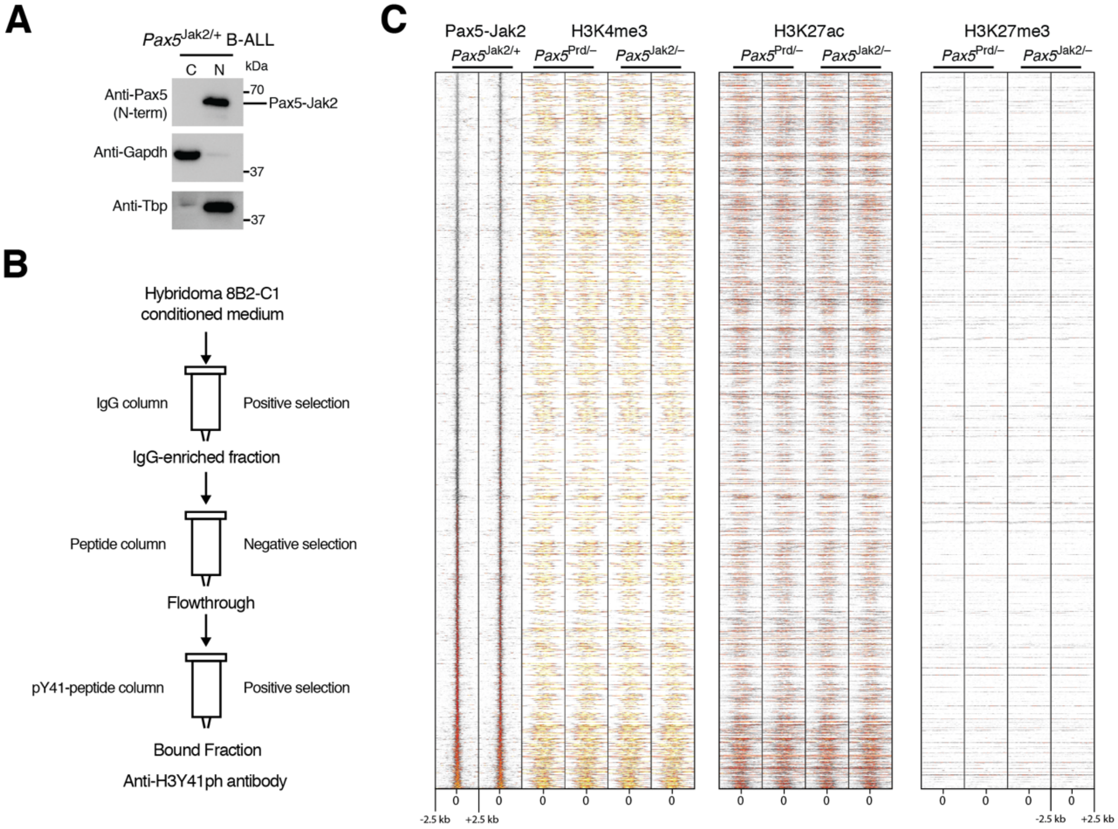

Appendix Figure S6. Pax5-Jak2 does not induce H3Y41 phosphorylation or active histone marks.

(A) Nuclear location of Pax5-Jak2. *In vitro* cultured *Pax5*$^{\text{Jak2/+}}$ B-ALL cells were separated into nuclear (N) and cytoplasmic (C) fractions, which were analyzed by immunoblotting for the presence of the nuclear Tbp, cytoplasmic Gapdh and Pax5-Jak2 proteins (see Appendix Supplementary Methods). **(B)** Schematic diagram explaining the purification of the anti-H3Y41ph antibody. Conditioned medium of 8B2-C1 hybridoma cells was enriched for IgG antibodies on an a Poros A column (Applied Biosystems). The eluate was passed through a column of Poros matrix coupled with the non-phosphorylated H3 peptide (Fig 6A) and the flowthrough fraction was bound to a column of Poros matrix coupled with the pY41-peptide followed by elution of the purified anti-H3Y41ph antibody. **(C)** Presence of H3K4me3 and H3K27ac and absence of H3K27me3 at Pax5-Jak2 peaks in *Pax5*$^{\text{Jak2/–}}$ and *Pax5*$^{\text{Prd/–}}$ progenitor cells. Density heat maps are shown for a region extending from -2.5 kb to +2.5 kb relative to the Pax5-Jak2 peak summit and were sorted according to the increasing density of Pax5-Jak2 binding, which was determined in *Pax5*$^{\text{Jak2/+}}$ tumor cells (Fig 5B).

Appendix Figure S7

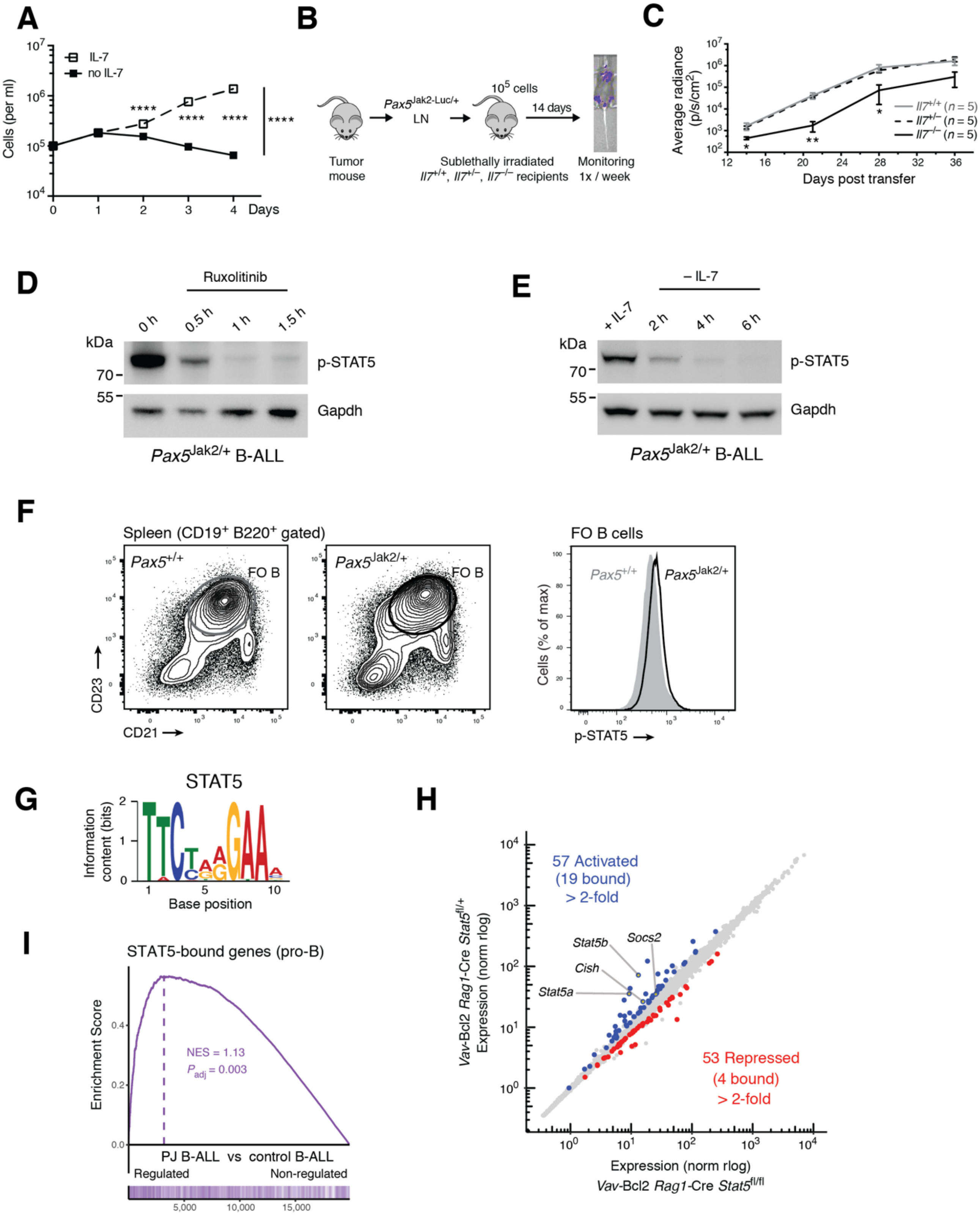

Appendix Figure S7. IL-7-dependent *Pax5*$^{\text{Jak2/+}}$ tumor growth and identification of STAT5-regulated genes in pro-B cells.

(A) IL-7-dependent proliferation of *Pax5*$^{\text{Jak2/+}}$ tumor cells. *In vitro* propagated *Pax5*$^{\text{Jak2/+}}$ B-ALL cells were seeded on ST2 feeder cells with or without IL-7, and the cell number per ml of culture medium was measured during the next 4 days. Statistical data are shown as mean value with SEM and were analyzed by two-way ANOVA with Šídák's multiple comparison test: ****$P < 0.0001$. **(B)** Schematic diagram describing the experimental outline for measuring the IL-7-dependent growth of *Pax5*$^{\text{Jak2-Luc/+}}$ tumor cells. B220^{low} B-ALL cells were sorted by flow cytometry from the lymph node of moribund *Pax5*$^{\text{Jak2-Luc/+}}$ mice, and 10^5 cells were intravenously transferred into sublethally irradiated *Il7*$^{-/-}$, *Il7*$^{+/-}$ or *Il7*$^{+/+}$ recipient mice. Tumor development was monitored once per week by *in vivo* bioluminescence measurement, starting at day 14 post transfer. **(C)** Second experiment analyzing the tumor progression in *Il7*$^{+/+}$ (grey), *Il7*$^{+/-}$ (dashed) and *Il7*$^{-/-}$ (black) mice transplanted with *Pax5*$^{\text{Jak2-Luc/+}}$ tumor cells, as determined by bioluminescence measurements at the indicated days after cell transfer. The bioluminescence measurements are shown as mean radiance values with SEM and were analyzed by the mixed-effect model REML with Geisser-Greenhouse's correction and Tukey's multiple comparison test: *$P < 0.05$, **$P < 0.01$. *n*, number of mice analyzed. **(D)** Loss of STAT5 phosphorylation upon ruxolitinib treatment. *Pax5*$^{\text{Jak2/+}}$ B-ALL cells were *in vitro* cultured on ST2 feeder cells in the presence of IL-7 and were then treated with vehicle (0 h) or 10 μM ruxolitinib for 0.5, 1 and 1.5 h. Whole-cell extracts of untreated or treated *Pax5*$^{\text{Jak2/+}}$ B-ALL cells were analyzed by immunoblot analysis with anti-p-STAT5 and anti-Gapdh antibodies. The size of marker proteins is indicated in kilodaltons (kDa) to the left. **(E)** Loss of STAT5 phosphorylation upon IL-7 withdrawal. Whole-cell extracts of *Pax5*$^{\text{Jak2/+}}$ B-ALL cells, which were *in vitro* cultured in the presence of IL-7 or after IL-7 withdrawal for 2, 4 or 6 h, were analyzed by immunoblot analysis with anti-p-STAT5 and anti-Gapdh antibodies. **(F)** STAT5 phosphorylation in B220$^+$ follicular (FO) B cells (B220$^+$CD19$^+$CD21^{lo}CD23^{hi}) from the spleen of 4-week-old *Pax5*$^{\text{Jak2/+}}$ (black) and 8-week-old *Pax5*$^{+/+}$ (grey) mice. **(G)** Consensus STAT5-binding motif identified with an E-value of 4.4×10^{-218} by the *de novo* motif discovery program MEME-ChIP. **(H)** Scatter plot of gene expression differences between *Vav-Bcl2 Rag1*-Cre *Stat5*$^{\text{fl/fl}}$ and control *Vav-Bcl2 Rag1*-Cre *Stat5*$^{\text{fl/+}}$ pro-B cells, which were *ex vivo* sorted from the bone marrow prior to RNA-seq analysis. The expression data of individual genes (indicated by dots) are plotted as normalized rlog (regularized logarithm) values. Genes with an expression difference of > 2-fold, an adjusted *P* value of < 0.05 and a TPM value of > 5 (in at least one pro-B cell type) are colored in blue and red, corresponding to activation or repression by STAT5, respectively (Dataset EV4). STAT5-bound genes are indicated, based on the genome-wide analysis of STAT5 binding by ChIP-seq analysis of *in vitro* cultured wild-type pro-B cells (see Methods). **(I)** GSEA analysis of the 1,606 STAT5-bound genes identified in pro-B cells, as compared to the ranked $\log_2$-fold gene expression changes in *Pax5*$^{\text{Jak2/+}}$ (PJ) B-ALLs versus control *Pax5*$^{+/-}$ *Cdkn2ab*$^{+/-}$ B-ALLs (see Methods). NES, normalized enrichment score.

2. Appendix Supplementary Methods

Mice

The following mice were maintained on the C57BL/6 genetic background: *Pax5*$^{+/-}$ (Urbánek et al., 1994), *Pax5*$^{ihCd2/ihCd2}$ (Fuxa and Busslinger, 2007), *Pax5*$^{Bio/Bio}$ (McManus et al., 2011), *Pax5*$^{Prd/+}$ (Smeenk et al., 2017), *Pax5*$^{Etv6/+}$ (Smeenk et al., 2017), *Cdkn2ab*$^{+/-}$ (Krimpenfort et al., 2007), *Il7*$^{+/-}$ (von Freeden-Jeffry et al., 1995), *Rosa26*$^{BirA/BirA}$ (Driegen et al., 2005), *Ikzf1*$^{neo/+}$ (Souabni et al., 2002), *Stat5*$^{fl/fl}$ (Cui et al., 2004), *Cd79a*(Mb1)$^{Cre/+}$ (Hobeika et al., 2006), *Meox2*$^{Cre/+}$ (Tallquist and Soriano, 2000), *Rag1*$^{Cre/+}$ (McCormack et al., 2003), transgenic FLPe (Rodriguez et al., 2000), transgenic CAGGs-Dre (Anastassiadis et al., 2009) and transgenic *Vav*-Bcl2 (Ogilvy et al., 1999). In this manuscript, we refer to the heterozygous *Meox2*$^{Cre/+}$, *Rag1*$^{Cre/+}$ and *Cd79a*$^{Cre/+}$ genotypes as *Meox2*-Cre, *Rag1*-Cre and *Cd79a*-Cre, respectively. All animal experiments were carried out according to valid project licenses, which were approved and regularly controlled by the Austrian Veterinary Authorities. Mice were monitored for indication of disease and were sacrificed when they showed signs of terminal illness.

Generation of *Pax5*$^{Jak2/+}$, *Pax5*$^{Jak2\text{-}Luc/+}$, *Pax5*$^{Prd*\text{-}Jak2/+}$ and *Pax5*$^{Jak2\text{-}KD/+}$ mice

The *Pax5*$^{Jak2\text{-}Luc}$ and *Pax5*^{Jak2} alleles were generated by cloning of the targeting vector shown in Appendix Fig S1B and described in detail in the corresponding figure legend. Targeting was performed in the ES cell line A9. PCR-positive clones were verified by Southern blot analysis (Appendix Fig S1C) before injection into C57BL/6 blastocysts and the generation of *Pax5*$^{LSL\text{-}Jak2\text{-}Luc\text{-}Neo/+}$ mice. *Pax5*$^{LSL\text{-}Jak2\text{-}Luc/+}$ and *Pax5*$^{LSL\text{-}Jak2\ /+}$ mice were obtained by sequential deletion of the neomycin (*neo*^{r}) resistance gene and IRES-luciferase gene by crossing *Pax5*$^{LSL\text{-}Jak2\text{-}Luc\text{-}Neo/+}$ mice with the transgenic FLPe and CAGGs-Dre lines, respectively (Appendix Fig S1B). The *loxP*-stop-*loxP* (LSL) cassette was deleted by crossing of *Pax5*$^{LSL\text{-}Jak2\text{-}Luc/+}$ and *Pax5*$^{LSL\text{-}Jak2\ /+}$ mice with the *Meox2*-Cre line to generate the *Pax5*$^{Jak2\text{-}Luc/+}$ and *Pax5*$^{Jak2\ /+}$ mice. The following primers shown in Fig 3E were used for PCR genotyping of the *Pax5*$^{LSL\text{-}Jak2\text{-}Luc/+}$, *Pax5*$^{Jak2\text{-}Luc/+}$, *Pax5*$^{LSL\text{-}Jak2\ /+}$ *Pax5*$^{Jak2\ /+}$ mice: (a) 5'-GGTTCTGAGGGTCCAGGAAT-3', (b) 5'-AGGTTCAGCCCTTG GAGAAT-3' and (c) 5'-CGAGAACTTGTTTATTGCAGCTT-3'. The different *Pax5*^{Jak2} alleles were identified by amplifying a 320-bp PCR fragment with primer pair b/c, and the wild-type *Pax5* allele by amplifying a 744-bp PCR fragment with primer pair a/b.

Pax5$^{LSL\text{-}Jak2\text{-}KD/+}$ and *Pax5*$^{LSL\text{-}Prd*\text{-}Jak2/+}$ mice were generated by CRISPR/Cas9-mediated genome editing in mouse *Pax5*$^{LSL\text{-}Jak2/+}$ zygotes on a C57BL/6 or C57BL/6 x CBA background (Yang et al., 2013). Mouse zygotes were injected with Cas9 mRNA, a sgRNA specific for the sequence to be mutated (linked to the scaffold tracrRNA) and a single-stranded DNA repair template of 200 nucleotides to introduce the specific mutations in the kinase or paired domain of the *Pax5*$^{LSL\text{-}Jak2\text{-}KD}$ (Appendix Fig S4A) or *Pax5*$^{LSL\text{-}Prd*\text{-}Jak2}$ (Appendix Fig S5E) allele, respectively. Mice carrying the introduced mutation were identified by amplification of the respective PCR fragment

(Table EV1) and subsequent restriction digestion with AgeI (KD [K272E], Appendix Fig S4A) or HhaI (Prd*, Appendix Fig S5E). After sequence verification, the mutant *Pax5*$^{\text{Jak2}}$ allele was PCR-genotyped with primer pair a/b, as indicated above. Mice on a mixed background were backcrossed to the C57BL/6 background, and *Pax5*$^{\text{LSL-Prd*-Jak2/+}}$ and *Pax5*$^{\text{LSL-Jak2-KD/+}}$ mice were crossed with the *Meox2*-Cre line to obtain *Pax5*$^{\text{Prd*-Jak2/+}}$ and *Pax5*$^{\text{Jak2-KD/+}}$ mice.

Antibodies

The following monoclonal antibodies were purchased from BD Biosciences, Thermo Fisher Scientific, BioLegend or Miltenyi Biotec and were used for flow-cytometric analysis of the mouse spleen, lymph node and bone marrow: B220/CD45R (RA3-6B2), CD2 (RM2-5), CD11b/Mac1 (M1/70), CD19 (1D3), CD21/CD35 (7G6), CD23 (B3B4), CD25/IL-2Rα (PC61), CD93/AA4.1 (AA4.1), CD117/c-Kit (2B8), CD127/IL-7Rα (A7R34), CD135/Flt3 (A2F10), Gr1 (RB6-8C5), IgD (11.26c), Igκ (187.1), IgM (II/41 or eB121-15F9), Gr1 (RB6-8C5) and TCRβ (H57-597).

The rabbit monoclonal anti-Pax5 antibody (C-terminal; D19F8; Cell Signaling Technology) and mouse monoclonal anti-p-STAT5 antibody (47/Stat5 pY694; BD Biosciences) were used for the flow-cytometric detection of Pax5 and p-STAT5 by intracellular staining using the Foxp3/transcription factor staining buffer set (eBioscience; 00-5523-00) according to the manufacturer's instructions. The following antibodies were used for immunoblot analysis: rabbit polyclonal anti-Pax5 (directed against amino acids 17-145; Adams et al., 1992), rabbit monoclonal anti-p-STAT5 (C11C5 pY694; Cell Signaling Technology), mouse monoclonal anti-TBP (3TF1-3G3; Active Motif), rabbit monoclonal anti-H3, HRP-coupled (D1H2; Cell Signaling Technology) and rabbit monoclonal anti-GAPDH, HRP-coupled (14C10; Cell Signaling Technology). ChIP analysis was performed with the following antibodies recognizing STAT5 or different histone tail modifications: rabbit polyclonal anti-STAT5A and anti-STAT5B (PA-ST5A and PA-ST5B, R&D Systems), rabbit polyclonal anti-H3K4me3 (pAb-003-050; Diagenode), rabbit polyclonal anti-H3K27ac (ab4729, Abcam), rabbit monoclonal anti-H3K27me3 (C36B11; Cell Signaling Technology) antibody.

Generation of a monoclonal H3Y41ph-specific antibody

Fifty mg of the phosphorylated H3 peptide (amino acids 37-46 with phosphorylated (p) Y41; Fig 6A) in complete Freund's adjuvant was subcutaneously injected into one mouse. After three immunizations, 30 mg of the phosphorylated H3 peptide (without adjuvant) was injected as final boost into the tail vein. Four days after the final immunization, the splenocytes were fused with the myeloma cell line X63-Ag8.653 according to standard protocols using polyethylene glycol (Harlow E., Lane D. (1988) Antibodies: a laboratory manual. Cold Spring Harbor: Cold Spring Harbor Laboratory). Cells were seeded into 96-well plates, and fused hybridoma cells were selected in HAT-containing growth medium. Seven days after fusion, hybridoma supernatants were screened for the presence of a H3Y41ph-specific antibody by ELISA against an unmodified or pY41-

modified H3 peptides (amino acids 29-53, Fig 6A). Single clones secreting a H3Y41ph-specific antibody were obtained by limited dilution of the hybridoma cell mixture of positive wells.

ELISA measurements were performed by coating ELISA plates with 3 mg/ml of the unmodified or phosphorylated H3 peptide (amino acids 29-53). After washing with TBS and blocking with 2% BSA in TBS, the wells were incubated with hybridoma supernatant at 3-fold serial dilutions for 2 h at room temperature. After washing, a secondary anti-mouse IgG antibody coupled to horseradish peroxidase (115-035-008; Jackson ImmunoResearch) was added for 1 h at room temperature. After a final washing, the substrate 3,3´,5,5´-tetramethylbenzidine (T2885; Sigma) was added to initiate the enzymatic reaction, which was stopped by addition of 0.5 M H_2SO_4 followed by measuring the absorption at 450 nm.

Flow cytometric sorting and definition of mouse hematopoietic cell types

Cell types were defined as follows: Pax5-deficient progenitors ($CD19^{-}B220^{+}Kit^{+}Ly6D^{+}$), pro-B cells ($CD19^{+}B220^{+}Kit^{+}CD2^{-}IgM^{-}IgD^{-}$), pre-B cells ($CD19^{+}B220^{+}Kit^{-}CD2^{+}IgM^{-}IgD^{-}$), large pre-B cells ($CD19^{+}B220^{+}Kit^{-}IgM^{-}IgD^{-}FSC^{hi}$), small pre-B cells ($CD19^{+}B220^{+}Kit^{-}IgM^{-}IgD^{-}FSC^{lo}$), immature B cells ($CD19^{+}B220^{+}IgM^{+}IgD^{-}$), recirculating bone marrow B cells ($CD19^{+}B220^{+}IgD^{+}$), mature splenic B cells ($CD19^{+}B220^{+}IgD^{+}$), follicular (FO) B cells ($CD19^{+}B220^{+}CD23^{+}CD21^{lo}$), marginal zone (MZ) B cells ($CD19^{+}B220^{+}CD21^{hi}CD23^{lo}$), T cells ($TCR\beta^{+}$), myeloid cells ($CD11b^{+}Gr1^{+}$). The different hematopoietic cell types were identified in bone marrow, spleen and lymph nodes using an LSR Fortessa (BD Biosciences). Cells were sorted with a FACSAria III (BD Biosciences) machine. Flowjo software (Treestar) was used for data analysis.

Transplantation experiments and ruxolitinib treatment

Tumor cells from lymph nodes of moribund *Pax5*$^{Jak2/+}$ and *Pax5*$^{Jak2\text{-}Luc/+}$ mice as well as $B220^{low}$ and $B220^{+}$ B cells from the bone marrow of *Pax5*$^{Jak2/+}$ mice were sorted by flow cytometry and transferred by intravenous injection (10^5 cells per mouse) into sublethally irradiated C57BL/6 mice (4.5 Gy). Tumor development was monitored, and mice were sacrificed, when showing signs of terminal illness. Where applicable, ruxolitinib (R-6688; LC Laboratories) was administered twice daily by oral gavage of 45 mg/kg in 0.5% methycellulose (M0512; Sigma).

Tumor monitoring by bioluminescence measurements

For bioluminescence imaging, mice were intraperitoneally injected with D-luciferin (150 mg/kg, Goldbio) and imaged with an IVIS Spectrum Xenogen machine (Caliper Life Sciences).

Histopathology

For histopathological analyses, organs were isolated and fixed in 4% paraformaldehyde, dehydrated and processed with the Logos Tissue processor. Processed organs were embedded in paraffin, sectioned at a thickness of 2 μm and stained with hematoxylin and eosin (H&E). Sections were

scanned using a Mirax slide scanner. Stained slides were reviewed by a board-certified pathologist with a Zeiss Axioskop 2 MOT microscope (Carl Zeiss Microscopy).

Patient samples

Total RNA from PAX5-JAK2^{+} B-ALL samples was prepared for subsequent RNA-seq analysis. The human RNA-seq data are available upon request.

***In vitro* culture of pro-B cells and B-ALL cell lines**

Pro-B cells were cultured on OP9 feeder cells in IL7-containing IMDM as described (Nutt et al., 1997). B-ALL cells were isolated from lymph nodes of *Pax5*$^{Jak2/+}$ *Rosa26*$^{BirA/+}$ or *Pax5*$^{Etv6/+}$ *Cdkn2ab*$^{+/-}$ tumor mice and were established as cell lines by culturing them on ST2 feeder cells in IMDM medium containing IL-7 and heat-inactivated 10% fetal calf serum.

Protein extract preparation and immunoblot analysis

Nuclear extracts were prepared from short-term cultured pro-B cells as described in detail (Minnich et al., 2016). Whole-cell extracts were prepared using the Cell Lysis buffer (9803; Cell Signaling Technology), containing multiple phosphatase inhibitors, according to the manufacturer's instructions. Subcellular fractionation of B-ALL cells was performed with the Cell Fractionation kit (ab109719; Abcam). The proteins of whole-cell, cytoplasmic or nuclear extracts were denatured in 3x SDS-PAGE sample buffer containing β-mercaptoethanol, boiled, separated by SDS-PAGE and analyzed by immunoblot analysis.

qPCR determination of the copy number of the different *Pax5* alleles

B-ALL cells (CD19$^{+/-}$B220^{low}) from *Pax5*$^{Jak2/+}$ tumor mice, CD19^{+}B220^{+} and pre-leukemic CD19^{+}B220^{low} B cells from the bone marrow of 6-week-old *Pax5*$^{Jak2/+}$ mice and CD19^{+}B220^{+} B cells from *Pax5*$^{+/+}$ bone marrow were isolated by flow cytometry, and genomic DNA was extracted using the DNeasy Blood & Tissue kit (Qiagen) according to the manufacturer's instruction, including RNAse treatment. qPCR analysis was performed with the Luna® Universal qPCR Master Mix (NEB) using 20 ng of genomic DNA and primers specific for the *Pax5*^{Jak2} or *Pax5*$^{+}$ allele (Table EV1). The PCR data were normalized to the product amplified from a control region in intron 2 of the *Car8* gene located 35 Mb upstream of *Pax5*.

RT-qPCR analysis of mRNA expression

Total RNA was prepared from short-term cultured pro-B cells using a semi-automated RNA bead isolation method with Sera-Mag SpeedBead Carboxylate-Modified Magnetic Particles (Hydrophobic; GE Healthcare) run on the magnetic particle processor KingFisher Duo instrument (Thermo Fisher Scientific). The cDNA was synthesized using Oligo d(T)$_{18}$ primer (NEB) and SuperScript II Reverse Transcriptase (Thermo Fisher Scientific) in the presence of RNase inhibitor

(Thermo Fisher Scientific). The transcripts of selected genes were amplified by qPCR using primers located in different exons (Table EV1) and were normalized against the *Tbp* mRNA.

ChIP analysis of Pax5-Jak2 and STAT5 binding as well as chromatin modifications

Ex vivo B-ALL cells from *Pax5*$^{Jak2/+}$ *Rosa26*$^{BirA/+}$ tumor mice, *ex vivo* sorted *Pax5*$^{Bio/Bio}$ pro-B cells, short-term cultured *Pax5*$^{Jak2/+}$ *Rosa26*$^{BirA/+}$ pro-B cells as well as short-term cultured *Pax5*$^{Jak2/–}$ and *Pax5*$^{Prd/–}$ progenitor cells were crosslinked at room temperature with 1% formaldehyde (Sigma) for 10 min, before the nuclei were prepared and lysed in the presence of 0.25% SDS (Pax5[-Jak2] Bio-ChIP) or 1% SDS (histone modification and STAT5 ChIP). The chromatin was sheared by sonication with the Bioruptor® Standard (Diagenode), followed by immunoprecipitation with specific antibodies (for description see 'Antibodies') or by streptavidin-mediated pull-down of *in vivo* biotinylated Pax5 and Pax5-Jak2 proteins, as previously described (Ebert et al., 2011; Revilla-i-Domingo et al., 2012). The precipitated DNA at selected genomic sites was determined by qPCR analysis with specific primers (Table EV1), and the relative enrichment was calculated as percent of precipitated DNA relative to the input DNA. For analysis of STAT5 binding, wild-type pro-B cells were cultured on OP9 cells in the presence of IL-7, before IL-7 was withdrawn for 4 h and the pro-B cells were restimulated with IL-7 (10 ng/ml) for 30 min prior to formaldehyde fixation, nuclei preparation and ChIP analysis with a mixture of anti-STAT5A and anti-STAT5B antibodies. For ChIP-seq analysis, 0.5-5 ng of ChIP-precipitated DNA was used for library preparation and subsequent Illumina deep sequencing.

Complementary DNA (cDNA) preparation for RNA-sequencing

RNA from *ex vivo* sorted pro-B cells and B-ALL cells was isolated with the RNeasy Plus Mini Kit (Qiagen). mRNA was obtained by two rounds of poly(A) selection using the Dynabeads mRNA purification kit (Invitrogen) and fragmented by heating at 94 °C for 3 min in fragmentation buffer. The fragmented mRNA was used as template for first-strand cDNA synthesis with random hexamers using the Superscript Vilo First-Strand Synthesis System (Invitrogen). The second-strand cDNA was synthesized with 100 mM dATP, dCTP, dGTP and dUTP in the presence of RNase H, *E. coli* DNA polymerase I and DNA ligase (Invitrogen).

Library preparation and Illumina deep sequencing

About 0.5-5 ng of cDNA or ChIP-precipitated DNA were used as starting material for the generation of sequencing libraries with the NEBNext Ultra II DNA library prep kit for Illumina (NEB). Alternatively, sequencing libraries were generated using the NEBNext End Repair/dA-Tailing Module and NEBNext Ultra Ligation Module (NEB) followed by amplification with the KAPA Real-Time Amplification kit (KAPA Biosystems). Cluster generation and sequencing was carried out by using the Illumina HiSeq 2000/2500 system with a read length of 50 nucleotides (single-

read) or 100/125 nucleotides (paired-end) according to the manufacturer's guidelines. Table EV2 provides further information about all sequencing experiments of this study.

ChIP-seq read processing

After sequencing, all reads that passed the Illumina quality filtering were considered for alignment.

ChIP-seq analysis (mm10)

The ChIP-seq samples, which were used for generating the data shown in Fig 5B and Appendix Fig S5C, D and I, were aligned to the mouse genome assembly version of December 2011 (GRCm38/mm10) using the bwa program version 0.7.17 (Li and Durbin, 2009). Where necessary (e.g. for comparisons after peak calling), reads were down-sampled to the lowest read number in the samples to be compared, using the Picard Toolkit version 2.18.27 (http://broadinstitute.github.io/picard/). The read coverages, displayed in Fig 5B and Appendix Fig S5I, were calculated with the bedtools program version 2.27.1 (Quinlan and Hall, 2010), normalized to reads per millions (RPM) using the SAMtools version 1.9 (Li et al., 2009) and Kent tools version 20190507 (Kuhn et al., 2013) and were visualized with the UCSC genome browser (Kuhn et al., 2013). Peaks were called with the MACS program version 2.2.5 (Zhang et al., 2008), using only one of the two read sequences in case of paired-end read samples, with the input sample 35327 (*ex vivo Rag2*$^{\text{Cre/Cre}}$ pro-B cells) as a control, except for the B-ALL samples 138002 and 138003, where the associated input sample 138001 was used (Table EV2). All peaks were filtered for significance (*P* value < 10^{-10}) and subsequently assigned to genes as described (Revilla-i-Domingo et al., 2012), based on the RefSeq database that was downloaded from UCSC on December 6th, 2018. Peak overlap analysis was done with the MULTOVL program version 1.2 (Aszódi, 2012) and read densities were calculated using the JNOMICS program (I. Tamir, unpublished). Associated heat map visualizations were implemented using R (http://www.R-project.org) and were wrapped with customized bash scripts for command line usage. *De novo* motifs were predicted by using the MEME-ChIP suite version 5.0.4 (Bailey et al., 2015) to analyze a 300-bp regions centered at the summit of the top 300 peaks of each ChIP-seq sample.

ChIP-seq analysis (mm9)

The ChIP-seq samples, which were used for generating the data, shown in Fig 7G, H and K, Appendix Fig S2A, Appendix Fig S5A and Appendix Fig S7G, H and I, were aligned to the mouse genome assembly version of July 2007 (NCBI37/mm9), using the Bowtie program version 1.0.0 (Langmead et al., 2009). Peaks were called with the MACS program version 1.3.6.1 (Zhang et al., 2008) and subsequently filtered for significance (*P* value < 10^{-10}).

Peaks of samples 29936 and 29938 (*ex vivo Pax5*$^{\text{Bio/Bio}}$ pro-B cells), 38870 and 40031 (*in vitro Pax5*$^{\text{Jak2/+}}$ *Rosa26*$^{\text{BirA/+}}$ pro-B cells) and 40030 and 46051 (*ex vivo Pax5*$^{\text{Jak2/+}}$ *Rosa26*$^{\text{BirA/+}}$ B-ALL cells, Table EV2) were called by using the pro-B cell input GSM2461724 as control. The resulting

peaks were overlapped using the MULTOVL version 1.2 (Aszódi, 2012) to identify the sites A, B, C and D (Appendix Fig S5A), which display distinct binding patterns between the three different cell types analyzed.

Definition of Pax5 and STAT5 target genes in pro-B cells (mm9)

All filtered peaks were assigned to genes as described (Revilla-i-Domingo et al., 2012), based on the RefSeq datbase, which was downloaded from UCSC on January 10th, 2014. The overlapping set between bound and regulated genes, as defined by RNA-seq (see RNA-seq analysis mm9), was defined as regulated target gene set.

For defining Pax5 target genes, the Pax5 ChIP-seq samples 29936 and 29938 (*ex vivo* *Pax5*$^{Bio/Bio}$ pro-B cells), 35059 and 35061 (*ex vivo* wild-type pro-B cells) and the input 35327 (*ex vivo Rag2*$^{Cre/Cre}$ pro-B cells, Table EV2) were down-sampled to 20 million reads by using the UNIX bash command "shuf". Peaks were called with the MACS program version 2.2.5 (Zhang et al., 2008), using the input 35327 as control and only one of the two read sequences in case of paired-end read samples. A gene was considered to be bound if at least one peak in one of the four analyzed ChIP-seq samples was assigned to it.

For defining STAT5 target genes, peaks were called using the STAT5 ChIP-seq sample 8265 (*in vitro* wild-type pro-B cells) with the pro-B cell input GSM2461724 as control. Significant enrichment of STAT5 binding in regulated genes (Fig S7I), identified in *Pax5*$^{Jak2/+}$ B-ALL cells (Fig 2C), was calculated by applying a chi-square test with Yates' continuity correction, using all regulated genes with a TPM value > 5 and a fold-change > 3, excluding histone and immunoglobulin genes. Non-regulated genes were defined by a fold-change between > -1.25 and < +1.25.

All steps of the ChIP-seq analysis were implemented as customized UNIX bash (http://www.R-project.org) scripts.

RNA-seq read processing

After sequencing, reads that passed the Illumina quality filtering were considered for alignment. Reads corresponding to mouse ribosomal RNAs (BK000964.1 and NR046144.1) or human ribosomal RNAs (U13369.1) were removed.

RNA-seq differential expression analysis (mm9)

The RNA-seq samples, which were used to define the gene expression changes between *ex vivo* sorted *Pax5*$^{-/-}$ and *Pax5*$^{+/+}$ pro-B cells (Appendix Fig S2A) as well as *ex vivo* sorted pro-B cells from *Vav-Bcl2 Rag1*-Cre *Stat5*$^{fl/fl}$ and control *Vav-Bcl2 Rag1*-Cre *Stat5*$^{fl/+}$ (Appendix Fig S7H), were aligned to the mouse genome assembly version of July 2007 (NCBI37/mm9) using the TopHat program version 1.4.1a (Trapnell et al., 2009). The number of reads per gene was counted using featureCounts version 1.5.0 (Liao et al., 2014) with the default settings and the modified refGene gene annotation described (Schwickert et al., 2019). TPM values were calculated as described

(Wagner et al., 2012). The R package DESeq2 version 2_1.24 (Love et al., 2014) was used with default settings for sample normalizations and dispersion estimations. Regularized log transformations (rlog) were computed with the blind option set to 'FALSE'. Rlog counts were transformed from log_2 to log_{10} scale for all scatterplots. The default DESeq2 setup (model design formula: "~ genotype"; Wald test) was used for calling differentially expressed genes. Genes with an adjusted *P* value < 0.05 and an absolute fold-change > 3 (Appendix Fig S2A) or > 2 (Appendix Fig S7H) as well as a mean TPM (averaged within conditions) > 5 were called as significantly expressed. Immunoglobulin and T cell receptor genes were filtered from the list of significantly expressed genes but were included in the TPM (transcripts per million) calculations.

RNA-seq differential expression analysis (mm10)

The RNA-seq samples, which were used to define the gene expression changes between *Pax5*$^{Jak2/+}$ and *Pax5*$^{+/+}$ pro-B cells (Fig 2A) as well as *Pax5*$^{Jak2/+}$ and control *Pax5*$^{+/-}$ *Cdkn2ab*$^{+/-}$ B-ALL tumors (Fig 2C), were aligned to the mouse genome assembly version of December 2011 (GRCm38/mm10) using the STAR program version 2.4.2a (Dobin et al., 2013) with the parameters '–outSAMStrandField intronMotif' and '–removeNonCannonical RemoveNonCannonicalUnannot ated' in paired-end mode. The number of reads per gene was counted using featureCounts version 1.5.0 (Liao et al., 2014) with the setting -p and the refGene gene annotation of mm10, which was downloaded from UCSC on Dec 6th 2018 and expanded by immunoglobulin and T cell receptor gene annotation from Gencode version 29. TPM values were calculated as described (Wagner et al., 2012). The R package DESeq2 version 2_1.24 (Love et al., 2014) was used with default settings for sample normalizations and dispersion estimations. Regularized log transformations (rlog) were computed with the blind option set to 'FALSE'. Rlog counts were transformed from log_2 to log_{10} scale for all scatterplots. The default DESeq2 setup (model design formula: "~ genotype"; Wald test) was used for calling differentially expressed genes.

Genes with an adjusted *P* value < 0.05 and an absolute fold-change > 3 (Fig 2C) or > 2 (Fig 2A) as well as a mean TPM (averaged within conditions) > 5 were called as significantly expressed. Immunoglobulin and T cell receptor genes were filtered from the list of significantly expressed genes but were included in the TPM (transcripts per million) calculations.

RNA-seq principal component analysis (mm10)

RNA-seq samples, which were used for the generation of Appendix Fig S2E, were aligned to the mouse genome assembly version of December 2011 (GRCm38/mm10) using the STAR program version 2.4.2a (Dobin et al., 2013) in single-read mode. To mimic the characteristics of the wild-type samples, only the first read of the B-ALL paired-end data was considered for alignment and cut down to 50 bp prior to alignment. The number of reads per gene was counted using featureCounts version 1.5.0 (Liao et al., 2014) without setting the parameter -p and the refGene gene annotation of mm10, which was downloaded from UCSC on Dec 6th 2018 and expanded by the

immunoglobulin and T cell receptor gene annotation from Gencode version 29. Regularized log transformations (rlog) were computed by the R package DESeq2 version 2_1.24 (Love et al., 2014) with the blind option set to 'TRUE'. Principal component analysis was conducted based on the 500 most variable rlog values.

RNA-seq differential expression analysis (hg19)

Human RNA-seq samples were aligned to the human genome assembly version of February 2009 (GRCh37/hg19), using the STAR program version 2.4.2a (Dobin et al., 2013) with the parameters '–outSAMStrandField intronMotif' and '–removeNonCannonical RemoveNonCannonicalUnannotated' in paired-end mode. The number of reads per gene was counted using featureCounts version 1.5.0 (Liao et al., 2014) with the setting -p and the refGene gene annotation of hg19, which was downloaded from UCSC on Dec 6th 2018 and expanded by the immunoglobulin and T cell receptor gene annotation from Gencode version 29. TPM values were calculated as described (Wagner et al., 2012). The R package DESeq2 version 2_1.24 (Love et al., 2014) was used with default settings for sample normalizations and dispersion estimations. Regularized log transformations (rlog) were computed with the blind option set to 'FALSE'.

Fusion gene detection

Two custom genome references were created by adding the exonic human PAX5-JAK2 fusion sequence and the wild-type *Pax5* or *PAX5* exon 5-exon 6 sequence to the mouse genome assembly version of December 2011 (GRCm38/mm10) or human genome assembly version of February 2009 (GRCh37/hg19), respectively. B-ALL sample reads were aligned to their corresponding custom genomes with the STAR program version 2.4.2a (Dobin et al., 2013) in paired-end mode. The number of reads covering the specific *PAX5-JAK2* junction (*PAX5* exon 5-*JAK2* exon 19) versus the wild-type *Pax5* or *PAX5* exon 5-exon 6 junction were reported (Fig. S3C).

Gene set enrichment analysis

The R package apeglm version 1.8.0 (Zhu et al., 2019) was used to shrink $\log_2$-fold changes for gene ranking. Gene set enrichment was conducted with the R package clusterProfiler version 3.14.3 (Yu et al., 2012).

Statistical analysis

Statistical analysis was performed with the GraphPad Prism 8 software. Two-tailed unpaired Student's *t*-test analysis and multiple *t*-tests (unpaired and two-tailed with Holm-Šídák's correction) were used to assess the statistical significance of one observed parameter between two experimental groups. For comparison of multiple groups and analysis of repeated measurements, the ANOVA analysis or mixed model effect analysis (REML) were used together with post-hoc multiple

comparison tests (Šídák or Tukey, for two or more groups, respectively). The statistical evaluation of the RNA-seq data is described under 'Analysis of RNA-seq data'.

Data availability

The RNA-seq and ChIP-seq data generated for this study (Table EV2) are available at the Gene Expression Omnibus (GEO) repository under the accession number GSE174775. The human RNA-seq data are available upon request.

3. Appendix Supplementary Methods

Adams, B., P. Dörfler, A. Aguzzi, Z. Kozmik, P. Urbánek, I. Maurer-Fogy, and M. Busslinger. 1992. *Pax-5* encodes the transcription factor BSAP and is expressed in B lymphocytes, the developing CNS, and adult testis. *Genes Dev*. 6:1589-1607.

Anastassiadis, K., J. Fu, C. Patsch, S. Hu, S. Weidlich, K. Duerschke, F. Buchholz, F. Edenhofer, and A.F. Stewart. 2009. Dre recombinase, like Cre, is a highly efficient site-specific recombinase in *E. coli*, mammalian cells and mice. *Dis. Model. Mech*. 2:508-515.

Aszódi, A. 2012. MULTOVL: fast multiple overlaps of genomic regions. *Bioinformatics* 28:3318-3319.

Bailey, T.L., J. Johnson, C.E. Grant, and W.S. Noble. 2015. The MEME Suite. *Nucleic Acids Res*. 43:W39-W49.

Cui, Y., G. Riedlinger, K. Miyoshi, W. Tang, C. Li, C.-X. Deng, G.W. Robinson, and L. Hennighausen. 2004. Inactivation of Stat5 in mouse mammary epithelium during pregnancy reveals distinct functions in cell proliferation, survival, and differentiation. *Mol. Cell. Biol*. 24:8037-8047.

Dobin, A., C.A. Davis, F. Schlesinger, J. Drenkow, C. Zaleski, S. Jha, P. Batut, M. Chaisson, and T.R. Gingeras. 2013. STAR: ultrafast universal RNA-seq aligner. *Bioinformatics* 29:15-21.

Driegen, S., R. Ferreira, A. van Zon, J. Strouboulis, M. Jaegle, F. Grosveld, S. Philipsen, and D. Meijer. 2005. A generic tool for biotinylation of tagged proteins in transgenic mice. *Transgenic Res*. 14:477-482.

Ebert, A., S. McManus, H. Tagoh, J. Medvedovic, G. Salvagiotto, M. Novatchkova, I. Tamir, A. Sommer, M. Jaritz, and M. Busslinger. 2011. The distal V_H gene cluster of the *Igh* locus contains distinct regulatory elements with Pax5 transcription factor-dependent activity in pro-B cells. *Immunity* 34:175-187.

Fuxa, M., and M. Busslinger. 2007. Reporter gene insertions reveal a strictly B lymphoid-specific expression pattern of *Pax5* in support of its B cell identity function. *J. Immunol*. 178:3031-3037.

Hobeika, E., S. Thiemann, B. Storch, H. Jumaa, P.J. Nielsen, R. Pelanda, and M. Reth. 2006. Testing gene function early in the B cell lineage in mb1-cre mice. *Proc. Natl. Acad. Sci. USA* 103:13789-13794.

Krimpenfort, P., A. Ijpenberg, J.-Y. Song, M. van der Valk, M. Nawijn, J. Zevenhoven, and A. Berns. 2007. p15Ink4b is a critical tumour suppressor in the absence of p16Ink4a. *Nature* 448:943-946.

Kuhn, R.M., D. Haussler, and W.J. Kent. 2013. The UCSC genome browser and associated tools. *Brief. Bioinform.* 14:144-161.

Langmead, B., C. Trapnell, M. Pop, and S.L. Salzberg. 2009. Ultrafast and memory-efficient alignment of short DNA sequences to the human genome. *Genome Biol.* 10:R25.

Li, H., and R. Durbin. 2009. Fast and accurate short read alignment with Burrows-Wheeler transform. *Bioinformatics* 25:1754-1760.

Li, H., B. Handsaker, A. Wysoker, T. Fennell, J. Ruan, N. Homer, G. Marth, G. Abecasis, R. Durbin, and S. Genome Project Data Processing. 2009. The Sequence Alignment/Map format and SAMtools. *Bioinformatics* 25:2078-2079.

Liao, Y., G.K. Smyth, and W. Shi. 2014. featureCounts: an efficient general purpose program for assigning sequence reads to genomic features. *Bioinformatics* 30:923-930.

Love, M.I., W. Huber, and S. Anders. 2014. Moderated estimation of fold change and dispersion for RNA-seq data with DESeq2. *Genome Biol.* 15:550.

McCormack, M.P., A. Forster, L. Drynan, R. Pannell, and T.H. Rabbitts. 2003. The LMO2 T-cell oncogene is activated via chromosomal translocations or retroviral insertion during gene therapy but has no mandatory role in normal T-cell development. *Mol. Cell. Biol.* 23:9003-9013.

McManus, S., A. Ebert, G. Salvagiotto, J. Medvedovic, Q. Sun, I. Tamir, M. Jaritz, H. Tagoh, and M. Busslinger. 2011. The transcription factor Pax5 regulates its target genes by recruiting chromatin-modifying proteins in committed B cells. *EMBO J.* 30:2388-2404.

Minnich, M., H. Tagoh, P. Bönelt, E. Axelsson, M. Fischer, B. Cebolla, A. Tarakhovsky, S.L. Nutt, M. Jaritz, and M. Busslinger. 2016. Multifunctional role of the transcription factor Blimp-1 in coordinating plasma cell differentiation. *Nat. Immunol.* 17:331-343.

Nutt, S.L., P. Urbánek, A. Rolink, and M. Busslinger. 1997. Essential functions of Pax5 (BSAP) in pro-B cell development: difference between fetal and adult B lymphopoiesis and reduced *V*-to-*DJ* recombination at the *IgH* locus. *Genes Dev.* 11:476-491.

Ogilvy, S., D. Metcalf, C.G. Print, M.L. Bath, A.W. Harris, and J.M. Adams. 1999. Constitutive Bcl-2 expression throughout the hematopoietic compartment affects multiple lineages and enhances progenitor cell survival. *Proc. Natl. Acad. Sci. USA* 96:14943-14948.

Quinlan, A.R., and I.M. Hall. 2010. BEDTools: a flexible suite of utilities for comparing genomic features. *Bioinformatics* 26:841-842.

Revilla-i-Domingo, R., I. Bilic, B. Vilagos, H. Tagoh, A. Ebert, I.M. Tamir, L. Smeenk, J. Trupke, A. Sommer, M. Jaritz, and M. Busslinger. 2012. The B-cell identity factor Pax5 regulates distinct transcriptional programmes in early and late B lymphopoiesis. *EMBO J*. 31:3130-3146.

Rodriguez, C.I., F. Buchholz, J. Galloway, R. Sequerra, J. Kasper, R. Ayala, A.F. Stewart, and S.M. Dymecki. 2000. High-efficiency deleter mice show that FLPe is an alternative to Cre-*loxP*. *Nat. Genet*. 25:139-140.

Schwickert, T.A., H. Tagoh, K. Schindler, M. Fischer, M. Jaritz, and M. Busslinger. 2019. Ikaros prevents autoimmunity by controlling anergy and Toll-like receptor signaling in B cells. *Nat. Immunol*. 20:1517-1529.

Smeenk, L., M. Fischer, S. Jurado, M. Jaritz, A. Azaryan, B. Werner, M. Roth, J. Zuber, M. Stanulla, M.L. den Boer, C.G. Mullighan, S. Strehl, and M. Busslinger. 2017. Molecular role of the PAX5-ETV6 oncoprotein in promoting B-cell acute lymphoblastic leukemia. *EMBO J*. 36:718-735.

Souabni, A., C. Cobaleda, M. Schebesta, and M. Busslinger. 2002. Pax5 promotes B lymphopoiesis and blocks T cell development by repressing *Notch1*. *Immunity* 17:781-793.

Tallquist, M.D., and P. Soriano. 2000. Epiblast-restricted Cre expression in MORE mice: a tool to distinguish embryonic vs. extra-embryonic gene function. *Genesis* 26:113-115.

Trapnell, C., L. Pachter, and S.L. Salzberg. 2009. TopHat: discovering splice junctions with RNA-Seq. *Bioinformatics* 25:1105-1111.

Urbánek, P., Z.-Q. Wang, I. Fetka, E.F. Wagner, and M. Busslinger. 1994. Complete block of early B cell differentiation and altered patterning of the posterior midbrain in mice lacking Pax5/BSAP. *Cell* 79:901-912.

von Freeden-Jeffry, U., P. Vieira, L.A. Lucian, T. McNeil, S.E.G. Burdach, and R. Murray. 1995. Lymphopenia in interleukin (IL)-7 gene-deleted mice identifies IL-7 as a nonredundant cytokine. *J. Exp. Med*. 181:1519-1526.

Wagner, G.P., K. Kin, and V.J. Lynch. 2012. Measurement of mRNA abundance using RNA-seq data: RPKM measure is inconsistent among samples. *Theory Biosci*. 131:281-285.

Yang, H., H. Wang, C.S. Shivalila, A.W. Cheng, L. Shi, and R. Jaenisch. 2013. One-step generation of mice carrying reporter and conditional alleles by CRISPR/Cas-mediated genome engineering. *Cell* 154:1370-1379.

Yu, G., L.G. Wang, Y. Han, and Q.Y. He. 2012. clusterProfiler: an R package for comparing biological themes among gene clusters. *OMICS* 16:284-287.

Zhang, Y., T. Liu, C.A. Meyer, J. Eeckhoute, D.S. Johnson, B.E. Bernstein, C. Nussbaum, R.M. Myers, M. Brown, W. Li, and X.S. Liu. 2008. Model-based analysis of ChIP-Seq (MACS). *Genome Biol*. 9:R137.

Zhu, A., J.G. Ibrahim, and M.I. Love. 2019. Heavy-tailed prior distributions for sequence count data: removing the noise and preserving large differences. *Bioinformatics* 35:2084-2092.
