# Supplementary material for: The PAX5‐JAK2 translocation acts as dual‐hit mutation that promotes aggressive B‐cell leukemia via nuclear STAT5 activation
Source: EMBO J. 2022 Feb 14;41(7):e108397. doi: 10.15252/embj.2021108397 (PMC8982625; doi:10.15252/embj.2021108397)
Supplement: Supplementary file 2 — Table EV1 [file EMBJ-41-e108397-s005.pdf]

Table EV1: Oligonucleotide sequence information

Primers used for genotyping

Allele	Sequence (5' – 3')
All *Pax5-Jak2* variants	GGTTCTGAGGGTCCAGGAAT
	AGGTTCAGCCCTTGGAGAAT
	CGAGAACTTGTTTATTGCAGCTT
Deletion *of LoxP*-Stop-*LoxP*	CATGTCTGAGATCCCATATGAATAA (F2)
	CGACCAGCACTGTAGCACAC (R)
	GGTTCTGAGGGTCCAGGAAT (F1)
Amplification exon 2 for sequencing of *Pax5Prd*-Jak2*	AAAGGCCATCTAGGATCCTTATGTCATCCG
	TTTCCACAGCTACTTTGAATAGGGTGATCT
Amplification Pax5-Jak2 junction for sequencing *Pax5-Jak2-KD*	AGTCCCAGCTTCCAGTCAC
	TCCGTTCTTTATGTTTTTGAAGA

Primers used for quantitative genomic PCR

Allele	Sequence (5' – 3')
control 1 (35 Mb upstream of *Pax5*; Fig 3F; *Car8*)	ACAATGACCAACACGGCCAAAT
	GTTCCAGGAATGATTTCTCTC
control 2 (22 Mb downstream of Pax5; Fig S3A)	TAATCCAAGAAAGCTTTGTTA
	TCATTACTGGTCTCTTCCAGC
Pax5-Jak2	GACACCAACAAGCGCAAGAG
	CGGTCTTCAAAGGCACCAGA
Pax5	TCTCTCGGTAGGGATGATGC
	ACCCATAGTCCCAGCACATG

Primers used for ChIP verification

Site	Sequence (5' – 3')
Control	CATAGATGAAGCTGCCACATAGGT
	GTGGGCAAGGACAAAGCATTA
Site A	TAGTGTCGCCTGTGTTCTGC
	CGCTGAGGAGCTATGGATGG
Site B	CCTGCTGGAGTCCATGAGAT
	CTGACAACACTTCCGTCCCC
Site C	TGTGTGAGTGCAGATCTCATCA
	TGGCAACTGAGTGAAGCATAGA
Site D	GCCAGGGCAGGAAATGTTTG
	TGGCTAAAAGCTACGCTGGT

Primers used for gene expression analysis

Gene	Sequence (5' – 3')
Tbp	TTCGTGCAAGAAATGCTGAA
	CAGTTGTCCGTGGCTCTCTT
Pax5-Jak2	GACACCAACAAGCGCAAGAG
	CGGTCTTCAAAGGCACCAGA

sgRNAs used for CRISPR/Cas9-mediated generation of the *Pax5*(Prd*-Jak2) and *Pax5*(Jak2-KD) alleles

Gene	Sequence (5' – 3')
Pax5(Prd*-Jak2)	TCCCAGATGTAGTCCGCCAA
Pax5(Jak2-KD)	TCTACAGGACAACACTGGGG
