# Supplementary material for: The PAX5‐JAK2 translocation acts as dual‐hit mutation that promotes aggressive B‐cell leukemia via nuclear STAT5 activation
Source: EMBO J. 2022 Feb 14;41(7):e108397. doi: 10.15252/embj.2021108397 (PMC8982625; doi:10.15252/embj.2021108397)
Supplement: Supplementary file 3 — Table EV2 [file EMBJ-41-e108397-s001.pdf]

Table EV2: Description of all Illumina sequencing data used in this study (GSE174775)

GEO series #	GEO accession #	Exp #	Sample description	Genotype	Cell prepartion	Species	Sequencing protocol
GSE174775	GSM5327574	29936	Bio-ChIP-Seq Pro-B	Pax5(Bio/Bio) Rag1(Cre/Cre)	Ex-vivo	Mouse	50bp paired-end
GSE174775	GSM5327575	29938	Bio-ChIP-Seq Pro-B	Pax5(Bio/Bio) Rag1(Cre/Cre)	Ex-vivo	Mouse	50bp paired-end
GSE174775	GSM5327576	40030	Bio-ChIP-Seq B-ALL	Pax5(Jak2/+) Meox2(Cre/+)	Ex-vivo	Mouse	50bp single-end
GSE174775	GSM5327577	46051	Bio-ChIP-Seq B-ALL	Pax5(Jak2/+) Meox2(Cre/+)	Ex-vivo	Mouse	50bp single-end
GSE174775	GSM5327578	38870	Bio-ChIP-Seq Pro-B	Pax5(Jak2/+) Meox2(Cre/+)	Pro-B culture	Mouse	50bp single-end
GSE174775	GSM5327579	40031	Bio-ChIP-Seq Pro-B	Pax5(Jak2/+) Meox2(Cre/+)	Pro-B culture	Mouse	50bp single-end
GSE174775	GSM5327580	100885	ChIP-Seq H3K27ac Pro-B	Pax5(Jak2/–)	Progenitor culture	Mouse	50bp single-end
GSE174775	GSM5327581	100886	ChIP-Seq H3K27ac Pro-B	Pax5(Jak2/–)	Progenitor culture	Mouse	50bp single-end
GSE174775	GSM5327582	100884	ChIP-Seq H3K27ac Pro-B	Pax5(Prd/–)	Progenitor culture	Mouse	50bp single-end
GSE174775	GSM5327583	100887	ChIP-Seq H3K27ac Pro-B	Pax5(Prd/–)	Progenitor culture	Mouse	50bp single-end
GSE174775	GSM5327584	100893	ChIP-Seq H3K27me3 Pro-B	Pax5(Jak2/–)	Progenitor culture	Mouse	50bp single-end
GSE174775	GSM5327585	100894	ChIP-Seq H3K27me3 Pro-B	Pax5(Jak2/–)	Progenitor culture	Mouse	50bp single-end
GSE174775	GSM5327586	100892	ChIP-Seq H3K27me3 Pro-B	Pax5(Prd/–)	Progenitor culture	Mouse	50bp single-end
GSE174775	GSM5327587	100895	ChIP-Seq H3K27me3 Pro-B	Pax5(Prd/–)	Progenitor culture	Mouse	50bp single-end
GSE174775	GSM5327588	100881	ChIP-Seq H3K4me3 Pro-B	Pax5(Jak2/–)	Progenitor culture	Mouse	50bp single-end
GSE174775	GSM5327589	100882	ChIP-Seq H3K4me3 Pro-B	Pax5(Jak2/–)	Progenitor culture	Mouse	50bp single-end
GSE174775	GSM5327590	100880	ChIP-Seq H3K4me3 Pro-B	Pax5(Prd/–)	Progenitor culture	Mouse	50bp single-end
GSE174775	GSM5327591	100883	ChIP-Seq H3K4me3 Pro-B	Pax5(Prd/–)	Progenitor culture	Mouse	50bp single-end
GSE174775	GSM5327592	138003	ChIP-Seq Pax5 B-ALL	Pax5(Jak2/+)	B-ALL cell line	Mouse	50bp single-end
GSE174775	GSM5327593	138002	ChIP-Seq Pax5 B-ALL	Pax5(Jak2Prdmut/+)	B-ALL cell line	Mouse	50bp single-end
GSE174775	GSM5327594	35059	ChIP-Seq Pax5 Pro-B	wild-type C57BL/6J	Ex-vivo, FACS sorted, pooled	Mouse	50bp single-end
GSE174775	GSM5327595	35061	ChIP-Seq Pax5 Pro-B	wild-type C57BL/6J	Ex-vivo, FACS sorted, pooled	Mouse	50bp single-end
GSE174775	GSM5327596	138001	ChIP-Seq input B-ALL	Pax5(Jak2Prdmut/+)	B-ALL cell line	Mouse	50bp single-end
GSE174775	GSM5327597	35327	ChIP-Seq input Pro-B	Rag1(Cre/Cre)	Ex-vivo, FACS sorted, pooled	Mouse	50bp paired-end
GSE93764	GSM2461724	10195	ChIP-Seq input Pro-B	Rag2(–/–)	Pro-B culture	Mouse	75bp paired-end
GSE174775	GSM5327598	8265	ChIP-Seq Stat5 Pro-B	wild-type	Pro-B culture (SB+)	Mouse	36bp single-end
GSE174775	GSM5327599	36328	RNA-Seq control B-ALL	Pax5(+/–) Cdkn2ab(+/–)	Ex-vivo	Mouse	125bp paired-end
GSE174775	GSM5327600	36329	RNA-Seq control B-ALL	Pax5(+/–) Cdkn2ab(+/–)	Ex-vivo	Mouse	125bp paired-end
GSE84987	GSM2255562	17226	RNA-Seq Large pre-B	wild-type	Ex-vivo, FACS sorted	Mouse	50bp single-end
GSE84987	GSM2255569	20759	RNA-Seq Large pre-B	wild-type	Ex-vivo, FACS sorted	Mouse	50bp single-end
GSE174775	GSM5327601	36345	RNA-Seq Pax5-Jak2 B-ALL	Pax5(Jak2/+)	Ex-vivo	Mouse	125bp paired-end
GSE174775	GSM5327602	36346	RNA-Seq Pax5-Jak2 B-ALL	Pax5(Jak2/+)	Ex-vivo	Mouse	125bp paired-end
GSE174775	GSM5327603	36347	RNA-Seq Pax5-Jak2 B-ALL	Pax5(Jak2/+)	Ex-vivo	Mouse	125bp paired-end
GSE174775	GSM5327604	36348	RNA-Seq Pax5-Jak2 B-ALL	Pax5(Jak2/+)	Ex-vivo	Mouse	125bp paired-end
GSE174775	GSM5327605	24394	RNA-Seq Pro-B	Stat5(fl/+) Vav-Bcl2 Rag1(Cre/+)	Ex-vivo, FACS sorted	Mouse	50bp single-end
GSE174775	GSM5327606	24395	RNA-Seq Pro-B	Stat5(fl/+) Vav-Bcl2 Rag1(Cre/+)	Ex-vivo, FACS sorted	Mouse	50bp single-end
GSE174775	GSM5327607	24396	RNA-Seq Pro-B	Stat5(fl/fl) Vav-Bcl2 Rag1(Cre/+)	Ex-vivo, FACS sorted	Mouse	50bp single-end
GSE174775	GSM5327608	24397	RNA-Seq Pro-B	Stat5(fl/fl) Vav-Bcl2 Rag1(Cre/+)	Ex-vivo, FACS sorted	Mouse	50bp single-end
GSE174775	GSM5327609	38492	RNA-Seq Pro-B	wild-type	Ex-vivo, FACS sorted	Mouse	50bp single-end
GSE174775	GSM5327610	38493	RNA-Seq Pro-B	Pax5(–/–)	Ex-vivo, FACS sorted	Mouse	50bp single-end
GSE174775	GSM5327611	38494	RNA-Seq Pro-B	Pax5(–/–)	Ex-vivo, FACS sorted	Mouse	50bp single-end
GSE174775	GSM5327612	38496	RNA-Seq Pro-B	wild-type	Ex-vivo, FACS sorted	Mouse	50bp single-end
GSE174775	GSM5327613	38497	RNA-Seq Pro-B	wild-type	Ex-vivo, FACS sorted	Mouse	50bp single-end
GSE84987	GSM2255560	17224	RNA-Seq Pro-B	wild-type	Ex-vivo, FACS sorted	Mouse	50bp single-end
GSE174775	GSM5327614	99560	RNA-Seq Pro-B	Pax5(+/+) Meox2(Cre/+)	Ex-vivo, FACS sorted, pooled	Mouse	50bp single-end
GSE174775	GSM5327615	99561	RNA-Seq Pro-B	Pax5(+/+) Meox2(Cre/+)	Ex-vivo, FACS sorted, pooled	Mouse	50bp single-end
GSE174775	GSM5327616	99562	RNA-Seq Pro-B	Pax5(+/+) Meox2(Cre/+)	Ex-vivo, FACS sorted, pooled	Mouse	50bp single-end
GSE174775	GSM5327617	99563	RNA-Seq Pro-B	Pax5(Jak2/+) Meox2(Cre/+)	Ex-vivo, FACS sorted, pooled	Mouse	50bp single-end
GSE174775	GSM5327618	99564	RNA-Seq Pro-B	Pax5(Jak2/+) Meox2(Cre/+)	Ex-vivo, FACS sorted, pooled	Mouse	50bp single-end
GSE174775	GSM5327619	99565	RNA-Seq Pro-B	Pax5(Jak2/+) Meox2(Cre/+)	Ex-vivo, FACS sorted, pooled	Mouse	50bp single-end
GSE84987	GSM2255568	20758	RNA-Seq Pro-B	wild-type	Ex-vivo, FACS sorted	Mouse	50bp single-end
GSE84987	GSM2255561	17225	RNA-Seq Small pre-B	wild-type	Ex-vivo, FACS sorted	Mouse	50bp single-end
GSE84987	GSM2255570	20760	RNA-Seq Small pre-B	wild-type	Ex-vivo, FACS sorted	Mouse	50bp single-end
		43239	RNA-seq PAX5-JAK2+ B-ALL	PAX5-JAK2 rearrangement	Ex-vivo	Human	125bp paired-end
		43241	RNA-seq PAX5-JAK2+ B-ALL	PAX5-JAK2 rearrangement	Ex-vivo	Human	125bp paired-end
		SJBALL020069	RNA-seq PAX5-JAK2+ B-ALL	PAX5-JAK2 rearrangement	Ex-vivo	Human	100 bp paired-end
		SJBALL020138	RNA-seq PAX5-JAK2+ B-ALL	PAX5-JAK2 rearrangement	Ex-vivo	Human	100 bp paired-end
		SJBALL020936	RNA-seq PAX5-JAK2+ B-ALL	PAX5-JAK2 rearrangement	Ex-vivo	Human	100 bp paired-end
		SJBALL020966	RNA-seq PAX5-JAK2+ B-ALL	PAX5-JAK2 rearrangement	Ex-vivo	Human	100 bp paired-end
		SJBALL021329	RNA-seq PAX5-JAK2+ B-ALL	PAX5-JAK2 rearrangement	Ex-vivo	Human	100 bp paired-end
		SJBALL265	RNA-seq PAX5-JAK2+ B-ALL	PAX5-JAK2 rearrangement	Ex-vivo	Human	100 bp paired-end
