# Supplementary material for: The PAX5‐JAK2 translocation acts as dual‐hit mutation that promotes aggressive B‐cell leukemia via nuclear STAT5 activation
Source: EMBO J. 2022 Feb 14;41(7):e108397. doi: 10.15252/embj.2021108397 (PMC8982625; doi:10.15252/embj.2021108397)

## Appendix Figure S2D

Anti-Pax5

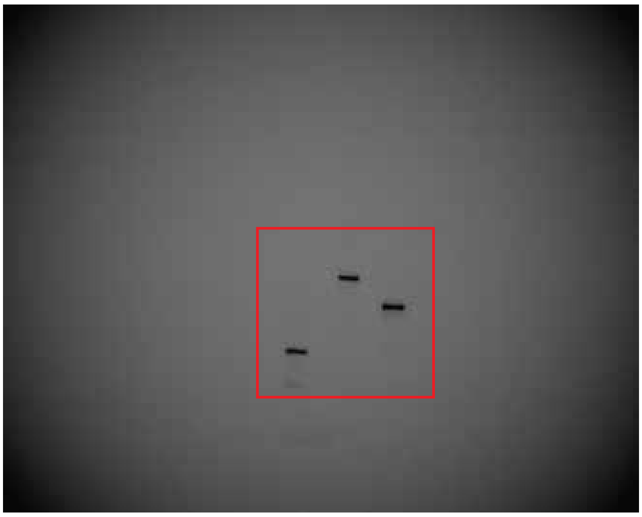

Composite image with ladder

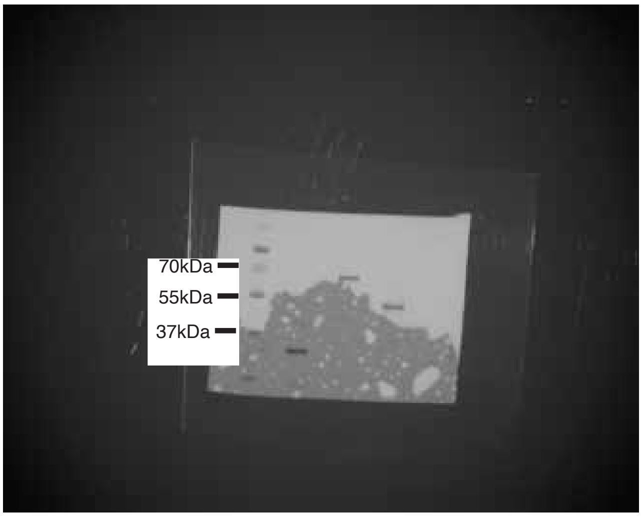


Anti-Gapdh

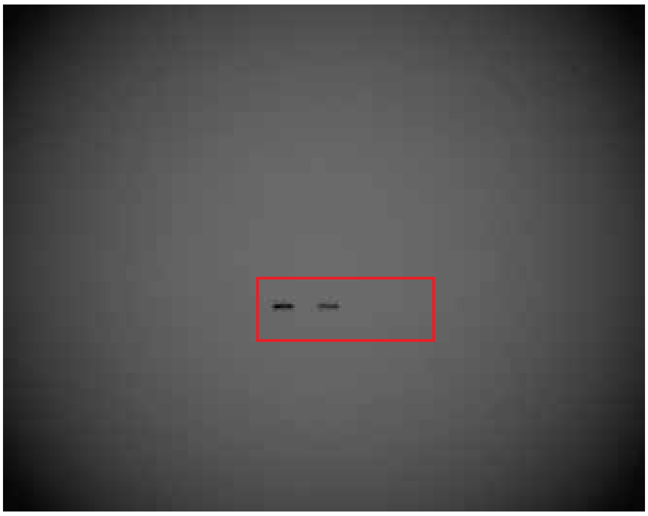

Composite image with ladder

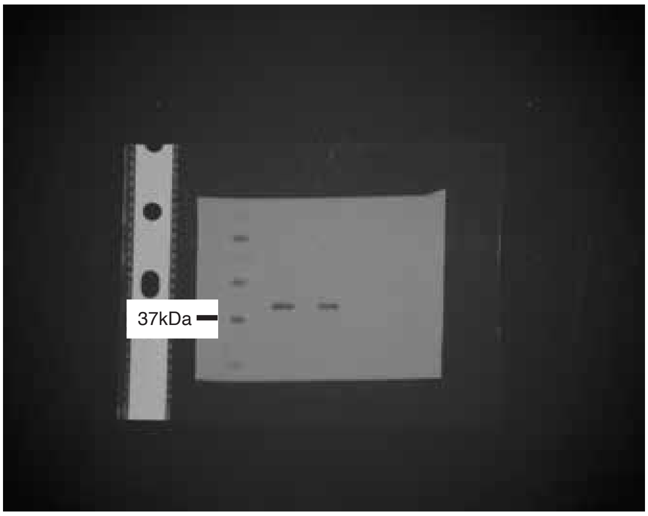

Supplement: Supplementary file 8 — Source Data for Appendix [file EMBJ-41-e108397-s006.zip › Appendix_Figure_Source_Data/EMBOJ-2021-108397R1-Appendix_Figure_S2_Source_Data-sd.pdf]
