# Supplementary figures and images for: The PAX5‐JAK2 translocation acts as dual‐hit mutation that promotes aggressive B‐cell leukemia via nuclear STAT5 activation
Source: EMBO J. 2022 Feb 14;41(7):e108397. doi: 10.15252/embj.2021108397 (PMC8982625; doi:10.15252/embj.2021108397)

## Appendix Figure S3B

DNA gel (EtBr-stained)

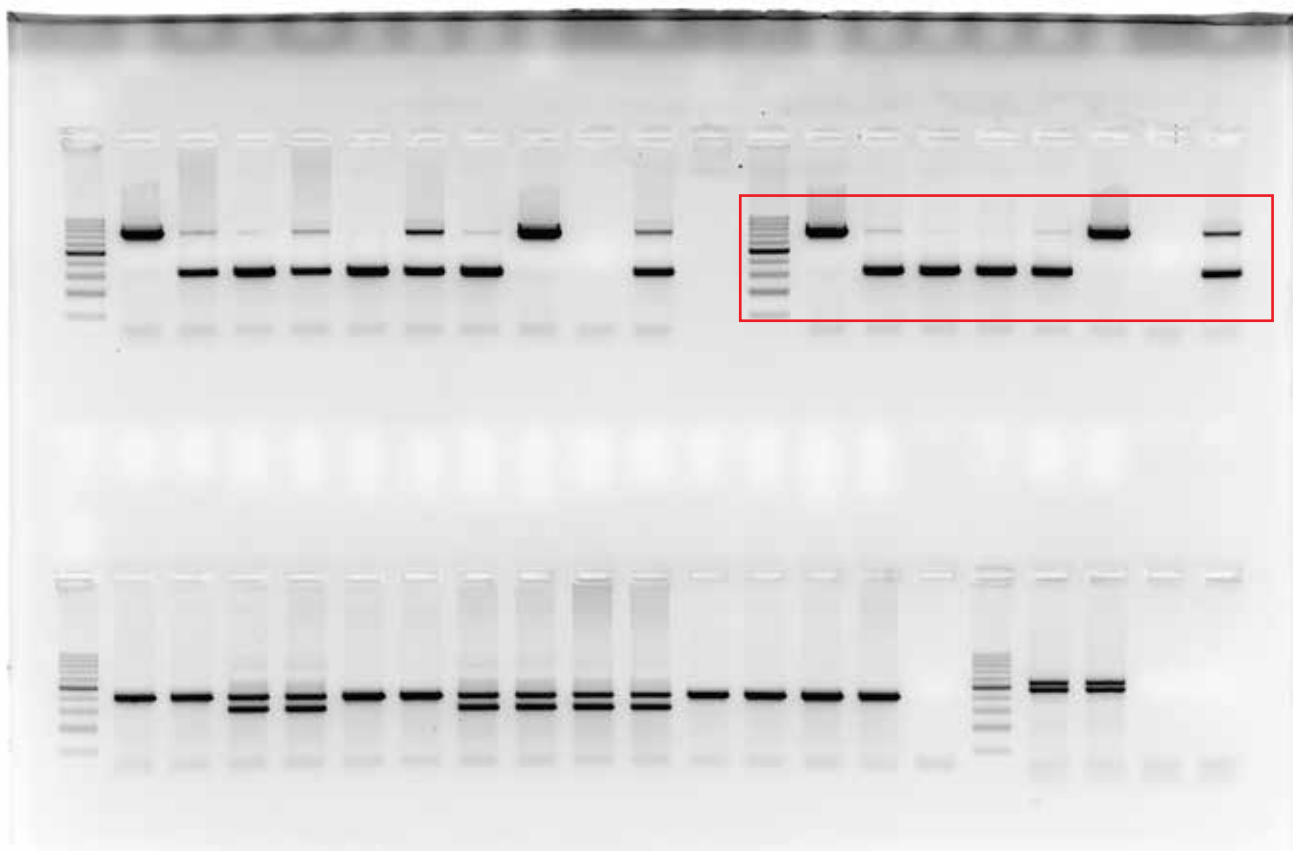

Supplement: Supplementary file 8 — Source Data for Appendix [file EMBJ-41-e108397-s006.zip › Appendix_Figure_Source_Data/EMBOJ-2021-108397R1-Appendix_Figure_S3_Source_Data-sd.pdf]

Appendix Figure S6A

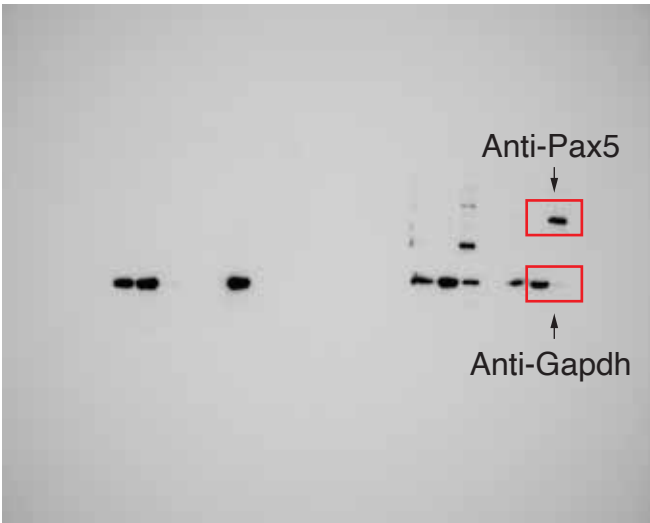

Composite image with ladder

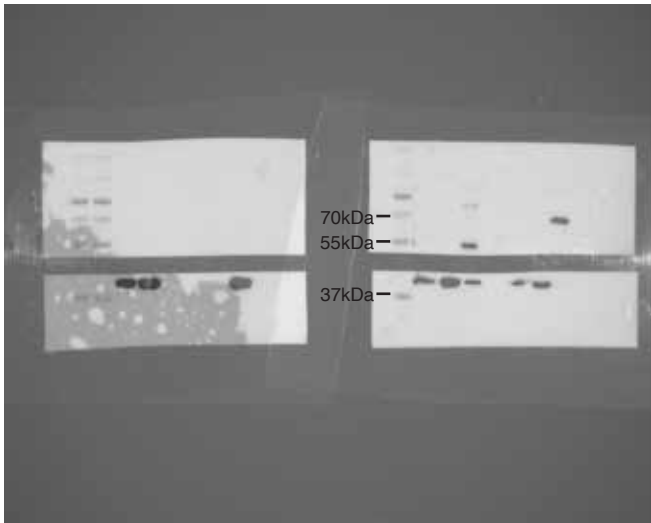

Anti-Tbp

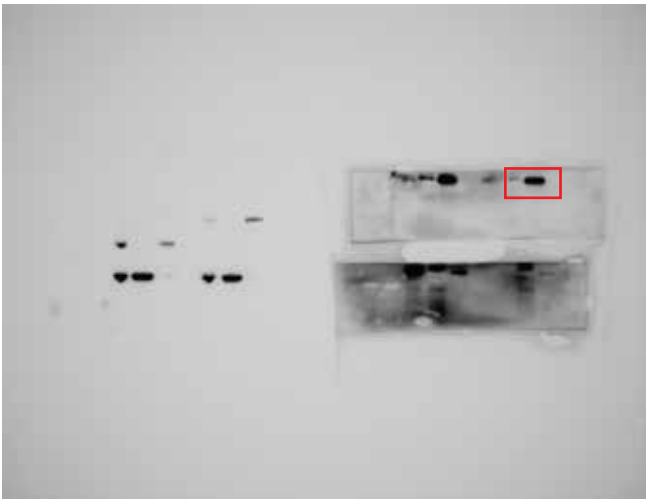

Composite image with ladder

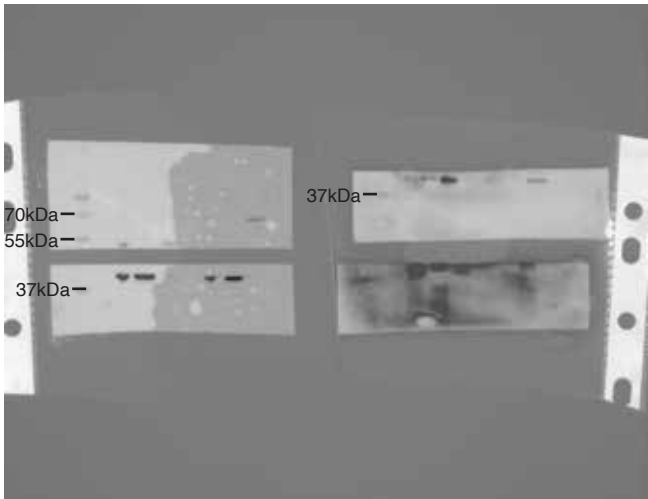

Supplement: Supplementary file 8 — Source Data for Appendix [file EMBJ-41-e108397-s006.zip › Appendix_Figure_Source_Data/EMBOJ-2021-108397R1-Appendix_Figure_S6_Source_Data-sd.pdf]

**Appendix Figure S5F**

Anti-Pax5

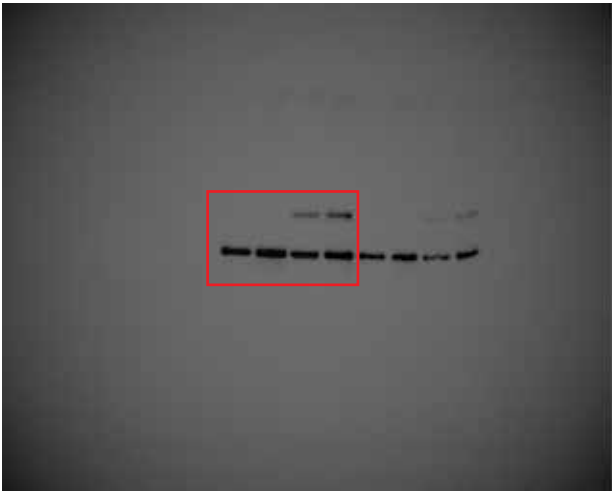

Composite image with ladder

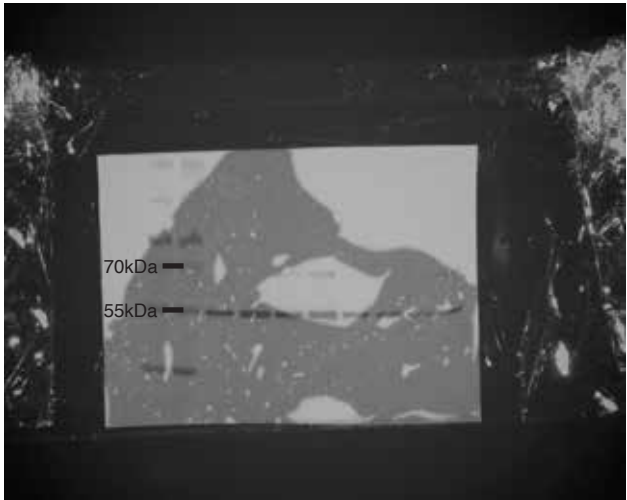

Supplement: Supplementary file 8 — Source Data for Appendix [file EMBJ-41-e108397-s006.zip › Appendix_Figure_Source_Data/EMBOJ-2021-108397R1-Appendix_Figure_S5_Source_Data-sd.pdf]

**Appendix Figure S4B**

Anti-Pax5

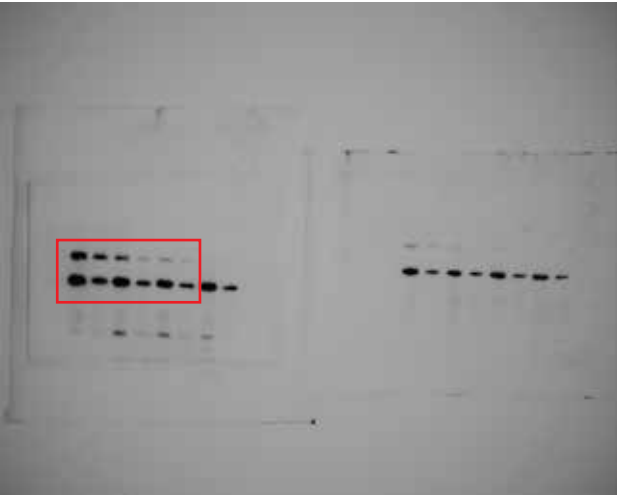

Composite image with ladder

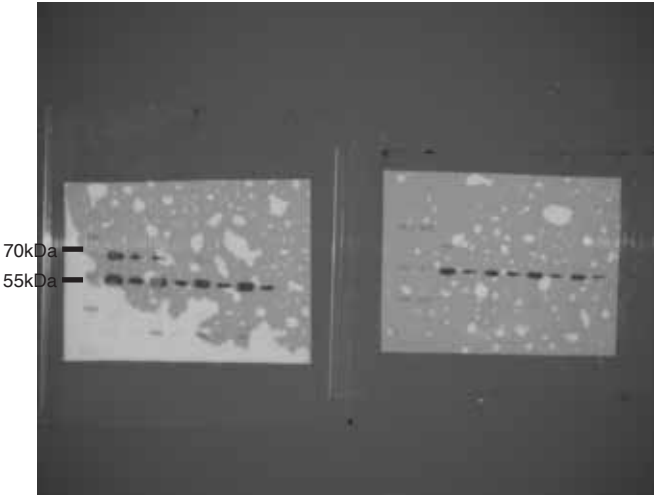

Supplement: Supplementary file 8 — Source Data for Appendix [file EMBJ-41-e108397-s006.zip › Appendix_Figure_Source_Data/EMBOJ-2021-108397R1-Appendix_Figure_S4_Source_Data-sd.pdf]

Appendix Figure S7D

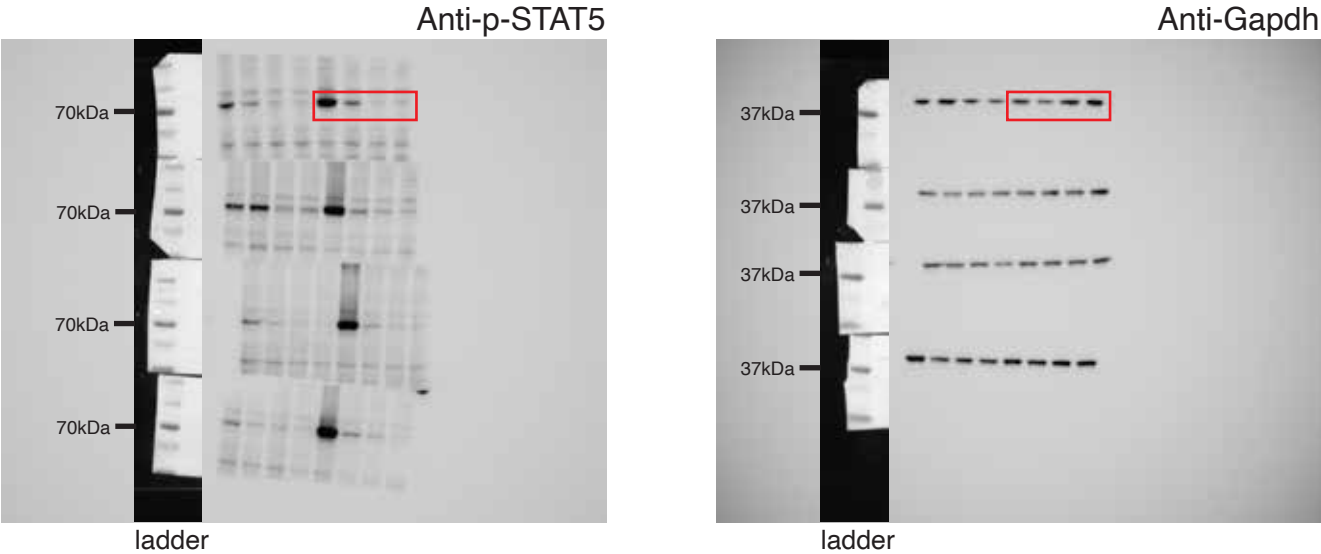

Appendix Figure S7E

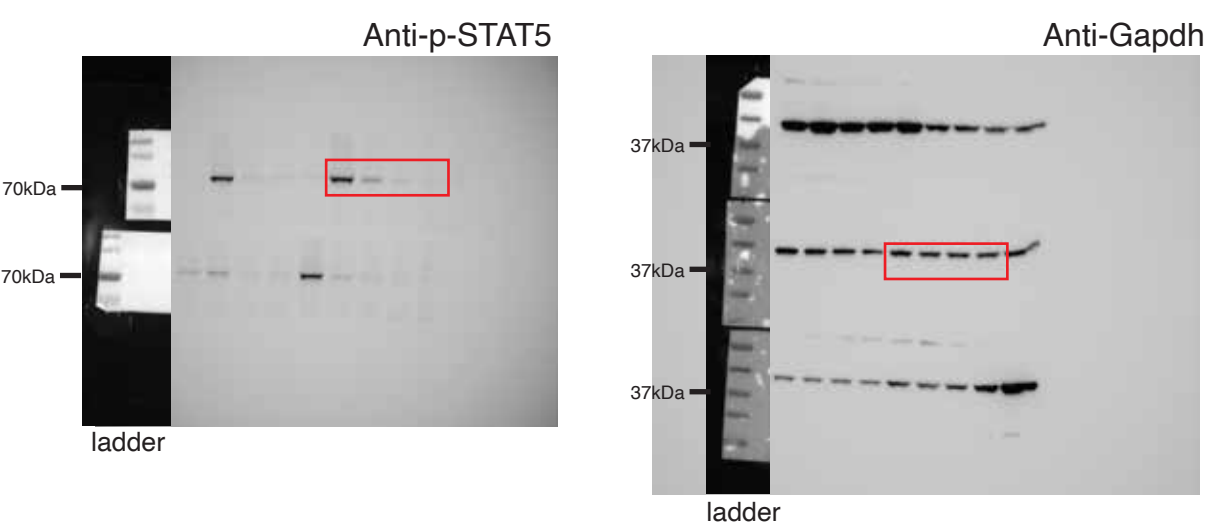

Supplement: Supplementary file 8 — Source Data for Appendix [file EMBJ-41-e108397-s006.zip › Appendix_Figure_Source_Data/EMBOJ-2021-108397R1-Appendix_Figure_S7_Source_Data-sd.pdf]

**Figure 1B**

Anti-Pax5

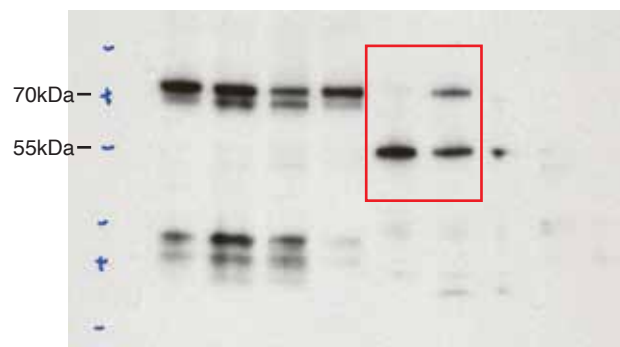

Supplement: Supplementary file 9 — Source Data for Figure 1 [file EMBJ-41-e108397-s010.pdf]

Figure 3B

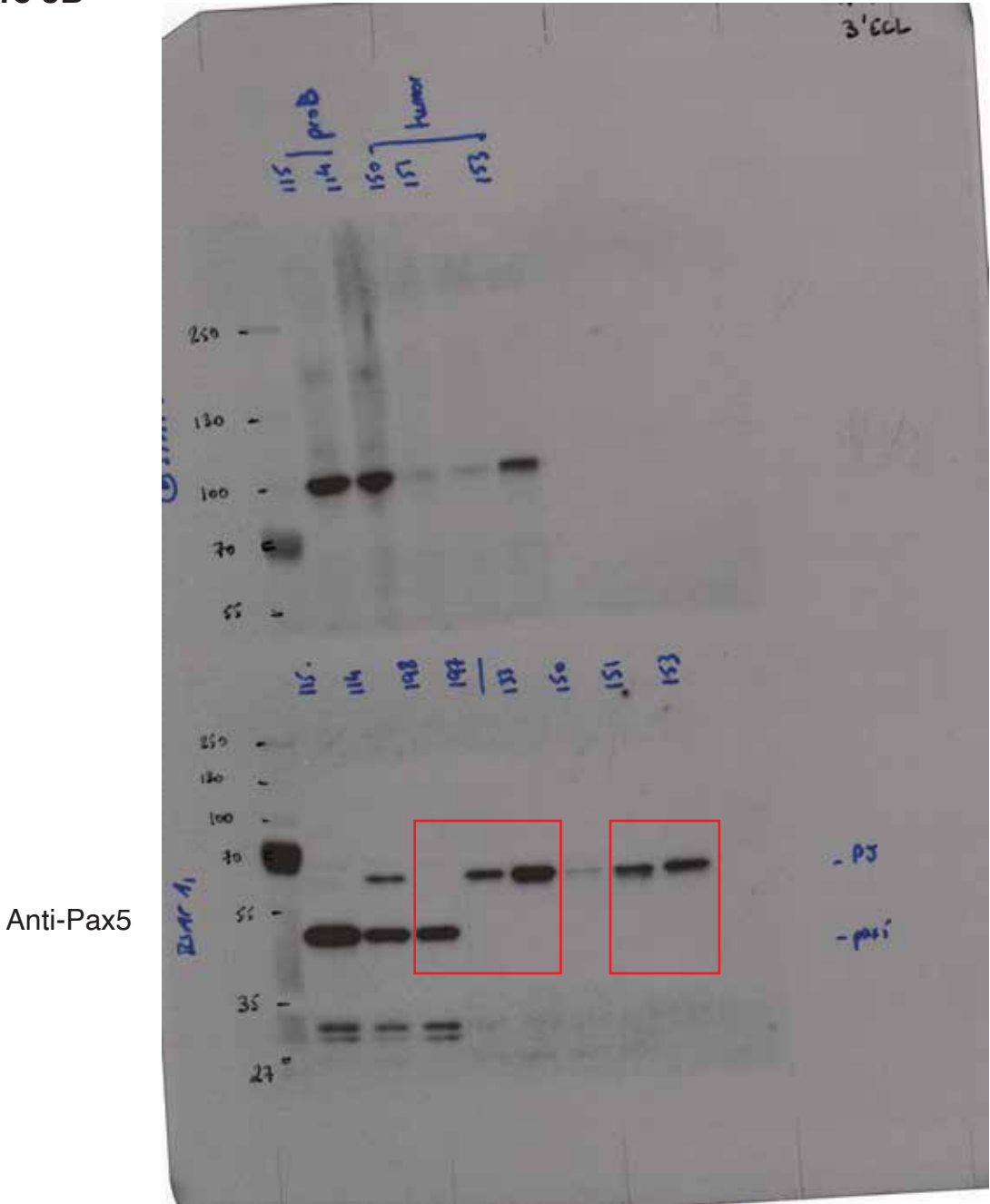

Figure 3E

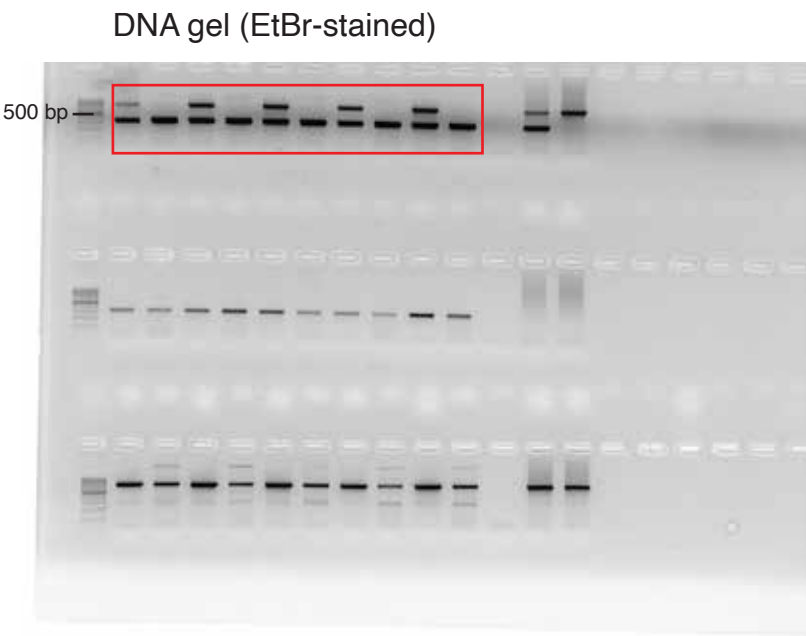

Supplement: Supplementary file 10 — Source Data for Figure 3 [file EMBJ-41-e108397-s007.pdf]

**Figure 6C**

Anti-H3Y41ph

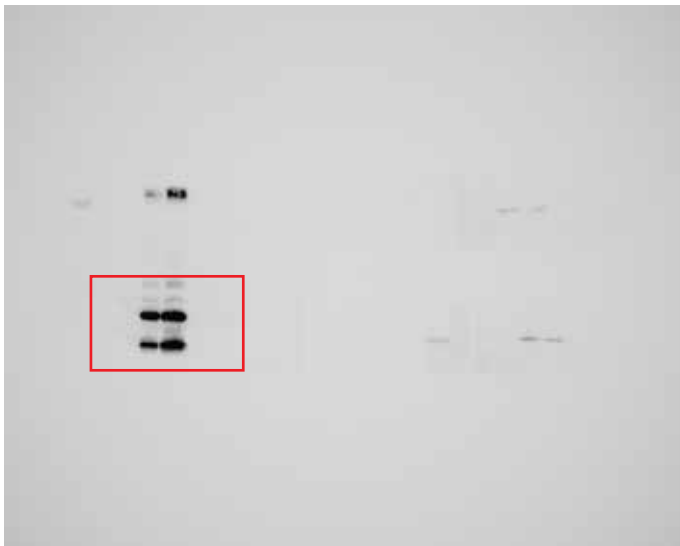

Ladder annotated printout

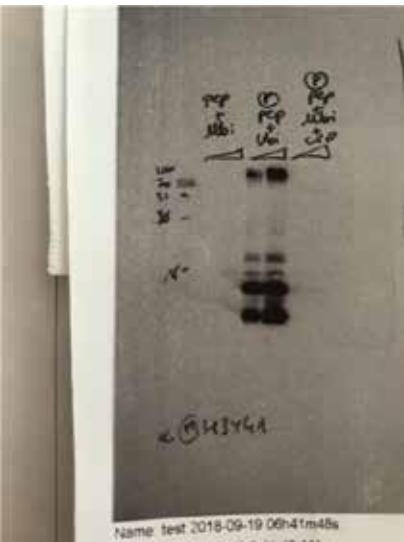

**Figure 6D**

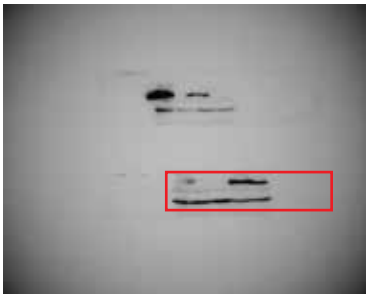

Anti-p-STAT5

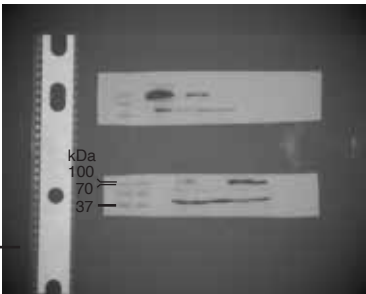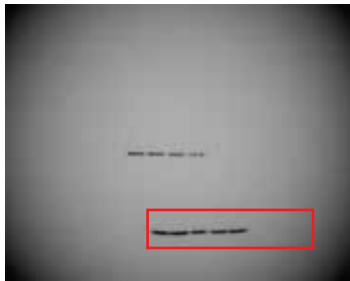

Anti-Gapdh

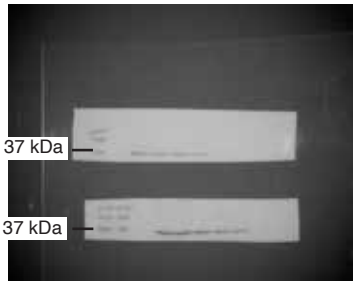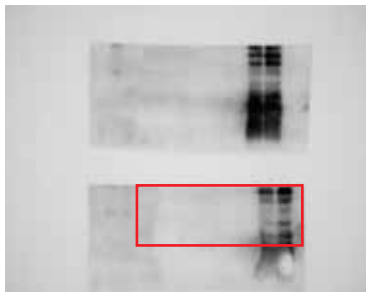

Anti-H3Y41ph

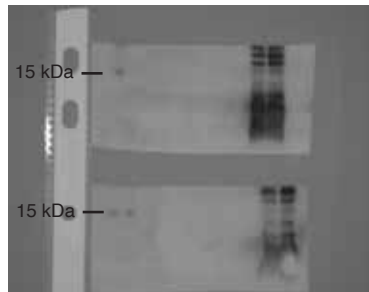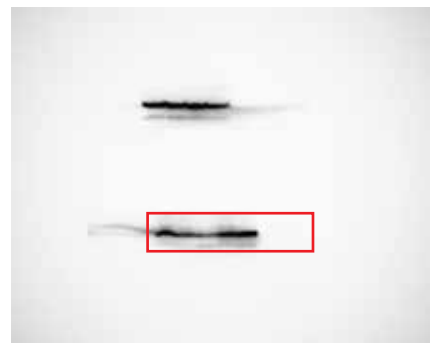

Anti-H3

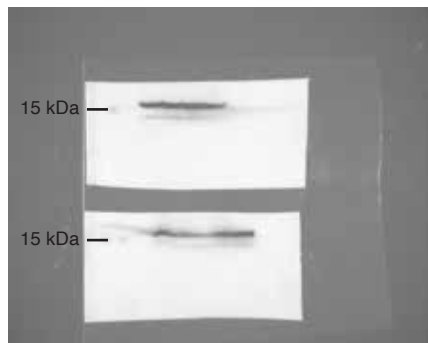

Supplement: Supplementary file 11 — Source Data for Figure 6 [file EMBJ-41-e108397-s011.pdf]
