# Supplementary material for: The PAX5‐JAK2 translocation acts as dual‐hit mutation that promotes aggressive B‐cell leukemia via nuclear STAT5 activation
Source: EMBO J. 2022 Feb 14;41(7):e108397. doi: 10.15252/embj.2021108397 (PMC8982625; doi:10.15252/embj.2021108397)
Supplement: Supplementary file 12 [file EMBJ-41-e108397-s009.pdf]

THE EMBO JOURNAL

PAX5-JAK2 acts as a dual-hit mutation to promote aggressive B cell leukemia by controlling nuclear STAT5 activity

Sabine Jurado, Anna Fedl, Markus Jaritz, Daniela Kostanova-Poliakova, Stephen Malin, Charles Mullighan, Sabine Strehl, Maria Fischer, and Meinrad Busslinger
DOI: 10.15252/embj.2021108397

Corresponding author(s): Meinrad Busslinger (Meinrad.Busslinger@imp.ac.at)

Review Timeline:

Submission Date:	2nd Apr 21
Editorial Decision:	7th May 21
Revision Received:	15th Nov 21
Editorial Decision:	16th Dec 21
Revision Received:	31st Dec 21
Accepted:	11th Jan 22

Editor: Karin Dumstrei

Transaction Report:

(Note: With the exception of the correction of typographical or spelling errors that could be a source of ambiguity, letters and reports are not edited. Depending on transfer agreements, referee reports obtained elsewhere may or may not be included in this compilation. Referee reports are anonymous unless the Referee chooses to sign their reports.)

1st Editorial Decision 7th May 2021

Dear Meinrad,

Thank you for submitting your manuscript to The EMBO Journal. Your study has now been seen by three referees and their comments are provided below.

As you can see below, the referees find the analysis interesting and support publication here. They do raise a number of constructive comments including further support for the PAX5-JAK2/ nuclear STAT5 kinase link and the cellular processes triggered by the PAX5-JAK2 fusion protein. Should you be able to address the raised concerns in full then I would like you to invite a revised version.

I think that it would be helpful to discuss the raised points further and I am available to do so via email or video.

When preparing your letter of response to the referees' comments, please bear in mind that this will form part of the Review Process File, and will therefore be available online to the community. For more details on our Transparent Editorial Process, please visit our website: https://www.embopress.org/page/journal/14602075/authorguide#transparentprocess

I thank you for the opportunity to consider your work for publication. I looking forward to discussing your revisions further

with best wishes

Karin

Karin Dumstrei, PhD
Senior Editor
The EMBO Journal

Instructions for preparing your revised manuscript:

Please make sure you upload a letter of response to the referees' comments together with the revised manuscript.

Please also check that the title and abstract of the manuscript are brief, yet explicit, even to non-specialists.

When assembling figures, please refer to our figure preparation guideline in order to ensure proper formatting and readability in print as well as on screen:
https://bit.ly/EMBOPressFigurePreparationGuideline

IMPORTANT: When you send the revision we will require
- a point-by-point response to the referees' comments, with a detailed description of the changes made (as a word file).
- a word file of the manuscript text.
- individual production quality figure files (one file per figure)
- a complete author checklist, which you can download from our author guidelines (https://www.embopress.org/page/journal/14602075/authorguide).
- Expanded View files (replacing Supplementary Information)
Please see out instructions to authors
https://www.embopress.org/page/journal/14602075/authorguide#expandedview

Please remember: Digital image enhancement is acceptable practice, as long as it accurately represents the original data and conforms to community standards. If a figure has been subjected to significant electronic manipulation, this must be noted in the figure legend or in the 'Materials and Methods' section. The editors reserve the right to request original versions of figures and the original images that were used to assemble the figure.

Further information is available in our Guide For Authors: https://www.embopress.org/page/journal/14602075/authorguide

Revision to The EMBO Journal should be submitted online within 90 days, unless an extension has been requested and approved by the editor; please click on the link below to submit the revision online before 5th Aug 2021:

https://emboj.msubmit.net/cgi-bin/main.plex

Referee #1:

The paper by Jurado et al describes the generation and characterization of a series of mouse models of B-lineage leukemia. The work is focused on the PAX5-JAK2 fusion protein generated by translocation in human leukemia. The authors use most elegant genetic approaches as well as state of the art molecular analysis to explore the impact and function of this oncogenic fusion protein in normal and malignant B-cell development. Even though the expression of this fusion protein do not appear to impact B-cell development in young mice these animals shows signs of the accumulation of a pre-leukemic clone already at 4 weeks of age. These tumors are transplantable, oligoclonal and display loss of the Wt Pax5 allele. Ectopic expression of PAX5 caused a delay in tumor progression, possibly explained by inefficient competition of the fusion protein for DNA binding. The authors report that the oncogenic activity is dependent of a functional kinase domain in the fusion protein and that mutation of the DNA binding domain modify the function of the fusion protein. While the fusion protein retained an ability to bind PAX5 sites in the genome, at least in the absence of WT PAX5, the authors do not detect support for the idea that the fusion protein has an ability to modify chromatin. As the tumors display an IL7 dependency and display signs of a hyperactivated STAT5 signaling, the authors suggest that the fusion protein act as a STAT5 kinase to retain the factor active in the nucleus once it has been trans-located to the nucleus as a consequence of IL7R signaling.

The experimental designs are in many ways elegant exploring a large variety of genetic models. The results and conclusions are, however, not always easy to follow and would in some cases appear somewhat contradictory. There also appear to be a most distinct difference between what is observed in this mouse model and human B-ALLs carrying the PAX5-JAK2 translocation. Furthermore, there is limited support for the general conclusion that the function of PAX5-JAK2 protein act as a nuclear STAT5 kinase. Hence, even though most interesting, there are several concerns that need to be addressed before the data fully support the conclusions.

Specific points:

1: I find it rather confusing that the representation of the fusion protein allele in figure 1A displays an intron between exon 4 and 5 while the remaining representations indicate that exon 4 is part of the human fusion protein. This would need clarification.

2: The authors claim that "heterozygous expression of Pax5-Jak2 had no significant effect on B cell development in young mice". These young mice are 3 weeks old, at 4 weeks many of them are abnormal. Would it not be better to discuss this in terms as "apparent effect" as it would be likely that abnormal cells exist already at three weeks of age? Furthermore, in panel 1C there appear to be an increased frequency of CD2-Kit- cells in the PAX5-JAK2 mice. Is this an actual finding or are these cells only present in the selected sample? Could the enrichment of these cells be an indication of a premalignant state? As low B220 expression levels are a characteristic of a B1 progenitor (Ghosn, PNAS 2011), can it be excluded that the leukemic cells arise from a fetal liver progenitor?

3: The data in figure S1G is somewhat difficult to interpret as these are not presented in a Kaplan-Meier plot. Were the onset of leukemia in the two mice who developed disease from B220 high cells slower than in the animals transplanted with B220 low cells? Were all the transplanted cells B220 high and B220 low from the same mouse? The figure legend states " a 6-week old mouse". Were all the transplanted cells from one mouse in one experiment? This would need clarification.

4: In figure 2, the authors use a set of RNA-seq experiments to arrive with the conclusion that "We therefore conclude that the Pax5Jak2 allele behaves like a Pax5 null allele with regard to Pax5 function". While this is all in order, I do not quite understand why the authors do not use data from the experiment presented in figure S6C where they show that a mouse carrying a PAX5 null allele and the PAX5-JAK2 allele do not obtain any CD19+ cells. Is this not direct evidence for that the fusion protein do not possess a canonical PAX5 function adding to the conclusion of the authors?

5: The analysis of the role of the kinase domain in figure 4 is interesting. While the data presented from Ruxolitinib treatment of tumor transplanted mice is elegant, the low expression levels of the KD mutated fusion allele is concerning. The onset of leukemia in Pax-Jak2-luc mice is delayed from 72 to 200 days. The authors explain this as follows "The pro-B cells of Pax5Jak2-Luc/+ mice expressed 2-fold lower levels of Pax5-Jak2 protein compared with Pax5Jak2/+ pro-B cells (Appendix Fig S1E and F). Consequently, the Pax5Jak2-Luc/+ mice develop B-ALL with a 3-fold longer latency of 214 days (Appendix Fig S4D and E) relative to the Pax5Jak2/+ mice (74 days; Fig 1F).". If the expression of the KD allele is reduced even further as indicated in figure S4D, what would then be the latency of disease onset in this mouse model? It would be important that the authors stress this limitation in their analysis.

6: The analysis of the DNA binding ability of the fusion gene is interesting. However, I cannot see that the differences in dose is taken into consideration when the CHIP-seq data are analyzed. As recently reported (Ramamoorthy et al 2020 Genes and Development) Pax5 dose impacts the binding spectra of PAX5. Inclusion of CHIP-seq data from Pax5+/- pro-B cells and tumor cells from Cd79CRE-IKXF1neo/+Pax5LSLjak2/+ mice would be most informative. As the tumors from these mice has obtained the duplication of the PAX5-Jak2 allele, the dose should possibly be comparable to that of the tumor cells from the Pax5Jak2

mice allowing for a more relevant analysis.

7: The data in figure 7 support the idea that STAT5 signaling is enhanced in the presence of the PAX5-JAK2 fusion protein. I do, however, not find it conclusively shown that STAT5 is directly targeted by the kinase. As this is an important conclusion of the paper more evidence to this end would be of great value. The hypersensitivity of the cells to IL7 signaling could possibly be addressed by short term in vitro exposure to IL7 followed by analysis of pSTAT5 levels at defined timepoints after removal of the cytokine.

Minor points:
1: The authors state in the abstract, the result section and in the discussion that "... developed an aggressive B-ALL in the absence of another cooperating mutation." , however, they report that loss of Wt Pax5 is important and occurs in the tumors. Do the authors not consider loss of PAX5 to be a mutation?

2: It would be nice to see where primers are in S1b so that S1d is easier to understand.

3: The authors write " Interestingly, a previous RNA-seq comparison of Pax5+/- and Pax5+/+ pro-B cells identified a similarly low number of Pax5-regulated genes". I do not understand if these are the same genes? If so this would likely be the result of a dose effect if not there is little information in this analysis.

4: The authors explain the reduction of B220 expression as a result of differential splicing. Did the RNA seq data support this conclusion with lower number of reads from exon 4-6?

5: The authors claim that the data in panel 3C shows absence of Wt PAX5 in the tumor cells, however, the signal in the tumors is still in the range of several thousands and no negative control is included. I would suggest that the conclusion is changed to "reduced levels".

6: The authors state " Interestingly, the PAX5-JAK2 transcripts were increased in 6 of the 8 B-ALLs, resulting in an average percentage of 68.4% for PAX5-JAK2 mRNA compared with 31.6% for full-length PAX5 mRNA (Appendix Fig S3C, right), whereas a similar analysis of PAX5-ETV6+ B-ALLs revealed a 1:1 ratio of both PAX5 transcripts (Smeenk et al., 2017).". What conclusion do the authors draw from this finding? Does this not indicate there are elements in the human fusion gene that creates higher expression levels then what is observed in the mouse model? Would this explain the apparent lack of Wt PAX5 mutations in human leukemia as the fusion protein is expressed at sufficient levels to compete with the Wt PAX5?

7: How does the conclusion " We therefore conclude that the consistent loss of Pax5 in Pax5Jak2/+ B-ALL tumors allows Pax5-Jak2 to bind to its target sites in the genome, which strongly argues for a nuclear function of Pax5-Jak2" fit with the observation that the human leukemia cells retain a functional PAX5 allele. This should be discussed. Can it be related to my minor comment 6?

8: Even though the data in figure 6 is of importance in the light of the existing literature, this information could be moved to the supplement.

9: Several diagrams, including the Kaplan-Meier plots, lacks statistical analysis.

Referee #2:

In this manuscript, Jurado et al. describe a study aimed at addressing the oncogenic function of a PAX5-JAK2 fusion protein in B-ALL. The authors generated various mouse models in which the constitutive Jak2 kinase domain is fused to the DNA-binding region of the endogenous Pax5 locus. The authors find that Pax5Jak2/+ mice rapidly develop B-ALL, whereby the tumors lose the endogenous wild type allele. The authors also introduced point mutations in the Pax5 DNA-binding region found in human B-ALL. The corresponding Pax5Prd*-Jak2/+ mice develop B-ALL with a longer survival rate and less frequent loss of the wild type Pax5 allele. ChIP-seq analysis indicated that the PAX5-Prd*-JAK2 fusion protein still binds ~50% of Pax5-sites but also gains binding at new sites. By assessing the function of the PAX5-JAK2 protein, the authors show that the fusion protein does not induce changes in chromatin modification or phosphorylation of H3Y41. However, they find that PAX5-JAK2 affects IL-7 signaling and STAT5-phosphorylation, resulting in an activation of a STAT5-dependent gene expression program.

The study addresses the important question of how PAX5-JAK5 fusions found in human B-ALL result in leukemogenesis in a mouse model. This is an impressive study that involves many newly generated mouse strains and a thorough analysis. The data are extensive, convincing and well presented. The study will be of interest to a wide readership. However, the manuscript would benefit from addressing some specific comments:

Specific comments:

1) The authors show that the PAX5-JAK2 fusion protein does not compete well with the endogenous wild-type PAX5 protein for binding to canonical Pax5 sites in pro-B cells. However, the fusion protein binds well upon the loss of the wild-type (wt) Pax5 allele in leukemic cells. In Fig. 2, the authors compared RNA-seq data sets of Pax5Jak2/+ pro-B cells with those of wt pro-B cells, as well as data of Pax5Jak2/+ B-ALL cells with those of a previously described Pax5 model of B-ALL (Pax5+/- Cdkn2ab+/-). The pro-B cell comparison identified very few differentially expressed genes (DRG), consistent with the presence of a wild-type Pax5 allele in Pax5Jak2/+ pro-B cells. In the B-ALL comparison, however, they identified a much larger set of DRGs. To assess the function of the PAX5-JAK2 fusion protein in the absence of PAX5, and to relate it to its DNA-binding ability (determined by ChIP-seq in Fig. 5), it would be of interest to include a comparison of the Pax5Jak2/+ B-ALL data set with that of Pax5+/- pro-B cells, which the authors have at hand.

2) In Fig.7, the authors show that Pax5Jak2 cooperates with IL-7 signaling in promoting B-ALL. They also find that phosphorylation of STAT5 is enhanced in Pax5Jak2 B-ALL and follicular B cells. The authors show that some 15% of STAT5-bound genes are dysregulated in Pax5Jak2 B-ALL (Fig. 7H-K). Have the authors determined whether and how many of the STAT5-bound genes are co-occupied by PAX5-JAK2? Does the constitutive active JAK2 kinase domain of the fusion protein phosphorylate co-bound STAT5 or is phosphorylation independent of DNA binding?

3) The analysis of the Pax5Prd*-Jak2/+ mice is interesting because this Pax5 allele harbors mutations in the paired domain that are frequently found in human B-ALL and alter the binding specificity (shown in Fig. S5I). Have the authors further characterized the PAX5-Prd*-JAK2-specific sites (~50% of the total bound sites)? Are different motifs enriched than those found at PAX-JAK2 or PAX5 peaks? Does binding correlate with altered chromatin accessibility?

4) The authors claim that the kinase domain is required for the oncogenic function of Pax5Jak2. This conclusion is most likely correct. However, the experiments supporting this claim should be interpreted with some caution. The genetic data involve a mouse carrying a point mutation in the kinase domain of Pax5Jak2. Based on the immunoblot analysis shown in Fig. S4B, the mutant fusion protein is expressed at a four-fold lower level than the corresponding PAX5-JAK2 protein. In other experiments, the authors find that the dosage of PAX5-JAK2 and PAX5 influences the penetrance and initiation of leukemia. Therefore, the authors may want to temper their conclusion. The effect of pharmacological inhibition of the kinase domain by treatment of cells with the JAK1/2 inhibitor Ruxolitinib is clear. However, the authors should mention that the inhibitor may also target general JAK1/2 function and not only that of the PAX5-JAK2 fusion protein.

5) The manuscript would also benefit from shortening. The result section contains description of experimental details (including generation of mouse strains) that could be moved to the Materials and Methods section: Likewise, the entire Fig. 6 and the corresponding result section is dedicated to negative data concerning the lack of epigenetic regulation by Pax5Jak2. Therefore, I suggest to include this figure in Supplemental Figures and to shorten the description in the text accordingly.

6) The authors may want to mention recent studies showing that combined Pax5 and Ebf1 heterozygosity in the mouse also enhances IL-7R signaling and STAT5 phosphorylation in B-ALL.

Referee #3:

In the manuscript entitled "PAX5-JAK2 acts as a dual-hit mutation to induce B cell leukemia by regulating nuclear STAT5 activity", the authors created a mouse model to analyze the role of PAX5-JAK2 (PJ) fusion protein in developing B-ALL in vivo. The mice expressing PJ fusion protein from endogenous PAX5 locus develop very aggressive B-ALL with median survival of 74 days. The DNA binding activity of PJ and its ability to constitutively phosphorylate Stat5 are found to be responsible for the observed phenotype. In addition, due to acquired uniparental disomy (aUPD) observed in the mice, they lose the WT PAX5 allele in the malignant B-ALL cells which accelerates the leukemia development. Although the study generates a new mouse model and characterized it, there are several questions to the model as well as the experimental results and interpretation.

Specific points:
1. The authors expressed PJ fusion gene from the endogenous PAX5 locus to closely mimic the disease situation. However, the construct led to the loss of WT PAX5 allele which is not observed in the disease. The data provided by the authors about the human B-ALL cells also supports this fact. Thus this mouse line does not actually model the human disease. It is difficult to distinguish the alterations that is caused by the oncogenic fusion protein from those caused due to loss WT PAX5 allele. From this point of view, the Cd79a-Cre Ikzf1neo/+ Pax5LSL-Jak2/+ line is a better choice of model. The longer latency period in these mice indicates that Pax5Jak2/+ model is indeed an exaggerated model for B-ALL not because of the fusion protein itself but due to model design.
2. The major focus of the study is to show the oncogenic potential of the PJ fusion in the development of B-ALL in mouse model. The oncogenic potential of PJ fusion for B-ALL is already known and the present study does not add any additional information regarding the mechanism of action. The authors did several high-throughput sequencing studies including RNA-seq and ChIP-seq. But they did not pinpoint clearly the cellular processes that are altered due to PJ fusion. They have shown the change in expression for some genes (Figure 2E, G), but what is the relevance of these genes in the context of B-ALL? Except for the explanation why B220 is lowered in the ALL cells, no further explanation has been given. From their expression data, can the

authors deduce a mechanism how after 3 weeks of age the pro-B cells rapidly transform into the malignant ALL cells?
3. The authors compared the gene expression alteration by PJ fusion protein always with respect to another B-ALL model and stressed on the PAX5 activated and repressed genes. It is well possible that the fusion protein has its own specific gene expression paradigm other than deregulating the PAX5 regulated genes. Can the authors compare the gene expression changes with respect to WT to find out these genes? This should be performed in the model expressing PJ and ectopic PAX5 as this circumvents the problem of WT PAX5 loss in the Pax5Jak2/+ model.
4. The authors concluded that the JAK2 kinase activity is required for the ALL development as the kinase dead (KD) construct leads to delay of ALL onset. However, the expression level of PJ-KD fusion is extremely low compared to PJ making it difficult to understand if the observed delay is due to loss of kinase activity of JAK2 or simply due to low expression of the oncogene. Notably, this is already the case (as also mentioned by the authors) for the Pax5Jak2-Luc/+ mice that express 2 fold less fusion protein and consequently develops ALL at 3 fold longer latency.
5. The authors show that treatment with JAK2 inhibitor Ruxolitinib prolongs the survival of the mouse by 20 days. There is no statistical analysis given for this observation (Fig 4E-F). How significant is this change? It seems that this small change also indicates that longer latency of ALL onset in kinase dead expressing mice is actually due to low expression of the oncogene.
6. Using ChIP-seq analysis, the authors analyzed the DNA binding ability of the PJ fusion protein. It is really surprising that they only concentrated on the numerical values of the binding sites occupied by PJ fusion or the paired domain mutant. Why do they not analyze the genes/ regulatory regions belonging to site B (specific for PJ fusion)? This might provide more valuable information about the oncogenic mechanism of the fusion protein. The five mutations in the PAX5 Prd domain do not eliminate DNA binding but lead to increased latency of the disease onset. It is therefore important to analyze this differential DNA binding between Prd and PJ to understand how PJ leads to such aggressive disease course. Such analysis is completely missing in the paper.
7. Regarding the epigenetic function of PJ, it is difficult to assess the data as the antibody generated in the study does not detect H3Y41Ph in cell lines previously reported to be positive for this mark. Therefore, the lack of H3Y41ph in PJ expressing cells could be due to antibody problem as well. Additionally, why did they suddenly use Pax5Jak2/- cells for the ChIP seq analysis? These cells are not even committed to B cell lineage. Thus, the Pax5Jak2/- cells and the tumor cells are temporally in different state.
8. The loss of IL7 receptor also increases disease latency. However, no statistics is provided here as well (Fig 7C and S7C). How significant is the observation? Can the authors also see alteration of IL7 regulated genes in the PJ expressing B-ALL cells in their high-throughput sequencing analysis?
9. The increased phosphorylation of Stat5 by PJ seems to be an over-estimation. The elevated pStat5 in the tumor cells is clearly evident but the increase shown in follicular (Fig 7F, G) or Pro B cells (Fig S7D, E) is really week to none. Specifically, the representative histogram plot shown in Fig S7D should not have a statistical significance shown in S7E. The authors also proposed that PJ fusion protein keeps Stat5 in constitutively phosphorylated form leading to activation of the Stat5 regulated genes. However, they did not show this constitutive activation. Can they show that in PJ expressing cells Stat5 is solely nuclear and constitutively phosphorylated?
10. The authors mentioned in the introduction that "Pax5Jak2/+ mice exhibited normal B cell development up to 3 weeks of age, but thereafter rapidly developed an aggressive B-ALL tumor in the absence of another cooperating gene mutation". The absence of other mutation is not actually tested in the study. The latency of B-ALL in the current model is too short. However, did they test any additional mutation in the ectopic PAX5 expressing mouse line?

Point-by-point reply to the reviews of the EMBOJ-2021-108397 manuscript (Jurado et al.)
(15 November 2021)

We have performed several new experiments to address the concerns of the reviewers, which has considerably improved the manuscript. We have revised the manuscript according to the reviewers' comments (in black), as indicated below in our point-by-point reply (in blue). The newly added text in the revised manuscript is highlighted in blue.

Referee #1:

The paper by Jurado et al describes the generation and characterization of a series of mouse models of B-lineage leukemia. The work is focused on the PAX5-JAK2 fusion protein generated by translocation in human leukemia. The authors use most elegant genetic approaches as well as state of the art molecular analysis to explore the impact and function of this oncogenic fusion protein in normal and malignant B-cell development. Even though the expression of this fusion protein do not appear to impact B-cell development in young mice these animals shows signs of the accumulation of a pre-leukemic clone already at 4 weeks of age. These tumors are transplantable, oligoclonal and display loss of the Wt Pax5 allele. Ectopic expression of PAX5 caused a delay in tumor progression, possibly explained by inefficient competition of the fusion protein for DNA binding. The authors report that the oncogenic activity is dependent of a functional kinase domain in the fusion protein and that mutation of the DNA binding domain modify the function of the fusion protein. While the fusion protein retained an ability to bind PAX5 sites in the genome, at least in the absence of WT PAX5, the authors do not detect support for the idea that the fusion protein has an ability to modify chromatin. As the tumors display an IL7 dependency and display signs of a hyperactivated STAT5 signaling, the authors suggest that the fusion protein act as a STAT5 kinase to retain the factor active in the nucleus once it has been trans-located to the nucleus as a consequence of IL7R signaling.

The experimental designs are in many ways elegant exploring a large variety of genetic models. The results and conclusions are, however, not always easy to follow and would in some cases appear somewhat contradictory. There also appear to be a most distinct difference between what is observed in this mouse model and human B-ALLs carrying the PAX5-JAK2 translocation. Furthermore, there is limited support for the general conclusion that the function of PAX5-JAK2 protein act as a nuclear STAT5 kinase. Hence, even though most interesting, there are several concerns that need to be addressed before the data fully support the conclusions.

Specific points:

1: I find it rather confusing that the representation of the fusion protein allele in figure 1A displays an intron between exon 4 and 5 while the remaining representations indicate that exon 4 is part of the human fusion protein. This would need clarification.

We thank the reviewer for pointing out this mistake in Figure 1A, which we have corrected.

2: The authors claim that "heterozygous expression of Pax5-Jak2 had no significant effect on B cell development in young mice". These young mice are 3 weeks old, at 4 weeks many of them are abnormal. Would it not be better to discuss this in terms as "apparent effect" as it would be likely that abnormal cells exist already at three weeks of age? Furthermore, in panel 1C there

appear to be an increased frequency of CD2-Kit- cells in the PAX5-JAK2 mice. Is this an actual finding or are these cells only present in the selected sample? Could the enrichment of these cells be an indication of a premalignant state? As low B220 expression levels are a characteristic of a B1 progenitor (Ghosn, PNAS 2011), can it be excluded that the leukemic cells arise from a fetal liver progenitor?

1) As suggested by the reviewer, we have replaced 'significant effect' by 'apparent effect' in the result section on page 7 (middle).

2) In the meantime, we have performed a statistical analysis of the flow-cytometric data of all *Pax5*$^{Jak2/+}$ and *Pax5*$^{+/+}$ mice, which did not reveal a difference in the frequency of large (FSC^{high}) or small (FSC^{low}) pre-B cells (Kit^{-}CD2^{+}B220^{+}CD19^{+}IgM^{-}IgD^{-}) between *Pax5*$^{Jak2/+}$ and *Pax5*$^{+/+}$ mice. We have now incorporated this statistical analysis in Figure 1D.

3) The low B220 expression of the *Pax5*$^{Jak2/+}$ B-ALL cells does not indicate a potential B-1 cell origin, as these tumor cells express the B-1a cell marker gene *Cd5* at a 10-fold lower level compared with wild-type peritoneal B-1a cells (Figure 1 for reviewer).

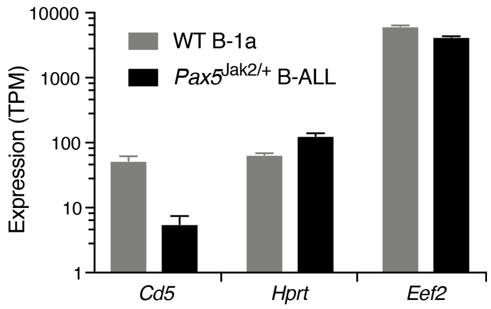

Figure 1. mRNA expression of the *Cd5* and housekeeping genes *Hprt* and *Eef2* in *Pax5*$^{Jak2/+}$ B-ALL and wild-type peritoneal B-1a cells.

3: The data in figure S1G is somewhat difficult to interpret as these are not presented in a Kaplan-Meier plot. Were the onset of leukemia in the two mice who developed disease from B220 high cells slower than in the animals transplanted with B220 low cells? Were all the transplanted cells B220 high and B220 low from the same mouse? The figure legend states " a 6-week old mouse". Were all the transplanted cells from one mouse in one experiment? This would need clarification.

We thank the reviewer for this suggestion. We now show the survival data in the format of a Kaplan-Meier survival analysis in Figure S1G, which is now easier to be understood. The B220^{high} and B220^{low} B cells were isolated from the bone marrow of one 6-week-old *Pax5*$^{Jak2/+}$ mouse (A). We now replaced "a" by "one" in the legend of Figure S1G.

4: In figure 2, the authors use a set of RNA-seq experiments to arrive with the conclusion that "We therefore conclude that the Pax5Jak2 allele behaves like a Pax5 null allele with regard to Pax5 function". While this is all in order, I do not quite understand why the authors do not use data from the experiment presented in figure S6C where they show that a mouse carrying a PAX5 null allele and the PAX5-JAK2 allele do not obtain any CD19+ cells. Is this not direct evidence for that the fusion protein do not possess a canonical PAX5 function adding to the conclusion of the authors?

We thank the reviewer for this good suggestion. In addition, we now show new flow-cytometric data in Figure S2B, which unequivocally demonstrate that the *Pax5*$^{Jak2/+}$ and *Pax5*$^{Prd/+}$ mice are unable to generate CD19^{+} B cells. As suggested, we have moved the data of Figure S6C,D to Figure S2C,D and now discuss these and the new data immediately after the presentation of the minimal gene expression differences observed between *Pax5*$^{Jak2/+}$ and *Pax5*$^{+/+}$ pro-B cells in 3-week-old mice (page 9, bottom). The fact that B cell development is arrested at an uncommitted

B220$^+$CD19$^-$ progenitor stage in *Pax5*$^{Jak2/-}$ mice unequivocally demonstrates that Pax5-Jak2 does not function as a transcription factor. It is important for the reader to realize early in the result section that the differential gene expression observed in *Pax5*$^{Jak2/+}$ B-ALL cells is not caused by a "direct transcriptional activity" of the Pax5-Jak2 protein but rather by an indirect effect of the constitutively active Pax5-Jak2 kinase.

5: The analysis of the role of the kinase domain in figure 4 is interesting. While the data presented from Ruxolitinib treatment of tumor transplanted mice is elegant, the low expression levels of the KD mutated fusion allele is concerning. The onset of leukemia in Pax-Jak2-luc mice is delayed from 72 to 200 days. The authors explain this as follows "The pro-B cells of Pax5Jak2-Luc/+ mice expressed 2-fold lower levels of Pax5-Jak2 protein compared with Pax5Jak2/+ pro-B cells (Appendix Fig S1E and F). Consequently, the Pax5Jak2-Luc/+ mice develop B-ALL with a 3-fold longer latency of 214 days (Appendix Fig S4D and E) relative to the Pax5Jak2/+ mice (74 days; Fig 1F).". If the expression of the KD allele is reduced even further as indicated in figure S4D, what would then be the latency of disease onset in this mouse model? It would be important that the authors stress this limitation in their analysis.

As suggested by the reviewer, we now mention the potential limitation caused by the lower expression of the Pax5-Jak2-KD protein in *Pax5*$^{Jak2\text{-}KD/+}$ B cells (Figure S4B) by making the following statement on page 16 (top): "While the 4-fold lower expression of the Pax5-Jak2-KD protein in pro-B cells is expected to significantly delay the tumor onset, the complete absence of B-ALL in 1-year-old *Pax5*$^{Jak2\text{-}KD/+}$ mice nevertheless indicates a critical role of the Pax5-Jak2 kinase activity in the initiation of leukemia development".

6: The analysis of the DNA binding ability of the fusion gene is interesting. However, I cannot see that the differences in dose is taken into consideration when the CHIP-seq data are analyzed. As recently reported (Ramamoorthy et al 2020 Genes and Development) Pax5 dose impacts the binding spectra of PAX5. Inclusion of CHIP-seq data from Pax5+/- pro-B cells and tumor cells from Cd79CRE-IKXF1neo/+Pax5LSLjak2/+ mice would be most informative. As the tumors from these mice has obtained the duplication of the PAX5-Jak2 allele, the dose should possibly be comparable to that of the tumor cells from the Pax5Jak2 mice allowing for a more relevant analysis.

1) In order to perform a Bio-ChIP-seq experiment with *Pax5*$^{Bio/-}$ pro-B cells (instead of Pax5$^{+/-}$ pro-B cells) to fit it to the Bio-ChIP-seq data of Figure 5B, we would have had to first generate mice of the *Rosa26*$^{BirA/+}$ *Pax5*$^{Bio/-}$ genotype followed by *in vitro* culture of pro-B cells and subsequent Bio-ChIP-seq analysis. We did not perform this experiment, as we do not consider the long time delay caused by this experiment to be justified particularly in view of the relatively high variability of results obtained with ChIP-seq experiments.

2) There is a misunderstanding about the utility of *Cd79a*-Cre *Ikzf1*$^{neo/+}$ *Pax5*$^{LSL\text{-}Jak2/+}$ B-ALL cells for Bio-ChIP-seq analysis. Due to ectopic expression of the full-length Pax5 protein from the *Ikzf1* allele, the Pax5-Jak2 protein can only inefficiently bind to genomic DNA in the presence of the competing full-length Pax5 protein, as we described it for the *Pax5*$^{Jak2/+}$ *Rosa26*$^{BirA/+}$ pro-B cells in Figure 5B. Moreover, we have not established a cell line from a *Cd79a*-Cre *Ikzf1*$^{neo/+}$ *Pax5*$^{LSL\text{-}Jak2/+}$ B-ALL tumor that should additionally even express *Rosa26*^{BirA} to be used for Bio-ChIP analysis. Due to the relatively long tumor latency (Figure 3C), we cannot even predict, when we would obtain the next *Cd79a*-Cre *Ikzf1*$^{neo/+}$ *Pax5*$^{LSL\text{-}Jak2/+}$ B-ALL tumor mouse. Hence, the suggested experiment is not feasible for scientific and time reasons.

7: The data in figure 7 support the idea that STAT5 signaling is enhanced in the presence of the PAX5-JAK2 fusion protein. I do, however, not find it conclusively shown that STAT5 is directly targeted by the kinase. As this is an important conclusion of the paper more evidence to this end would be of great value. The hypersensitivity of the cells to IL7 signaling could possibly be addressed by short term in vitro exposure to IL7 followed by analysis of pSTAT5 levels at defined timepoints after removal of the cytokine.

1) To address the first point (phosphorylation of STAT5 by the Pax5-Jak2 kinase), we have performed a time course analysis by treating *Pax5*$^{Jak2/+}$ B-ALL cells with the JAK1/2 inhibitor ruxolitinib followed by immunoblot analysis of p-STAT5. Phosphorylation of STAT5 was reduced within 30 min of ruxolitinib treatment and was lost after 1 hour, demonstrating that the p-STAT5 is strongly dependent on Jak2 activity in *Pax5*$^{Jak2/+}$ B-ALL cells (shown in the new Figure S7D). These data are mentioned on page 24 (top).

2) We performed the suggested time course analysis to investigate STAT5 phosphorylation after IL-7 withdrawal in *Pax5*$^{Jak2/+}$ B-ALL cells. Upon IL-7 withdrawal, STAT5 phosphorylation was reduced at 2 hours and lost at 4 hours in *Pax5*$^{Jak2/+}$ B-ALL cells, indicating that p-STAT5 in tumor cells is dependent on IL-7 signaling (shown in the new Figure S7E). These data are mentioned on page 24 (top). The loss of p-STAT5 is furthermore consistent with the observed loss of cell viability of *Pax5*$^{Jak2/+}$ B-ALL cells in response to IL-7 withdrawal, as shown in Figure S7A of the initial submission.

Minor points:

1: The authors state in the abstract, the result section and in the discussion that "... developed an aggressive B-ALL in the absence of another cooperating mutation.", however, they report that loss of Wt Pax5 is important and occurs in the tumors. Do the authors not consider loss of PAX5 to be a mutation?

As suggested by the reviewer, we have rephrased the statement by indicating that aggressive B-ALL tumors develop in *Pax5*$^{Jak2/+}$ mice without the need of introducing another cooperating exogeneous gene mutation in the abstract (page 2), introduction (page 5) and discussion (page 26).

2: It would be nice to see where primers are in S1b so that S1d is easier to understand.

We now show the location of the PCR primers in Figure S1B, which helps to better understand the PCR result shown in Figure S1D.

3: The authors write " Interestingly, a previous RNA-seq comparison of Pax5+/- and Pax5+/+ pro-B cells identified a similarly low number of Pax5-regulated genes". I do not understand if these are the same genes? If so this would likely be the result of a dose effect if not there is little information in this analysis.

The low number of Pax5-regulated genes in *Pax5*$^{Jak2/+}$ and *Pax5*$^{+/–}$ pro-B cells are indeed caused by a dose effect, as the *Pax5*^{Jak2} allele behaves like a null allele with regard to Pax5 function, based on the failure of *Pax5*$^{Jak2/–}$ mice to develop any CD19^{+} B cells due to a stringent B cell developmental arrest at an uncommitted progenitor stage (Figure S2B,C). We now explicitly state this fact on page 9 (bottom). Consequently, there is indeed a significant overlap between the Pax5-regulated genes in *Pax5*$^{Jak2/+}$ and *Pax5*$^{+/–}$ pro-B cells, as suggested by the reviewer.

4: The authors explain the reduction of B220 expression as a result of differential splicing. Did the RNA seq data support this conclusion with lower number of reads from exon 4-6?

The RNA-seq data shown in Figure 2H of the initial submission already demonstrate that the reads at the exons 4, 5 and 6 of the *Ptprc* (CD45) gene are strongly reduced in *Pax5*$^{Jak2/+}$ B-ALL cells compared with control *Pax5*$^{+/-}$ *Cdkn2ab*$^{+/-}$ B-ALL cells.

5: The authors claim that the data in panel 3C shows absence of Wt PAX5 in the tumor cells, however, the signal in the tumors is still in the range of several thousands and no negative control is included. I would suggest that the conclusion is changed to "reduced levels".

Four lines of evidence indicate that Pax5 is completely lost in *Pax5*$^{Jak2/+}$ B-ALL cells and is not only reduced in these cells as suggested by the reviewer. The tumor cells express no wild-type mRNA (Figure 3A), no wild-type protein (Figure 3B), no hCD2 (Pax5) reporter protein (Figure 3D) and have lost the wild-type *Pax5* gene by uniparental disomy (Figures 3E and S3A). The relatively high fluorescence intensity measured in *Pax5*$^{Jak2/+}$ B-ALL cells by intracellular staining with the anti-Pax5 antibody therefore corresponds to the loss of Pax5 and cannot be interpreted as 'reduced Pax5 levels'.

6: The authors state " Interestingly, the PAX5-JAK2 transcripts were increased in 6 of the 8 B-ALLs, resulting in an average percentage of 68.4% for PAX5-JAK2 mRNA compared with 31.6% for full-length PAX5 mRNA (Appendix Fig S3C, right), whereas a similar analysis of PAX5-ETV6+ B-ALLs revealed a 1:1 ratio of both PAX5 transcripts (Smeenk et al., 2017).". What conclusion do the authors draw from this finding? Does this not indicate there are elements in the human fusion gene that creates higher expression levels then what is observed in the mouse model? Would this explain the apparent lack of Wt PAX5 mutations in human leukemia as the fusion protein is expressed at sufficient levels to compete with the Wt PAX5?

The explanation of the reviewer may be correct, as regulatory elements of the *JAK2* locus could increase *PAX5-JAK2* expression in the respective human B-ALLs. However, we did not dare to draw such a strong conclusion, given the high fluctuation of the data between the different PAX5-JAK2^{+} patients (from 85.2% [tumor 1] to 46.6% [tumor 4]).

7: How does the conclusion " We therefore conclude that the consistent loss of Pax5 in Pax5Jak2/+ B-ALL tumors allows Pax5-Jak2 to bind to its target sites in the genome, which strongly argues for a nuclear function of Pax5-Jak2" fit with the observation that the human leukemia cells retain a functional PAX5 allele. This should be discussed. Can it be related to my minor comment 6?

The difference between the mouse *Pax5*$^{Jak2/+}$ B-ALL model and the human PAX5-JAK2^{+} B-ALLs is discussed in great detail on page 28 in the discussion section of the initial submission, where we mention that the loss of Pax5 accelerates B-ALL formation but is not essential for B-ALL development in the mouse model similar to the human patients.

8: Even though the data in figure 6 is of importance in the light of the existing literature, this information could be moved to the supplement.

The reviewer acknowledges the importance of the H3Y41ph data in light of the existing literature. The issue of H3Y41 phosphorylation has been an unresolved and hotly debated issue in both the epigenetics and JAK-STAT communities. We therefore think that we should not hide the data in a supplementary figure, especially when we have an entire paragraph dedicated to

the phosphorylation of H3Y41 and even mention these epigenetic data in the abstract. Sometimes, it is also important to show well-controlled negative data. Finally, we have generated a monoclonal anti-H3Y41ph antibody and could demonstrate with this new tool that this histone modification does not exist. As this message would be lost by relegation of the data to a supplementary figure, we strongly argue for showing these data in Figure 6.

9: Several diagrams, including the Kaplan-Meier plots, lacks statistical analysis.

As suggested by the reviewer, we have now added the statistical evaluation to the Kaplan-Meier plots in Figures 1F, 3G, 4F, 5H and 7C as well as in Figures S1G, S4D and S5H. In addition, we have also provided the statistical data for Figures 3F and 7B as well as for Figures S1F, S3A and S4G.

Referee #2:

In this manuscript, Jurado et al. describe a study aimed at addressing the oncogenic function of a PAX5-JAK2 fusion protein in B-ALL. The authors generated various mouse models in which the constitutive Jak2 kinase domain is fused to the DNA-binding region of the endogenous Pax5 locus. The authors find that Pax5Jak2/+ mice rapidly develop B-ALL, whereby the tumors lose the endogenous wild type allele. The authors also introduced point mutations in the Pax5 DNA-binding region found in human B-ALL. The corresponding Pax5Prd*-Jak2/+ mice develop B-ALL with a longer survival rate and less frequent loss of the wild type Pax5 allele. ChIP-seq analysis indicated that the PAX5-Prd*-JAK2 fusion protein still binds ~50% of Pax5-sites but also gains binding at new sites. By assessing the function of the PAX5-JAK2 protein, the authors show that the fusion protein does not induce changes in chromatin modification or phosphorylation of H3Y41. However, they find that PAX5-JAK2 affects IL-7 signaling and STAT5-phosphorylation, resulting in an activation of a STAT5-dependent gene expression program.

The study addresses the important question of how PAX5-JAK5 fusions found in human B-ALL result in leukemogenesis in a mouse model. This is an impressive study that involves many newly generated mouse strains and a thorough analysis. The data are extensive, convincing and well presented. The study will be of interest to a wide readership. However, the manuscript would benefit from addressing some specific comments.

Specific comments:

1) The authors show that the PAX5-JAK2 fusion protein does not compete well with the endogenous wild-type PAX5 protein for binding to canonical Pax5 sites in pro-B cells. However, the fusion protein binds well upon the loss of the wild-type (wt) Pax5 allele in leukemic cells. In Fig. 2, the authors compared RNA-seq data sets of Pax5Jak2/+ pro-B cells with those of wt pro-B cells, as well as data of Pax5Jak2/+ B-ALL cells with those of a previously described Pax5 model of B-ALL (Pax5+/- Cdkn2ab+/-). The pro-B cell comparison identified very few differentially expressed genes (DRG), consistent with the presence of a wild-type Pax5 allele in Pax5Jak2/+ pro-B cells. In the B-ALL comparison, however, they identified a much larger set of DRGs. To assess the function of the PAX5-JAK2 fusion protein in the absence of PAX5, and to relate it to its DNA-binding ability (determined by ChIP-seq in Fig. 5), it would be of interest to include a comparison of the Pax5Jak2/+ B-ALL data set with that of Pax5+/- pro-B cells, which the authors have at hand.

It is a notorious problem in tumor biology to define a control cell type or developmental stage for a meaningful comparison of expression data with a given tumor cell type. The proliferating large pre-B cells can be used as a reference cell type if a B-ALL tumor is arrested at the pre-BCR^{+} cell stage. Our PCA analysis of RNA-seq data demonstrated that the *Pax5*$^{Jak2/+}$ B-ALL cells are positioned between wild-type large pre-B cells and wild-type pro-B cells on the PCA plot (Figure S2E) due to the partial dedifferentiation of *Pax5*$^{Jak2/+}$ B-ALL cells caused by their Pax5 loss. We have bioinformatically analyzed the RNA-seq comparisons between *Pax5*$^{Jak2/+}$ B-ALL and *Pax5*$^{+/+}$ pro-B cells (left), between *Pax5*$^{Jak2/+}$ B-ALL (middle) and *Pax5*$^{+/–}$ pro-B cells as well as between *Pax5*$^{Jak2/+}$ B-ALL and *Pax5*$^{+/+}$ large pre-B cells (right; Figure 2 for reviewer). These comparisons identified many more differentially expressed genes, which is not surprising, as we compare transformed tumor cells with primary pro-B and large pre-B cells. These massive gene expression changes must be largely caused by the tumor versus primary cell state comparison and may have little to do with the Pax5-Jak2 protein, which is mainly involved in phosphorylating STAT5 and does not act as a transcription factor (Figures 7 and S2B,C). For these reasons, we did not further analyze the massive gene expression changes shown in Figure 2 (for reviewer).

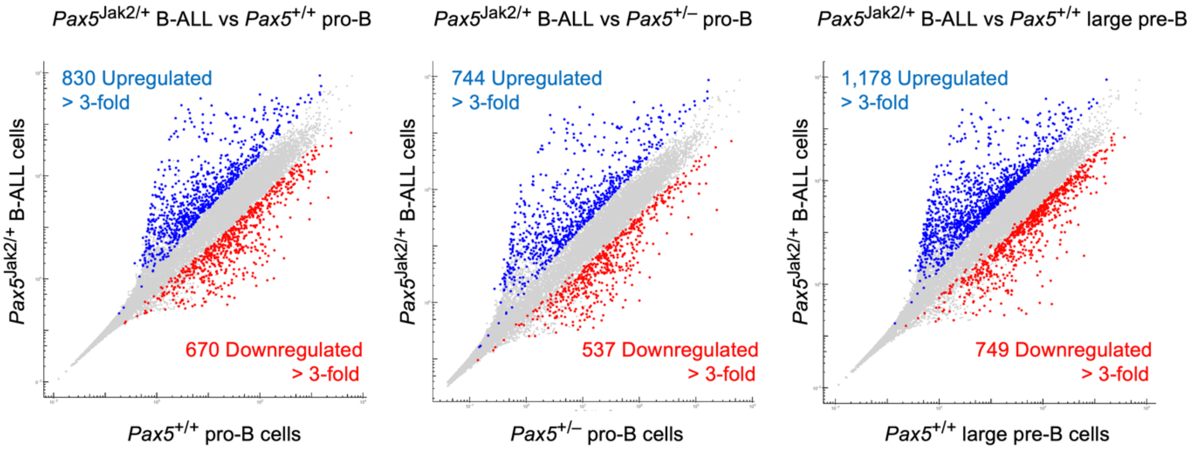

Figure 2. Gene expression differences between *Pax5*$^{Jak2/+}$ B-ALL and the indicated pro-B and pre-B cells, which were characterized by $P_{adj} < 0.05$, fold-change > 3-fold and expression value > 10 TPM.

2) In Fig.7, the authors show that Pax5Jak2 cooperates with IL-7 signaling in promoting B-ALL. They also find that phosphorylation of STAT5 is enhanced in Pax5Jak2 B-ALL and follicular B cells. The authors show that some 15% of STAT5-bound genes are dysregulated in Pax5Jak2 B-ALL (Fig. 7H-K). Have the authors determined whether and how many of the STAT5-bound genes are co-occupied by PAX5-JAK2? Does the constitutive active JAK2 kinase domain of the fusion protein phosphorylate co-bound STAT5 or is phosphorylation independent of DNA binding?

1) We have performed the requested bioinformatic analysis, which revealed that the STAT5-bound target genes constitute 11.4% of all Pax5-Jak2-bound genes in *Pax5*$^{Jak2/+}$ B-ALL cells. This overlap is likely an underestimate as only 2,377 STAT5 peaks were identified by ChIP-seq in pro-B cells.

2) The second question (phosphorylation of co-bound STAT5 by Pax5-Jak2) is extremely difficult, if not impossible, to answer. For this, we should have performed sequential ChIP analysis by first precipitating Pax5-Jak2-bound DNA followed by re-precipitation of the bound DNA with an anti-phospho-STAT5 antibody. Sequential ChIP analysis is a largely uncontrolled experiment, as the precipitated DNA after the second ChIP cannot be normalized to any other DNA sequences,

which have been eliminated during the second precipitation. For this reason, we did not attempt to perform this experiment.

3) The analysis of the Pax5Prd*-Jak2/+ mice is interesting because this Pax5 allele harbors mutations in the paired domain that are frequently found in human B-ALL and alter the binding specificity (shown in Fig. S5I). Have the authors further characterized the PAX5-Prd*-JAK2-specific sites (~50% of the total bound sites)? Are different motifs enriched than those found at PAX5-JAK2 or PAX5 peaks? Does binding correlate with altered chromatin accessibility?

As suggested by the reviewer, we have further analyzed the ChIP-seq data of the *Pax5*$^{Prd*-Jak2/+}$ and *Pax5*$^{Jak2/+}$ B-ALL cells (Figure S5I of the manuscript). Figure 3 (for reviewer, below) shows the density of the common (A), Pax5-Prd*-Jak2-specific (B) and Pax5-Jak2-specific (C) peaks. While the *de novo* motif discovery program MEME-ChIP readily identified the consensus Pax5 recognition motif in the common peaks (A), we could not find any significant *de novo*-predicted motif in the Pax5-Prd*-Jak2-specific (B) and Pax5-Jak2-specific (C) peaks. We have not performed ATAC-seq experiments with *Pax5*$^{Jak2/+}$ and *Pax5*$^{Prd*-Jak2\ /+}$ B-ALL cells and are therefore not in a position to comment on altered chromatin accessibility.

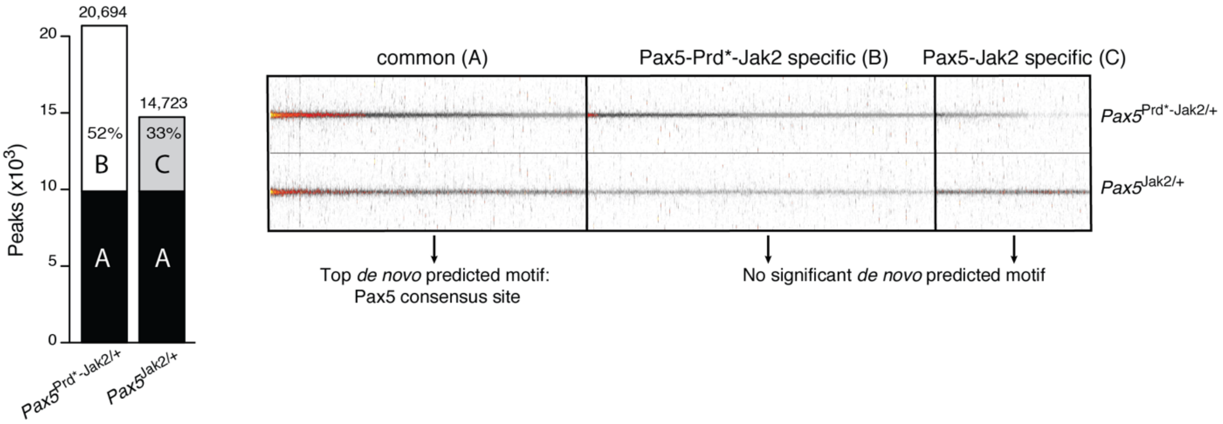

Figure 3. The number of common and unique peaks of the *Pax5*$^{Prd*-Jak2/+}$ and *Pax5*$^{Jak2\ +}$ B-ALL cells (shown in Figure S5I) are shown to the left and the density of Pax5-Jak2 binding is shown to the right.

4) The authors claim that the kinase domain is required for the oncogenic function of Pax5Jak2. This conclusion is most likely correct. However, the experiments supporting this claim should be interpreted with some caution. The genetic data involve a mouse carrying a point mutation in the kinase domain of Pax5Jak2. Based on the immunoblot analysis shown in Fig. S4B, the mutant fusion protein is expressed at a four-fold lower level than the corresponding PAX5-JAK2 protein. In other experiments, the authors find that the dosage of PAX5-JAK2 and PAX5 influences the penetrance and initiation of leukemia. Therefore, the authors may want to temper their conclusion. The effect of pharmacological inhibition of the kinase domain by treatment of cells with the JAK1/2 inhibitor Ruxolitinib is clear. However, the authors should mention that the inhibitor may also target general JAK1/2 function and not only that of the PAX5-JAK2 fusion protein.

1) As suggested by the reviewer, we now mention the potential limitation caused by the lower expression of the Pax5-Jak2-KD protein in *Pax5*$^{Jak2-KD/+}$ B cells (Figure S4B) by making the following statement on page 16 (top): “While the 4-fold lower expression of the Pax5-Jak2-KD protein in pro-B cells is expected to significantly delay the tumor onset, the complete absence of

B-ALL in 1-year-old *Pax5*$^{Jak2\text{-}KD/+}$ mice nevertheless indicates a critical role of the Pax5-Jak2 kinase activity in the initiation of leukemia development".

2) At the first appearance, we have introduced ruxolitinib as a JAK1/2 inhibitor on page 16 (bottom) in the result section and in the legend of Figure 4D,E (page 42) of the initial submission. We now added the following sentence on page 16 to indicate that ruxolitinib can also inhibit endogenous Jak1: "As pro-B and pre-B cells abundantly express Jak1 but not Jak2 (ImmGen database), ruxolitinib may mediate its effect by inhibition of the endogenous Jak1, transgenic Pax5-Jak2 or both kinases in *Pax5*$^{Jak2/+}$ B-ALL tumors".

5) The manuscript would also benefit from shortening. The result section contains description of experimental details (including generation of mouse strains) that could be moved to the Materials and Methods section: Likewise, the entire Fig. 6 and the corresponding result section is dedicated to negative data concerning the lack of epigenetic regulation by Pax5Jak2. Therefore, I suggest to include this figure in Supplemental Figures and to shorten the description in the text accordingly.

1) Describing the generation of the *Pax5*$^{Jak2/+}$ mouse is central for the understanding of the Pax5-Jak2 tumor model for the following reasons. The high aggressiveness of the B-ALL tumor resulted in the death of *Pax5*$^{Jak2/+}$ mice within 2.5 months (Figure 1F). Consequently, we would have lost the *Pax5*$^{Jak2/+}$ mouse strain already in the first generation. To prevent this, we had to use a complicated conditional strategy by introducing a floxed stop cassette (LSL) upstream of the *Jak2* cDNA insertion in the *Pax5*$^{LSL\text{-}Jak2}$ allele. Every experimental *Pax5*$^{Jak2/+}$ mouse was thereafter generated by deletion of the LSL cassette by the ubiquitous Meox2-Cre line. We therefore had to describe the entire experimental strategy in the first paragraph of the result section. In our opinion, the clarity of the manuscript would have suffered by relegating the entire description to Materials and Methods

2) The issue of H3Y41 phosphorylation has been an unresolved and hotly debated issue in both the epigenetics and JAK-STAT communities. We therefore think that we should not hide the data in a supplementary figure, especially when we have an entire paragraph dedicated to the phosphorylation of H3Y41 and even mention these epigenetic data in the abstract. Sometimes, it is also important to show well-controlled negative data. Finally, we have generated a monoclonal anti-H3Y41ph antibody and could demonstrate with this new tool that this histone modification does not exist. As this message will be lost by relegation of the data to a supplementary figure, we strongly argue for showing these data in Figure 6.

6) The authors may want to mention recent studies showing that combined Pax5 and Ebf1 heterozygosity in the mouse also enhances IL-7R signaling and STAT5 phosphorylation in B-ALL.

As suggested by the reviewer, we now mention the finding of Ramamoorthy et al (2020) in the context of the IL-7 sensitivity of the *Pax5*$^{Jak2/+}$ B-ALL cells on page 23 (top).

Referee #3:

In the manuscript entitled "PAX5-JAK2 acts as a dual-hit mutation to induce B cell leukemia by regulating nuclear STAT5 activity", the authors created a mouse model to analyze the role of PAX5-JAK2 (PJ) fusion protein in developing B-ALL in vivo. The mice expressing PJ fusion protein from endogenous PAX5 locus develop very aggressive B-ALL with median survival of 74 days. The DNA binding activity of PJ and its ability to constitutively phosphorylate Stat5 are found

to be responsible for the observed phenotype. In addition, due to acquired uniparental disomy (aUPD) observed in the mice, they lose the WT PAX5 allele in the malignant B-ALL cells which accelerates the leukemia development. Although the study generates a new mouse model and characterized it, there are several questions to the model as well as the experimental results and interpretation.

Specific points:

1. The authors expressed PJ fusion gene from the endogenous PAX5 locus to closely mimic the disease situation. However, the construct led to the loss of WT PAX5 allele which is not observed in the disease. The data provided by the authors about the human B-ALL cells also supports this fact. Thus this mouse line does not actually model the human disease. It is difficult to distinguish the alterations that is caused by the oncogenic fusion protein from those caused due to loss WT PAX5 allele. From this point of view, the Cd79a-Cre Ikzf1neo/+ Pax5LSL-Jak2/+ line is a better choice of model. The longer latency period in these mice indicates that Pax5Jak2/+ model is indeed an exaggerated model for B-ALL not because of the fusion protein itself but due to model design.

We have explained in detail the differences between the mouse *Pax5*$^{Jak2/+}$ B-ALL model and the human PAX5-JAK2^{+} B-ALL tumors and discussed the limitations of the *Pax5*$^{Jak2/+}$ model on page 28 of the discussion section. On the positive side, the Pax5 loss in the *Pax5*$^{Jak2/+}$ model allowed us to study the genome-wide binding of Pax5-Jak2, which would not have been possible in the 'better' *Cd79a*-Cre *Ikzf1*$^{neo/+}$ *Pax5*$^{LSL\text{-}Jak2/+}$ B-ALL model or even in human PAX5-JAK2^{+} B-ALL tumors due to the DNA-binding competition by the wild-type Pax5 protein. Moreover, the *Pax5*$^{Jak2/+}$ model also allowed us to decipher the role of Pax5-Jak2 in maintaining the level of active phosphorylated STAT5 in the nucleus (see point 9 below for detailed explanation). The *Cd79a*-Cre *Ikzf1*$^{neo/+}$ *Pax5*$^{LSL\text{-}Jak2/+}$ mice were important to demonstrate that the Pax5 loss contributes to tumor acceleration but that it is not required for B-ALL development. Most importantly, the work that we present in this manuscript could not have been performed with the *Cd79a*-Cre *Ikzf1*$^{neo/+}$ *Pax5*$^{LSL\text{-}Jak2/+}$ tumor model, as we could have never generated enough mice of this complicated genotype for a comprehensive study. In addition, the analysis of these mice would have been further complicated by the relatively long tumor latency and incomplete penetrance (Figure 3G).

2. The major focus of the study is to show the oncogenic potential of the PJ fusion in the development of B-ALL in mouse model. The oncogenic potential of PJ fusion for B-ALL is already known and the present study does not add any additional information regarding the mechanism of action. The authors did several high-throughput sequencing studies including RNA-seq and ChIP-seq. But they did not pinpoint clearly the cellular processes that are altered due to PJ fusion. They have shown the change in expression for some genes (Figure 2E, G), but what is the relevance of these genes in the context of B-ALL? Except for the explanation why B220 is lowered in the ALL cells, no further explanation has been given. From their expression data, can the authors deduce a mechanism how after 3 weeks of age the pro-B cells rapidly transform into the malignant ALL cells?

In response to the reviewer's question, we would like to emphasize our important finding that Pax5-Jak2 does not function as a transcription factor (Figure S2B,C) but instead is a DNA-binding, constitutively active Jak2 kinase that phosphorylates STAT5 in the nucleus. To further corroborate this finding, we have performed new experiments to interrogate the phosphorylation status of STAT5 in bone marrow B cells of *Pax5*$^{Jak2/+}$ mice at the age of 4-5 weeks (shown in the new

Figures 7F). These new data demonstrate that there is a small but significant increase of p-STAT5 levels in *Pax5*$^{Jak2/+}$ pro-B cells compared with wild-type pro-B cells *in vivo*. Importantly however, a strong increase of the p-STAT5 level was already seen in the leukemic B220^{low} B cells at the age of 4-5 weeks concomitant with the loss of the wild-type *Pax5* allele and increased DNA-binding of Pax5-Jak2. As active STAT5 is known to be an oncogenic driver of B-ALL development, we consider the phosphorylation of STAT5 in the nucleus to be the main oncogenic ('cellular') function of Pax5-Jak2.

3. The authors compared the gene expression alteration by PJ fusion protein always with respect to another B-ALL model and stressed on the PAX5 activated and repressed genes. It is well possible that the fusion protein has its own specific gene expression paradigm other than deregulating the PAX5 regulated genes. Can the authors compare the gene expression changes with respect to WT to find out these genes? This should be performed in the model expressing PJ and ectopic PAX5 as this circumvents the problem of WT PAX5 loss in the Pax5Jak2/+ model.

1) The GSEA analysis of Figure 2D,F may have overemphasized the contribution of Pax5-regulated genes to the differential gene expression signature of *Pax5*$^{Jak2/+}$ B-ALL cells, as only 27% of the downregulated genes and 30% of the upregulated genes correspond to activated and repressed Pax5 target genes, respectively, which we now mention on page 10. Hence, 70% of the differentially expressed genes in *Pax5*$^{Jak2/+}$ B-ALL cells are regulated independently of Pax5.

2) As described in the manuscript and mentioned above (under point 2), Pax5-Jak2 is not a transcription factor and therefore cannot turn on its own gene expression signature. However, Pax5-Jak2 phosphorylates another transcription factor, STAT5, in the nucleus. In this sense, the 'expression paradigm' of Pax5-Jak2 is to activate STAT5 that, in turn, switches on its own STAT5-dependent gene expression program (Figure 7).

3) We did not perform RNA-sequencing of *Cd79a*-Cre *Ikzf1*$^{neo/+}$ *Pax5*$^{LSL\text{-}Jak2/+}$ B-ALL cells and can therefore not perform the requested gene expression comparison. Moreover, the generation of these data would have delayed the revision of our manuscript by several months due to the inefficient generation of *Cd79a*-Cre *Ikzf1*$^{neo/+}$ *Pax5*$^{LSL\text{-}Jak2/+}$ mice, the long latency of the respective tumors and the time required for RNA-sequencing and bioinformatic analysis.

4. The authors concluded that the JAK2 kinase activity is required for the ALL development as the kinase dead (KD) construct leads to delay of ALL onset. However, the expression level of PJ-KD fusion is extremely low compared to PJ making it difficult to understand if the observed delay is due to loss of kinase activity of JAK2 or simply due to low expression of the oncogene. Notably, this is already the case (as also mentioned by the authors) for the Pax5Jak2-Luc/+ mice that express 2 fold less fusion protein and consequently develops ALL at 3 fold longer latency.

As suggested by the reviewer, we now mention the potential limitation caused by the lower expression of the Pax5-Jak2-KD protein in *Pax5*$^{Jak2\text{-}KD/+}$ B cells (Figure S4B) by making the following statement on page 16 (top): "While the 4-fold lower expression of the Pax5-Jak2-KD protein in pro-B cells is expected to significantly delay the tumor onset, the complete absence of B-ALL in 1-year-old *Pax5*$^{Jak2\text{-}KD/+}$ mice nevertheless indicates a critical role of the Pax5-Jak2 kinase activity in the initiation of leukemia development".

5. The authors show that treatment with JAK2 inhibitor Ruxolitinib prolongs the survival of the mouse by 20 days. There is no statistical analysis given for this observation (Fig 4E-F). How significant is this change? It seems that this small change also indicates that longer latency of ALL onset in kinase dead expressing mice is actually due to low expression of the oncogene.

1) We have determined and added the statistical significance for the ruxolitinib time course (Figure 4E) and survival curve (Figure 4F), as suggested by the reviewer.

2) Ruxolitinib treatment determines the role of Pax5-Jak2 activity during the tumor maintenance phase, while the kinase-dead mutation (K272E) of Pax5-Jak2 provides information about the role of the Pax5-Jak2 activity during the initiation of tumor development. As the functions of Pax5-Jak2 in tumor initiation and maintenance are likely different, it may not be valid to conclude from the ruxolitinib data, what role the Pax5-Jak2 protein could play during the tumor initiation phase.

6. Using ChIP-seq analysis, the authors analyzed the DNA binding ability of the PJ fusion protein. It is really surprising that they only concentrated on the numerical values of the binding sites occupied by PJ fusion or the paired domain mutant. Why do they not analyze the genes/ regulatory regions belonging to site B (specific for PJ fusion)? This might provide more valuable information about the oncogenic mechanism of the fusion protein. The five mutations in the PAX5 Prd domain do not eliminate DNA binding but lead to increased latency of the disease onset. It is therefore important to analyze this differential DNA binding between Prd and PJ to understand how PJ leads to such aggressive disease course. Such analysis is completely missing in the paper.

1) In answering the question about the 'genes/regulatory regions' (like site B), we would like to point out that Pax5-Jak2 is a DNA-binding Jak2 kinase and not a transcription factor. Moreover, the Pax5-Jak2-specific peaks in the B-ALL cells have a very low density in general, which indicates weak binding (Figure S5D; including site B, Figure S5A). The low density of these Pax5-Jak2-specific peaks (Figure S5D) does not warrant a detail analysis. Moreover, the Pax5-Jak2-specific peaks may be indirectly caused by differential chromatin accessibility of these sites in the tumor cells compared to pro-B cells.

2) As suggested by the reviewer, we have further analyzed the ChIP-seq data of the *Pax5*$^{\text{Prd*-Jak2/+}}$ and *Pax5*$^{\text{Jak2 +}}$ B-ALL cells (Figure S5I of the manuscript). Figure 3 (for reviewer) shows the density of the common (A), Pax5-Prd*-Jak2-specific (B) and Pax5-Jak2-specific (C) peaks. While the *de novo* motif discovery program MEME-ChIP readily identified the consensus Pax5 recognition motif in the common peaks (A), we could not find any significant *de novo*-predicted motif in the Pax5-Prd*-Jak2-specific (B) and Pax5-Jak2-specific (C) peaks. Unfortunately, these analyses could not reveal a potentially new function for the Pax5-Jak2-specific peaks. As a word of caution, it may be difficult to establish a 1:1 correlation between DNA-binding and an aggressive disease for a DNA-binding Jak2 kinase that does not function as a transcription factor.

7. Regarding the epigenetic function of PJ, it is difficult to assess the data as the antibody generated in the study does not detect H3Y41Ph in cell lines previously reported to be positive for this mark. Therefore, the lack of H3Y41ph in PJ expressing cells could be due to antibody problem as well. Additionally, why did they suddenly use Pax5Jak2/- cells for the ChIP seq analysis? These cells are not even committed to B cell lineage. Thus, the Pax5Jak2/- cells and the tumor cells are temporally in different state.

1) There must be a misunderstanding here. The scientific debate at the start of our experiments was whether H3Y41 phosphorylation (as described by Dawson et al., 2009) exists as a histone modification or not. As the original rabbit polyclonal anti-H3Y41ph antibody was no longer available for many years, we generated a new monoclonal anti-H3Y41ph antibody and demonstrated unequivocally that this antibody recognizes the phosphorylated Y41 residue in the context of the H3 sequence (Figure 6B,C). The question then was whether we could detect the

H3Y41ph mark with this new antibody in *Pax5*$^{Jak2/+}$ B-ALL cells and the three cell lines (HEL, TMD8, K1106), where H3Y41 phosphorylation was apparently detected in the study of Dawson et al. (2009). Although we could readily detect an H3pY41 peptide coupled to ubiquitin by immunoblot analysis with our monoclonal antibody, we could not detect any phosphorylation of Y41 on endogenous H3 proteins in the three cell lines and *Pax5*$^{Jak2/+}$ B-ALL cells. We therefore conclude that H3Y41ph does not exist as a histone modification.

2) We apologize for not having more explicitly explained why we used *Pax5*$^{Jak2/–}$ and *Pax5*$^{Prd/–}$ progenitors for investigating a possible effect of Pax5-Jak2 on the generation of active chromatin by ChIP-seq analysis. As both *Pax5*$^{Jak2/–}$ and *Pax5*$^{Prd/–}$ progenitors are arrested at the same uncommitted cell stage, we could directly compare the chromatin profiles between these two cell types. This analysis could not be carried out with *Pax5*$^{Jak2/+}$ B-ALL cells, as no control reference cell type for these B-ALL cells exists. We now explain in detail on page 22 why we used the *Pax5*$^{Jak2/–}$ and *Pax5*$^{Prd/–}$ progenitors for ChIP-seq analysis.

8. The loss of IL7 receptor also increases disease latency. However, no statistics is provided here as well (Fig 7C and S7C). How significant is the observation? Can the authors also see alteration of IL7 regulated genes in the PJ expressing B-ALL cells in their high-throughput sequencing analysis?

1) As suggested by the reviewer, we now provide a statistical analysis of the data shown in Figures 7C and S7C, which were obtained by transplantation of *Pax5*$^{Jak2/+}$ B-ALL cells into *Il7* mutant recipient mice, but not into IL-7α receptor-deficient recipient mice.

2) A prominent downstream transcription factor of IL-7R signaling is STAT5, whose role we have investigated in Figures 7D-K and S7G-I.

9. The increased phosphorylation of Stat5 by PJ seems to be an over-estimation. The elevated pStat5 in the tumor cells is clearly evident but the increase shown in follicular (Fig 7F, G) or Pro B cells (Fig S7D, E) is really week to none. Specifically, the representative histogram plot shown in Fig S7D should not have a statistical significance shown in S7E. The authors also proposed that PJ fusion protein keeps Stat5 in constitutively phosphorylated form leading to activation of the Stat5 regulated genes. However, they did not show this constitutive activation. Can they show that in PJ expressing cells Stat5 is solely nuclear and constitutively phosphorylated?

1) We agree with the reviewer that the minimal increase of p-STAT5 in *Pax5*$^{Jak2/+}$ FO B cells compared with *Pax5*$^{+/+}$ FO B cells may not be biologically relevant, although it is statistically significant (Figures 7E and S7F). To investigate when p-STAT5 levels are increased during leukemia development, we performed new experiments by analyzing B cells from the bone marrow of *Pax5*$^{Jak2/+}$ mice at the age of 4-5 weeks (new Figures 7F, mentioned already under point 2). These new data also demonstrate a small increase of p-STAT5 levels in *Pax5*$^{Jak2/+}$ pro-B cells compared with wild-type pro-B cells in vivo. Importantly, the p-STAT5 levels are strongly increased in the leukemic B220^{low} B cells at the age of 4-5 weeks. We conclude therefore that the strong increase of STAT5 phosphorylation occurs in early leukemogenesis concomitant with the loss of the wild-type *Pax5* allele and increased DNA-binding of Pax5-Jak2. These data provide strong support for a nuclear function in maintaining high levels of p-STAT5 in *Pax5*$^{Jak2/+}$ B-ALL cells. As active STAT5 is known to be an oncogenic driver of B-ALL development, we consider the phosphorylation of STAT5 in the nucleus to be the main oncogenic function of Pax5-Jak2. This new experiment is mentioned on page 24 (result) and 30 (discussion).

2) While we have demonstrated that the Pax5-Jak2 protein is almost exclusively present in the nucleus of *Pax5*$^{Jak2/+}$ B-ALL cells (Figure S6A), we have not analyzed the nuclear location of p-STAT5, which is known to be predominantly localized in the nucleus, as recently reviewed by Villarino et al. (2017. *Nat. Immunol.* 18:374-384) and Wingelhofer et al. (2018. *Leukemia* 32:1713-1726).

10. The authors mentioned in the introduction that "Pax5Jak2/+ mice exhibited normal B cell development up to 3 weeks of age, but thereafter rapidly developed an aggressive B-ALL tumor in the absence of another cooperating gene mutation". The absence of other mutation is not actually tested in the study. The latency of B-ALL in the current model is too short. However, did they test any additional mutation in the ectopic PAX5 expressing mouse line?

We generated and analyzed the *Cd79a*-Cre *Ikzf1*$^{neo/+}$ *Pax5*$^{LSL\text{-}Jak2/+}$ mice to see whether ectopic expression of Pax5 from the *Ikzf1* locus could delay B-ALL development, which it did. The longer latency is best explained by the *de novo* generation of cooperating gene mutations that promote tumor progression, as the reviewer correctly suggests. However, it is beyond the interest and focus of this manuscript to identify these mutations by whole-exome or whole-genome sequencing.

Additional figure changes

1. Minor change in numbers of differentially expressed genes shown in Figure 2C. We noticed only during the revision that a few V_H gene segments of the *Igh* locus were present among the differentially expressed genes shown in Figures 2C and S2A. As V_H gene segment cannot be considered to be a real gene, they have been eliminated from the gene lists (Tables S2 and S3), and the numbers of differentially expressed genes have been accordingly corrected in Figures 2C and S2A.

2. Swapping the two experiments demonstrating the IL-7 dependence of *Pax5*$^{Jak2/+}$ B-ALL tumors between Figure 7B and Figure S7C. Unfortunately, we have only now realized that the data of the previous Figure S7C correspond to the bioluminescence experiment shown in Figure 7A. We have corrected this mistake by swapping the data between Figure 7B and Figure S7C in the revised figures.

1st Revision - Editorial Decision 16th Dec 2021

Dear Meinrad,

Thank you for submitting your revised manuscript to The EMBO Journal. Your revision has now been seen by the three original referees. As you can see below, they appreciate the introduced revisions and have no further points.

I am therefore very pleased to accept your manuscript for publication here. Before I can send you the formal acceptance letter there are just a few editorial points to sort out. You can use the link below to upload the revised manuscript.

- Please upload individual high resolution figure files.

- We are missing 3-5 keywords

- Is there a good reason for not depositing the human RNA-seq data in a database?

- Please double check that all the funding is entered in both the submission system and manuscript. (Austrian Research Promotion Agency (Early Stage Grant 'Molecular Control' FFG-878286) and an EMBO fellowship (ALTF 140-2013))

- Competing financial interests should be re-labelled as Conflict of interest

- For the reference list: for articles with more than 10 authors, the author list should be cut after 10 authors followed by et al.

- Tables S1-4 should be re-named Dataset EV1, EV2.... Please also correct callout in text.

- The supplemental figures should re-named as Appendix. The appendix should also have a ToC and correct nomenclature in figure files to "Appendix Figure S1" etc. Please also correct callout in text. Alternative, you can also remove some of the figures from the appendix and upload them as expanded view figures. For more information see https://www.embopress.org/page/journal/14602075/authorguide#expandedview

- We don't allow data not shown (p.8, 12, 14, 20, 22). Please take a look and re-phrase.

- Place the Add M&M after Discussion

- For figure 3F and 7I, J the statistical analysis is based upon n=2. We normally discourage statistical analysis when N=2. Can we discuss how to display the data points best?

- Our publisher has also done their pre-publication check on your manuscript. When you log into the manuscript submission system you will see the file "Data Edited Manuscript file". Take a look at the word file and the comments regarding the figure legends and respond to the issues.

- We encourage the publication of source data, particularly for electrophoretic gels and blots, with the aim of making primary data more accessible and transparent to the reader. It would be great if you could provide me with a PDF file per figure that contains the original, uncropped and unprocessed scans of all or key gels used in the figure? The PDF files should be labelled with the appropriate figure/panel number, and should have molecular weight markers; further annotation could be useful but is not essential. The PDF files will be published online with the article as supplementary "Source Data" files.

- We include a synopsis of the paper (see http://emboj.embopress.org/). Please provide me with a general summary statement and 3-5 bullet points that capture the key findings of the paper.

- We also need a summary figure for the synopsis. The size should be 550 wide by [200-400] high (pixels). You can also use something from the figures if that is easier.

That should be all! Let me know if you have any questions

With best wishes

Karin

Karin Dumstrei, PhD
Senior Editor
The EMBO Journal

Guide For Authors: https://www.embopress.org/page/journal/14602075/authorguide

We realize that it is difficult to revise to a specific deadline. In the interest of protecting the conceptual advance provided by the work, we recommend a revision within 3 months (16th Mar 2022). Please discuss the revision progress ahead of this time with the editor if you require more time to complete the revisions. Use the link below to submit your revision:

https://emboj.msubmit.net/cgi-bin/main.plex

Referee #1:

The authors have addressed my concerns in a most satisfying way.

Referee #2:

The authors have addressed the comments in a satisfactory manner and have further strengthened an interesting manuscript.

Referee #3:

The authors have satisfactorily answered the initially raised questions. The revised manuscript contains new data and explains the strengths of the mouse model and the differences to human disease.

Karin Dumstrei, PhD
Senior Editor
The EMBO Journal

Vienna, 31 December 2021

Submission of the edited manuscript EMBOJ-2021-108397R (Jurado et al.)

Dear Karin,

Enclosed, we submit our edited revised manuscript. We made the requested modifications.

- We added in the legends the additional statistical data requested by the editor.
- We reduced the keywords to 5 on the front page
- I have double-checked that the funding sources are correctly entered in the submission system.
- The conflict-of-interest statement is now consistent with the EMBO J. style.
- We have renamed Tables S1-S6 as Tables EV1-EV6 and Fig S1-S7 as Appendix Fig S1-S7.
- We have eliminated all "data not shown" statements.
- We have added an abbreviated Methods section after Discussion.
- We have generated an Appendix file (pdf) containing the Appendix Fig S1-S7 and Appendix Supplementary Methods.
- We now submit all the requested Data Source files.
- We now submit a synopsis figure and synopsis text.
- We submit Figures 1-8 as high-quality pdf files.
- We also submit the Tables EV1-EV6 again after correctly relabeling them.
- We submitted the edited revised manuscript text as a Word document.

We are not allowed to deposit the human RNA-seq data in a public database, as the informed consent does not allow this, because no data transfer agreement was signed at the time. We therefore mention in the Data Availability statement that "the human RNA-seq data are available upon request".

Let me know whether everything is now correct for further processing of our manuscript

With best wishes for a successful 2022,

Meinrad Busslinger

Dear Meinrad,

Thank you for submitting your revise manuscript. I have now had a chance to look at it and all looks good!

I am therefore very pleased to accept the manuscript for publication here.

With best wishes

Karin

Karin Dumstrei, PhD
Senior Editor
The EMBO Journal

Please note that it is EMBO Journal policy for the transcript of the editorial process (containing referee reports and your response letter) to be published as an online supplement to each paper. If you do NOT want this, you will need to inform the Editorial Office via email immediately. More information is available here:
https://www.embopress.org/page/journal/14602075/authorguide#transparentprocess

Your manuscript will be processed for publication in the journal by EMBO Press. Manuscripts in the PDF and electronic editions of The EMBO Journal will be copy edited, and you will be provided with page proofs prior to publication. Please note that supplementary information is not included in the proofs.

Please note that you will be contacted by Wiley Author Services to complete licensing and payment information. The 'Page Charges Authorization Form' is available here: https://www.embopress.org/pb-assets/embo-site/tej_apc.pdf

Should you be planning a Press Release on your article, please get in contact with embojournal@wiley.com as early as possible, in order to coordinate publication and release dates.

If you have any questions, please do not hesitate to call or email the Editorial Office. Thank you for your contribution to The EMBO Journal.

** Click here to be directed to your login page: https://emboj.msubmit.net

EMBO PRESS

YOU MUST COMPLETE ALL CELLS WITH A PINK BACKGROUND ↓

PLEASE NOTE THAT THIS CHECKLIST WILL BE PUBLISHED ALONGSIDE YOUR PAPER

Corresponding Author Name:	Meinrad Busslinger
Journal Submitted to:	EMBO Journal
Manuscript Number:	EMBOJ-2021-108397

USEFUL LINKS FOR COMPLETING THIS FORM

http://www.antibodypedia.com
http://1degreebio.org
http://www.equator-network.org/reporting-guidelines/improving-bioscience-research-repor
http://grants.nih.gov/grants/olaw/olaw.htm
http://www.mrc.ac.uk/Ourresearch/Ethicsresearchguidance/Useofanimals/index.htm
http://ClinicalTrials.gov
http://www.consort-statement.org
http://www.consort-statement.org/checklists/view/32-consort/66-title
http://www.equator-network.org/reporting-guidelines/reporting-recommendations-for-tumo
http://datadryad.org
http://figshare.com
http://www.ncbi.nlm.nih.gov/gap
http://www.ebi.ac.uk/ega
http://biomodels.net/
http://biomodels.net/miriam/
http://jjj.biochem.sun.ac.za
https://osp.od.nih.gov/biosafety-biosecurity-and-emerging-biotechnology/
http://www.selectagents.gov/

Reporting Checklist For Life Sciences Articles (Rev. June 2017)

This checklist is used to ensure good reporting standards and to improve the reproducibility of published results. These guidelines are consistent with the Principles and Guidelines for Reporting Preclinical Research issued by the NIH in 2014. Please follow the journal's authorship guidelines in preparing your manuscript.

A- Figures
1. Data
The data shown in figures should satisfy the following conditions:

- ➔ the data were obtained and processed according to the field's best practice and are presented to reflect the results of the experiments in an accurate and unbiased manner.
- ➔ figure panels include only data points, measurements or observations that can be compared to each other in a scientifically meaningful way.
- ➔ graphs include clearly labeled error bars for independent experiments and sample sizes. Unless justified, error bars should not be shown for technical replicates.
- ➔ if n< 5, the individual data points from each experiment should be plotted and any statistical test employed should be justified
- ➔ Source Data should be included to report the data underlying graphs. Please follow the guidelines set out in the author ship guidelines on Data Presentation.

2. Captions
Each figure caption should contain the following information, for each panel where they are relevant:

- ➔ a specification of the experimental system investigated (eg cell line, species name).
- ➔ the assay(s) and method(s) used to carry out the reported observations and measurements
- ➔ an explicit mention of the biological and chemical entity(ies) that are being measured.
- ➔ an explicit mention of the biological and chemical entity(ies) that are altered/varied/perturbed in a controlled manner.

- ➔ the exact sample size (n) for each experimental group/condition, given as a number, not a range;
- ➔ a description of the sample collection allowing the reader to understand whether the samples represent technical or biological replicates (including how many animals, litters, cultures, etc.).
- ➔ a statement of how many times the experiment shown was independently replicated in the laboratory.
- ➔ definitions of statistical methods and measures:
  - common tests, such as t-test (please specify whether paired vs. unpaired), simple χ^2 tests, Wilcoxon and Mann-Whitney tests, can be unambiguously identified by name only, but more complex techniques should be described in the methods section;
  - are tests one-sided or two-sided?
  - are there adjustments for multiple comparisons?
  - exact statistical test results, e.g., P values = x but not P values < x;
  - definition of 'center values' as median or average;
  - definition of error bars as s.d. or s.e.m.

Any descriptions too long for the figure legend should be included in the methods section and/or with the source data.

In the pink boxes below, please ensure that the answers to the following questions are reported in the manuscript itself. Every question should be answered. If the question is not relevant to your research, please write NA (non applicable). We encourage you to include a specific subsection in the methods section for statistics, reagents, animal models and human subjects.

B- Statistics and general methods

Please fill out these boxes ↓ (Do not worry if you cannot see all your text once you press return)

1.a. How was the sample size chosen to ensure adequate power to detect a pre-specified effect size?	Information about the sample size and number of independent experiments is provided in the respective figure legends and materials and methods section.
1.b. For animal studies, include a statement about sample size estimate even if no statistical methods were used.	Information about the number of mice used for each experiment is provided in the respective figure legends.
2. Describe inclusion/exclusion criteria if samples or animals were excluded from the analysis. Were the criteria pre-established?	The data of all mice analyzed were included in the respective analyses.
3. Were any steps taken to minimize the effects of subjective bias when allocating animals/samples to treatment (e.g. randomization procedure)? If yes, please describe.	No randomization procedure was used for the experiments described in this manuscript.
For animal studies, include a statement about randomization even if no randomization was used.	No randomization procedure was used for the experiments described in this manuscript.
4.a. Were any steps taken to minimize the effects of subjective bias during group allocation or/and when assessing results (e.g. blinding of the investigator)? If yes please describe.	No blinding of the investigator was used for carrying out the experiments, except for the assessment of sick mice, which was done by an animal technician who did not know the genotypes of the mice.
4.b. For animal studies, include a statement about blinding even if no blinding was done	No blinding of the investigator was used for carrying out the experiments.
5. For every figure, are statistical tests justified as appropriate?	For each figure, the statistical tests used were justified as appropriate and are mentioned in the figure legends.
Do the data meet the assumptions of the tests (e.g., normal distribution)? Describe any methods used to assess it.	The statistical tests used to analyze the experimental data are mentioned in in the figure legends and met the assumption of the respective tests.
Is there an estimate of variation within each group of data?	We have included the experimental variation as standard error of the mean (SEM), when displaying the experimental data in the figures.

Is the variance similar between the groups that are being statistically compared?	Yes

C- Reagents

6. To show that antibodies were profiled for use in the system under study (assay and species), provide a citation, catalog number and/or clone number, supplementary information or reference to an antibody validation profile. e.g., Antibodypedia (see link list at top right), 1DegreeBio (see link list at top right).	Detailed information about all antibodies including their clone number are provided in materials and methods.
7. Identify the source of cell lines and report if they were recently authenticated (e.g., by STR profiling) and tested for mycoplasma contamination.	No commercially available cell lines were used for our study.

* for all hyperlinks, please see the table at the top right of the document

D- Animal Models

8. Report species, strain, gender, age of animals and genetic modification status where applicable. Please detail housing and husbandry conditions and the source of animals.	We provided all relevant information about the mice used in material and methods.
9. For experiments involving live vertebrates, include a statement of compliance with ethical regulations and identify the committee(s) approving the experiments.	All animal experiments were carried out according to valid project licenses, which were approved and regularly controlled by the Austrian Veterinary Authorities.
10. We recommend consulting the ARRIVE guidelines (see link list at top right) (PLoS Biol. 8(6), e1000412, 2010) to ensure that other relevant aspects of animal studies are adequately reported. See author guidelines, under 'Reporting Guidelines'. See also: NIH (see link list at top right) and MRC (see link list at top right) recommendations. Please confirm compliance.	Our mouse experiments were conducted in compliance with these guidelines.

E- Human Subjects

11. Identify the committee(s) approving the study protocol.	The use of the human tumor samples was approved by the respective Insitutional Review Boards of St Jude Children's Research Hospital (Memphis, USA) and the St. Anna Children's Cancer Research Institute (Vienna, Austria)
12. Include a statement confirming that informed consent was obtained from all subjects and that the experiments conformed to the principles set out in the WMA Declaration of Helsinki and the Department of Health and Human Services Belmont Report.	Informed consent was obtained from all 8 patients for analyzing their B-ALL cells by RNA-sequencing.
13. For publication of patient photos, include a statement confirming that consent to publish was obtained.	Not applicabile.
14. Report any restrictions on the availability (and/or on the use) of human data or samples.	The RNA-seq data of the human B-ALL samples generated will not be publically availabe, as they will be provided only upon request.
15. Report the clinical trial registration number (at ClinicalTrials.gov or equivalent), where applicable.	Not applicabile.
16. For phase II and III randomized controlled trials, please refer to the CONSORT flow diagram (see link list at top right) and submit the CONSORT checklist (see link list at top right) with your submission. See author guidelines, under 'Reporting Guidelines'. Please confirm you have submitted this list.	Not applicabile.
17. For tumor marker prognostic studies, we recommend that you follow the REMARK reporting guidelines (see link list at top right). See author guidelines, under 'Reporting Guidelines'. Please confirm you have followed these guidelines.	Not applicabile.

F- Data Accessibility

18: Provide a "Data Availability" section at the end of the Materials & Methods, listing the accession codes for data generated in this study and deposited in a public database (e.g. RNA-Seq data: Gene Expression Omnibus GSE39462, Proteomics data: PRIDE PXD000208 etc.) Please refer to our author guidelines for 'Data Deposition'.<br><br>Data deposition in a public repository is mandatory for:<br>a. Protein, DNA and RNA sequences<br>b. Macromolecular structures<br>c. Crystallographic data for small molecules<br>d. Functional genomics data<br>e. Proteomics and molecular interactions	All mouse NGS data generated in this study have been submitted to the Gene Expression Omnibus (GEO) repository under the accession number GSE174775, as mentioned under "Data Avaiablility".
19. Deposition is strongly recommended for any datasets that are central and integral to the study; please consider the journal's data policy. If no structured public repository exists for a given data type, we encourage the provision of datasets in the manuscript as a Supplementary Document (see author guidelines under 'Expanded View' or in unstructured repositories such as Dryad (see link list at top right) or Figshare (see link list at top right).	All mouse NGS data generated in this study have been submitted to the Gene Expression Omnibus (GEO) repository under the accession number GSE174775, as mentioned under "Data Avaiablility".
20. Access to human clinical and genomic datasets should be provided with as few restrictions as possible while respecting ethical obligations to the patients and relevant medical and legal issues. If practically possible and compatible with the individual consent agreement used in the study, such data should be deposited in one of the major public access-controlled repositories such as dbGAP (see link list at top right) or EGA (see link list at top right).	The RNA-seq data of the human B-ALL samples generated will not be publically availabe, as they will be provided only upon request.
21. Computational models that are central and integral to a study should be shared without restrictions and provided in a machine-readable form. The relevant accession numbers or links should be provided. When possible, standardized format (SBML, CellML) should be used instead of scripts (e.g. MATLAB). Authors are strongly encouraged to follow the MIRIAM guidelines (see link list at top right) and deposit their model in a public database such as Biomodels (see link list at top right) or JWS Online (see link list at top right). If computer source code is provided with the paper, it should be deposited in a public repository or included in supplementary information.	Not applicabile.

G- Dual use research of concern

22. Could your study fall under dual use research restrictions? Please check biosecurity documents (see link list at top right) and list of select agents and toxins (APHIS/CDC) (see link list at top right). According to our biosecurity guidelines, provide a statement only if it could.	Not applicabile.
